# A non‐canonical scaffold‐type E3 ligase complex mediates protein UFMylation

**DOI:** 10.15252/embj.2022111015

**Published:** 2022-09-19

**Authors:** Joshua J Peter, Helge M Magnussen, Paul A DaRosa, David Millrine, Stephen P Matthews, Frederic Lamoliatte, Ramasubramanian Sundaramoorthy, Ron R Kopito, Yogesh Kulathu

**Affiliations:** ^1^ Medical Research Council Protein Phosphorylation & Ubiquitylation Unit (MRC‐PPU), School of Life Sciences University of Dundee Dundee UK; ^2^ Department of Biology Stanford University Stanford CA USA; ^3^ Centre for Gene Regulation and Expression, School of Life Sciences University of Dundee Dundee UK

**Keywords:** E3 ligase, enzyme substrate, post‐translational modification, ribosome, ubiquitin‐like modifier, Post-translational Modifications & Proteolysis

## Abstract

Protein UFMylation, i.e., post‐translational modification with ubiquitin‐fold modifier 1 (UFM1), is essential for cellular and endoplasmic reticulum homeostasis. Despite its biological importance, we have a poor understanding of how UFM1 is conjugated onto substrates. Here, we use a rebuilding approach to define the minimal requirements of protein UFMylation. We find that the reported cognate E3 ligase UFL1 is inactive on its own and instead requires the adaptor protein UFBP1 to form an active E3 ligase complex. Structure predictions suggest the UFL1/UFBP1 complex to be made up of winged helix (WH) domain repeats. We show that UFL1/UFBP1 utilizes a scaffold‐type E3 ligase mechanism that activates the UFM1‐conjugating E2 enzyme, UFC1, for aminolysis. Further, we characterize a second adaptor protein CDK5RAP3 that binds to and forms an integral part of the ligase complex. Unexpectedly, we find that CDK5RAP3 inhibits UFL1/UFBP1 ligase activity *in vitro*. Results from reconstituting ribosome UFMylation suggest that CDK5RAP3 functions as a substrate adaptor that directs UFMylation to the ribosomal protein RPL26. In summary, our reconstitution approach reveals the biochemical basis of UFMylation and regulatory principles of this atypical E3 ligase complex.

## Graphical abstract

Reconstitution of UFM1 conjugation shows that the UFL1/UFBP1 E3 complex activates the E2 UFC1 for aminolysis and that ribosome monoUFMylation is promoted by the CDK5RAP3 adaptor.

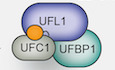

## Introduction

UFM1 is a ubiquitin‐like (Ubl) modifier that contains the characteristic β‐grasp fold found in all UBLs and is highly conserved in eukaryotes (Sasakawa *et al*, 2006; Van Der Veen & Ploegh, 2012). Like other UBLs, UFM1 is covalently attached to lysine residues on substrates via an enzymatic cascade involving an E1 activating enzyme, UFM1‐activating enzyme 5 (UBA5), E2 conjugating enzyme, UFM1‐conjugating enzyme 1 (UFC1), and an E3 ligase, UFM1 E3 ligase 1 (UFL1; Komatsu *et al*, 2004; Cappadocia & Lima, 2018). Recent studies have identified that UFMylation of the ribosomal protein RPL26 plays a critical role in endoplasmic reticulum (ER) homeostasis (Walczak *et al*, 2019; Liang *et al*, 2020; Wang *et al*, 2020). Besides ER‐associated roles, UFMylation has also been implicated in other cellular processes including protein translation, DNA damage response, nuclear receptor‐mediated transcription and development (Yoo *et al*, 2014; Lee *et al*, 2019; Qin *et al*, 2019; Wang *et al*, 2019; preprint: Gak *et al*, 2020; Liu *et al*, 2020). Further, loss of or mutations of components of the UFMylation machinery has been linked to many diseases such as cancer, type‐2 diabetes, neurological disorders, and cerebellar ataxia, where the failure of ER homeostasis and protein quality control could be one of the major contributing factors (Yoo *et al*, 2014; Duan *et al*, 2016; Liu *et al*, 2017; Nahorski *et al*, 2018; Xie *et al*, 2019). Indeed, UFMylation is an essential post‐translational modification for animal development as loss of UFM1 or any of the UFMylation enzymes results in the failure of erythropoiesis and embryonic lethality (Lemaire *et al*, 2011; Tatsumi *et al*, 2011; Cai *et al*, 2015, 2016).

UFM1 is synthesized in a precursor form consisting of 85 amino acids that are then post‐translationally processed by UFM1‐specific proteases (UFSPs) to remove the last two residues at the C‐terminus generating mature UFM1 that contains an exposed C‐terminal glycine, Gly^83^ (Sung *et al*, 2007). Mature UFM1 is activated by the E1, UBA5 via the formation of a high energy thioester bond between Cys^250^ of UBA5 and Gly^83^ of UFM1 (Komatsu *et al*, 2004; Oweis *et al*, 2016). Activated UFM1 is then transferred from UBA5 onto the catalytic Cys^116^ of UFC1 (Komatsu *et al*, 2004; Banerjee *et al*, 2020), which has a canonical UBC fold and an N‐terminal helical extension whose function is not known (Mizushima *et al*, 2007; Liu *et al*, 2009). UFC1 lacks many of the features conserved in many E2s such as the catalytic HPN motif suggesting unique modes of action and regulation. Further, some ubiquitin (Ub) E2 enzymes associate with Ub via a backside interaction, which mediates E2 dimerization, helping stabilize E2s in their active‐closed state and enhancing their catalytic efficiency (Brzovic *et al*, 2006; Buetow *et al*, 2015; Stewart *et al*, 2016; Patel *et al*, 2019). For UFC1, it is unknown whether similar mechanisms exist to regulate its activity as detailed biochemical characterization is lacking.

Transfer of UFM1 from UFC1 to substrates is thought to be mediated by UFL1, the only E3 ligase identified in the UFMylation pathway to date (Tatsumi *et al*, 2010). Deletion of UFL1 results in loss of UFMylation and mice lacking Ufl1 exhibit a failure in hematopoiesis and embryonic lethality suggesting that it could be the main or sole E3 ligase (Cai *et al*, 2019). Although the role of UFL1 in UFMylation is well established, the mechanism of how it functions as an E3 ligase is not understood (Banerjee *et al*, 2020). In general, E3 ligases are classified either as scaffold‐type ligases that bring together a UBL‐charged E2 with a substrate for direct transfer of the UBL from the E2 to the substrate, or as Cys‐dependent enzymes where the UBL is first transferred from the E2 to the catalytic Cys of the E3 for subsequent transfer to the substrate (Deol *et al*, 2019). Examples of scaffold‐type E3s are RING family ligases and RANBP2 whereas HECT, RBR, and RCR enzymes typify the latter type (Deshaies & Joazeiro, 2009; Pichler *et al*, 2017; Lorenz, 2018; Pao *et al*, 2018; Walden & Rittinger, 2018). Intriguingly, UFL1 does not possess any conserved sequence or domain features found in known E3 ligases. Hence it is not clear whether UFL1 is a Cys‐dependent E3 enzyme or uses a scaffolding mechanism.

UFL1 is predominantly found anchored at the ER through its interaction with an adaptor protein, DDRGK1/UFBP1 (UFM1 binding protein‐1), which has been suggested to be one of its substrates (Tatsumi *et al*, 2010; Wu *et al*, 2010). UFBP1 localizes to the ER membrane via an N‐terminal transmembrane segment (Walczak *et al*, 2019). Intriguingly, loss of UFBP1 affects the stability and expression levels of UFL1 (Tatsumi *et al*, 2010; Wu *et al*, 2010; Cai *et al*, 2015). CDK5RAP3 is another poorly characterized protein commonly associated with UFL1. Several reports have led to speculations that UFL1, UFBP1, and CDK5RAP3 together form an E3 ligase complex (Wu *et al*, 2010; Banerjee *et al*, 2020; Witting & Mulder, 2021). Only a handful of substrates of UFL1 have been identified to date and most of them are ER‐localized (Gerakis *et al*, 2019; Witting & Mulder, 2021). In addition to monoUFMylation, polyUFM1 chains can be assembled, which are mainly linked via K69 (Yoo *et al*, 2014). In the Ub system, the type of polyUb linkage formed is generally dictated by the last enzyme in the cascade that forms a thioester linkage with Ub before transfer to the substrate. Scaffold‐type E3s such as RING E3 ligases bind to charged E2 (E2~Ub) to mediate the transfer of Ub onto the substrate, and hence, the E2 enzymes are thought to determine the linkage type assembled. By contrast, catalytic Cys containing E3 ligases form an E3~Ub thioester intermediate and dictate the linkage type formed (Deshaies & Joazeiro, 2009; Deol *et al*, 2019).

Given the lack of understanding of the molecular mechanism underpinning the UFM1 conjugation machinery and the mechanisms governing its regulation, here we adopt a rebuilding approach with purified components to reconstitute UFMylation *in vitro*. Using this approach, we reveal that UFL1 on its own is an unstable protein that cannot support UFMylation. We demonstrate that UFL1 together with UFBP1 forms a functional heterodimeric E3 ligase complex and classify it as a scaffold‐type E3 ligase. Further, we identify CDK5RAP3 to bind to the E3 ligase complex and inhibit UFMylation by preventing the discharge of UFM1 from UFC1. Importantly, by reconstituting UFMylation of ribosomes *in vitro*, we find that CDK5RAP3 has a regulatory function to restrict UFMylation to *bona fide* substrates. Our mechanistic insights describe an atypical E3 ligase complex and provide a foundation for investigating its catalytic mechanism, regulation, and substrate specificity.

## Results

### UFL1 together with UFBP1 forms an active E3 ligase complex

**Figure 1:**
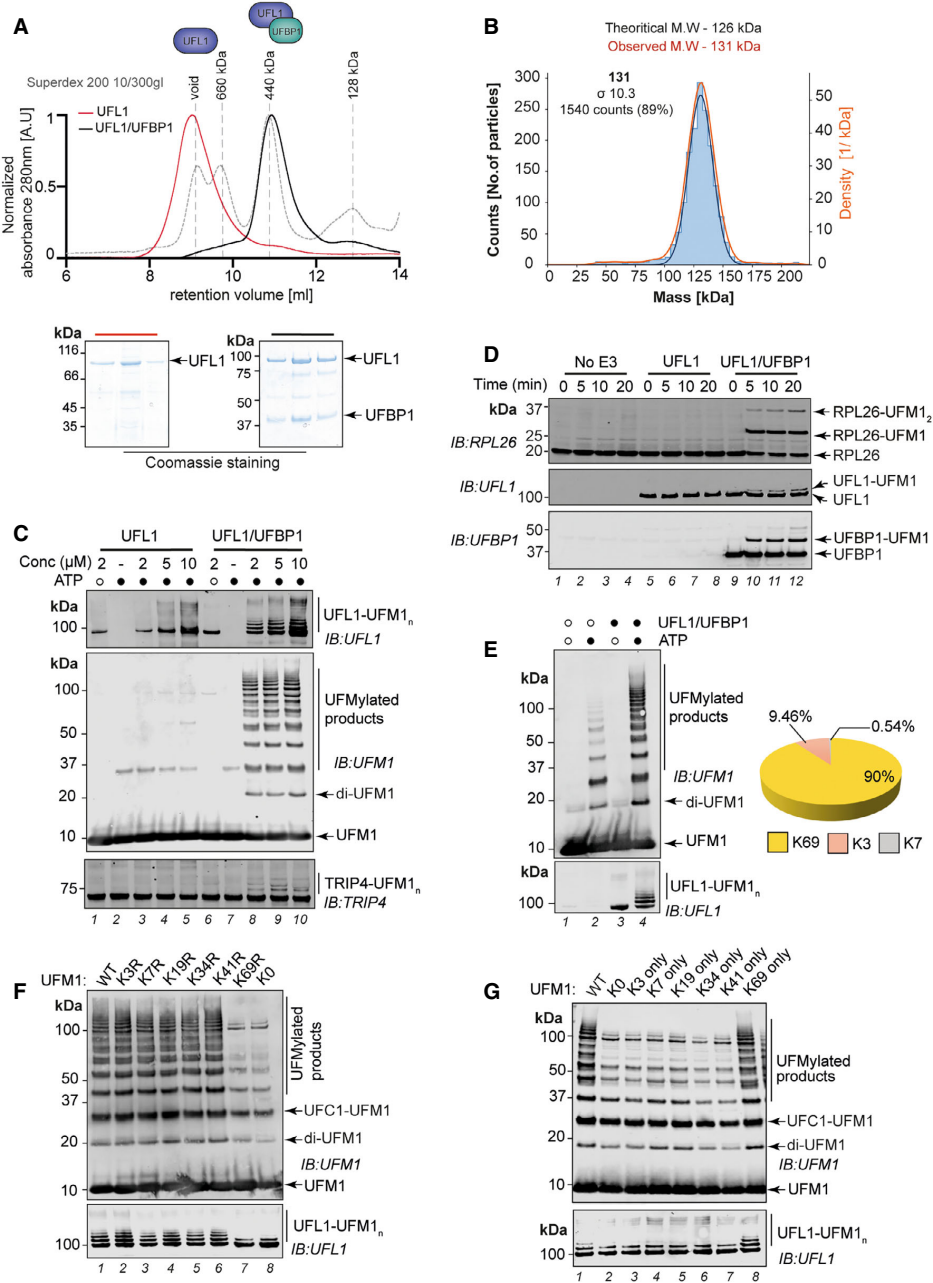
*In vitro* reconstitution of active UFM1 E3 ligase AComparison of size exclusion chromatography profiles of UFL1 expressed alone (Red) and co‐expressed UFL1/UFBP1 complex (Black) run under identical buffer conditions on a Superdex™ 200 Increase 10/300 GL column. Molecular weight standards are shown in gray. Fractions corresponding to each peak were collected and analyzed on 4–12% denaturing SDS–PAGE followed by Coomassie staining, which is shown below.BMass photometry analysis of co‐purified UFL1/UFBP1 complex. The theoretical and experimental molecular weights are indicated above.CImmunoblot comparing UFL1 autoUFMylation (*Top*) and UFM1 chain synthesis (*Middle*) in the presence of UFL1 expressed alone and in complex with UFBP1. (*Bottom*) Assays to monitor UFMylation of substrates using purified TRIP4. Reaction products were separated on a 4–12% SDS–PAGE gel under reducing conditions and analyzed by immunoblotting using indicated antibodies.D
*In vitro* reconstitution of 60S ribosome UFMylation. Purified 60S ribosome was incubated with either UFL1 on its own or UFL1/UFBP1 complex together with UBA5, UFC1, and UFM1 for indicated time points at 30°C.EImmunoblot showing the formation of free UFM1 chains in the presence (lane 3 and 4) and absence of UFL1/UFBP1 (lane 1 and 2). (*Bottom*) Graphical representation of the linkage composition of di‐UFM1 chains obtained from LC–MS/MS analysis.FImmunoblot analyzing polyUFMylation (*top*) and UFL1 autoUFMylation (*bottom*) in the presence of indicated Lys to Arg (K‐R) and Lys‐less (K0) mutants of UFM1.GImmunoblot showing *in vitro* UFMylation assay to check for the formation of di‐UFM1 chains in the presence of WT, Lys‐less (K0), and single lys mutants (K only) of UFM1. Data information: Data shown in (C–G) are representative of at least three independent experiments. Source data are available online for this figure.

**Figure EV1:**
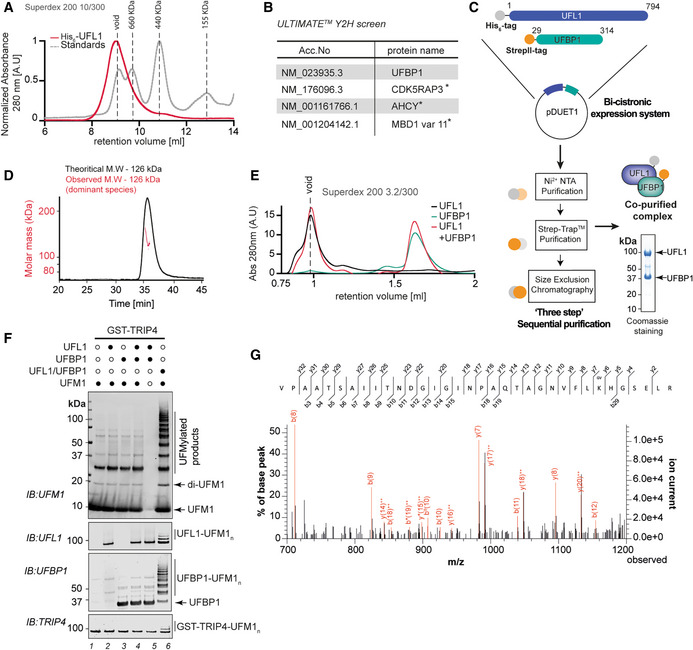
*In vitro* reconstitution of the active UFM1 E3 ligase ASize exclusion chromatography profile of UFL1 run on Superdex™ 200 Increase 10/300 GL column (shown in red). Overlay of UV chromatogram of molecular weight standards of different sizes (shown in grey) run under same buffer conditions.BResults obtained from ULTIMATE Y2H™ screening (Hybrigenics) for binary interactions with UFL1. Asterisk (*) denotes proteins hits obtained with low confidence.CSchematic describing the strategy for co‐expression and purification of UFL1/UFBP1 complex.DSEC‐MALS analysis of UFL1/UFBP1 complex. The theoretical and observed molecular weights are indicated above.EAnalytical gel filtration chromatography analysis showing UV traces of UFL1 and UFBP1 run on their own and a mixture containing UFL1 and UFBP1. Superdex™ 200 Increase 3.2/300 column was used for analysis.FUFBP1 does not activate UFL1 when added exogenously. *In vitro* UFMylation assays to compare the E3 ligase activity of UFL1 and UFBP1 expressed alone and together as a complex. 0.25 μM UBA5, 5 μM UFC1 and 10 μM UFM1 was incubated with 1 μM UFL1 or 1 μM UFBP1 or 1 μM UFL1/UFBP1 complex for 1 h at 37°C in the buffer containing 50 mM HEPES 7.5, 0.5 mM DTT, 10 mM MgCl_2_ and 10 mM ATP. The reaction was stopped by addition of 3× SDS loading dye and run on a 4–12% denaturing SDS PAGE gel under reducing conditions and immunoblotting was performed using indicated antibodies.GMS^2^ spectra showing the peptide derived from *in vitro* UFMylation assay showing the VG‐remnant on K69 of UFM1. Source data are available online for this figure.

The interaction of UFC1 with UBA5 and how UFM1 is transferred from UBA5 to the catalytic Cys are well understood (Oweis *et al*, 2016; Padala *et al*, 2017; Soudah *et al*, 2019; Banerjee *et al*, 2020; Kumar *et al*, 2021). We therefore focused on subsequent events in the UFMylation cascade. To analyze how the E2 UFC1 works together with the E3 ligase UFL1 to transfer UFM1, we aimed to reconstitute UFMylation reactions using purified components. We expressed 6xHis‐UFL1 in *Escherichia coli* and purified it by affinity chromatography as the first step. Subsequent size exclusion chromatography (SEC) analysis indicated UFL1 to form soluble aggregates as it was found to elute in the void fraction (Fig EV1A). Our attempts to prevent aggregation using alternative buffers and additives or by incorporating different solubility‐enhancing tags were unsuccessful. As expressing subunits of protein complexes individually may result in aggregation (Warner, 1977; Kihm *et al*, 2002; Yanagitani *et al*, 2017), we reasoned that UFL1 might require additional binding partners for its stability and activity. To identify binding partners of UFL1, we performed yeast two‐hybrid (Y2H) screening using a cDNA library derived from the human placenta as prey, which revealed UFBP1 and CDK5RAP3 as interactors (Fig EV1B). We therefore wondered if co‐expressing UFBP1 with UFL1 might confer solubility and stability to UFL1. To achieve this, we used a co‐expression system to express full‐length UFL1 together with UFBP1 lacking its transmembrane sequence and purified the ligase complex with three purification steps (Fig EV1C). Indeed, UFL1 co‐expressed with UFBP1 (UFL1/UFBP1) is well behaved and no longer formed soluble aggregates in SEC (Fig 1A). Mass photometry (Sonn‐Segev *et al*, 2020) and SEC‐MALS analyses (Figs 1B and EV1D) revealed a stable heterodimeric UFL1/UFBP1 complex. Importantly, the mass photometry measurements are performed at low nanomolar concentrations highlighting the stable nature of the complex.

Having obtained soluble UFL1/UFBP1 complexes, we next tested the catalytic activity of UFL1 expressed alone and in complex with UFBP1 using *in vitro* UFMylation assays containing UBA5, UFC1, and UFM1 and ATP. Whereas UFL1 on its own did not show any appreciable activity, the UFL1/UFBP1 complex is an active ligase as evidenced by UFL1 autoUFMylation and the formation of multiple UFMylated products that correspond to free UFM1 chains and UFMylated UFBP1 and UFC1 (Fig 1C). To check whether UFL1/UFBP1 was also capable of UFMylating substrates *in vitro*, we used TRIP4/ASC1, a protein reported to be UFMylated in cells (Yoo *et al*, 2014). Indeed, UFL1/UFBP1 can UFMylate TRIP4 *in vitro* (Fig 1C, *Bottom panel*). Intriguingly, the exogenous addition of purified UFBP1 to UFL1 is not sufficient to restore its E3 ligase activity (Fig EV1E and F). The ribosomal subunit RPL26 is one of the best described UFMylation substrates to date with proteomic analyses identifying RPL26 to be the main UFMylated protein in cells (Walczak *et al*, 2019; Wang *et al*, 2020). Hence, we set up a cell‐free reconstitution of ribosome UFMylation. Strikingly, the addition of UFL1/UFBP1 to purified 60S ribosomes leads to robust modification of RPL26 (Fig 1D), and in line with previous observations, both mono‐ and di‐UFMylated RPL26 are observed. Taken together, these results show that UFL1 and UFBP1 form an obligate heterodimer required for E3 ligase activity.

Since polyUFMylated products are formed in UFMylation reactions, we next analyzed the reaction products by mass spectrometry to determine the linkage types assembled. Interestingly, LC–MS analyses revealed that the polyUFM1 chains formed are predominantly linked via K69 with some K3 and K7 linkages also observed (Figs 1E and EV1G). To validate these observations, we generated a series of K‐R mutants where every Lys in UFM1 was individually mutated to Arg. In line with the mass spectrometry analyses, only UFM1 K69R resulted in near complete loss of polyUFMylated species and unanchored diUFM1, closely resembling reactions with UFM1 K0 in which all Lys were mutated to Arg (Fig 1F, lanes 7 and 8). With UFM1 K69R, mono but not polyUFMylation of UFL1 was observed confirming that UFL1 autoUFMylation involves the formation of K69‐linked polyUFM1 chains (Fig 1F). We next generated K_only_ UFM1 mutants where all but one Lys is mutated to Arg. All the K_only_ mutants along with UFM1 K0 mutant abrogate polyUFMylation formation with the sole exception of K69‐only UFM1 (Fig 1G). In summary, our reconstitution experiments reveal that UFL1 together with UFBP1 forms an active E3 ligase complex that can efficiently UFMylate substrates and assemble polyUFM1 chains that are K69‐linked.

### UFL1/UFBP1 is a scaffold‐type E3 ligase complex activating UFC1 for aminolysis

**Figure 2:**
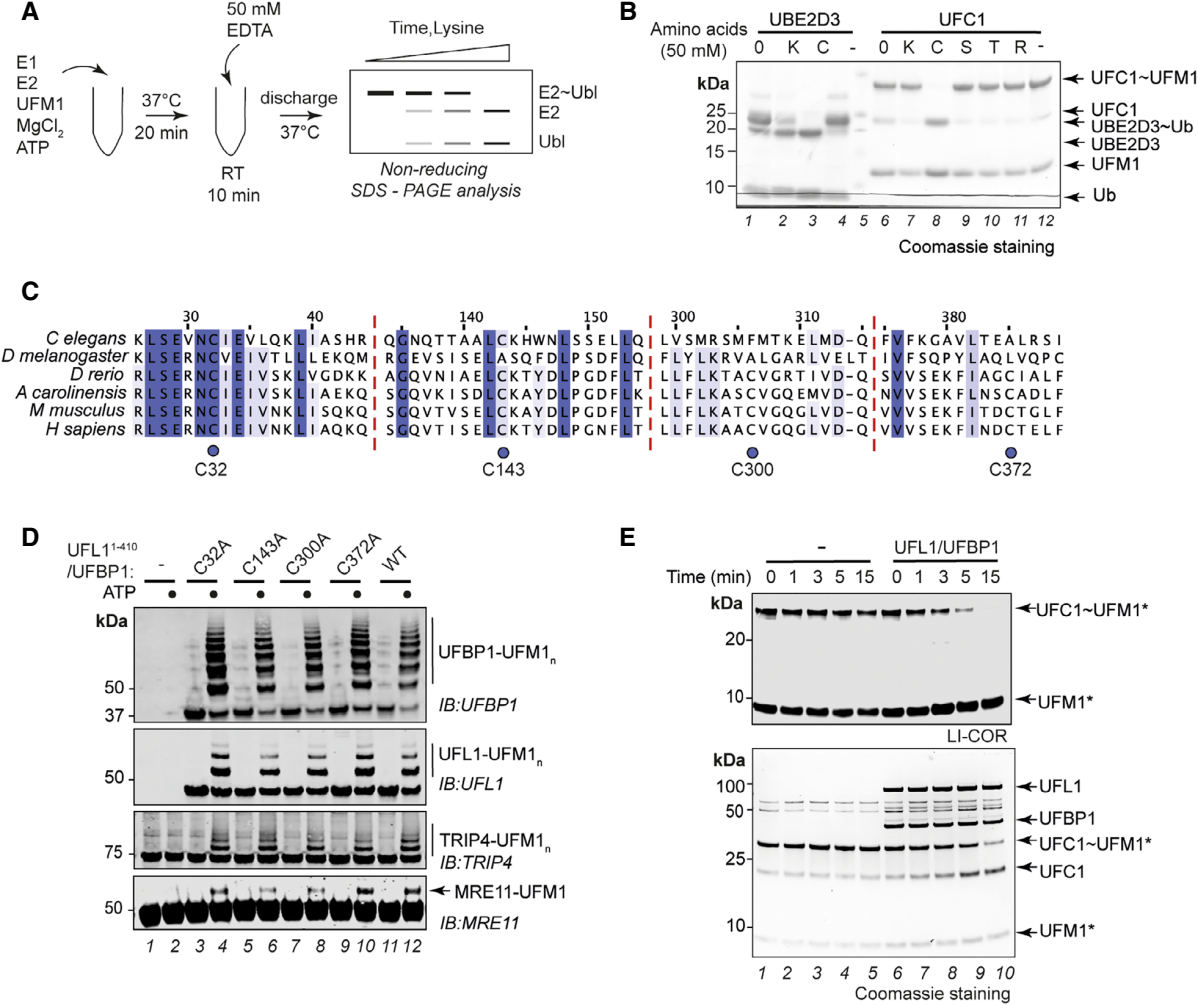
UFL1/UFBP1 complex activates UFC1 for aminolysis ASchematic showing the workflow for substrate‐independent single turnover lysine discharge assays.BCoomassie‐stained gels monitoring the discharge of UFM1 from UFC1 in the presence of indicated free amino acids. UBE2D3 is used as a positive control for lysine and cysteine discharge.CMultiple sequence alignment of 1–410 a.a. region of UFL1 from various organisms to highlight the degree of conservation of cysteine residues in this region.DImmunoblot showing *in vitro* UFMylation assays in the presence of Cys to Ala (indicated as C‐A) mutants of UFL1 to check for UFBP1 UFMylation (*top*), UFL1 autoUFMylation (*middle*), and substrate UFMylation (*bottom*).ESingle turnover lysine discharge assays in the presence and absence of full‐length UFL1/UFBP1 complex. The reaction was stopped by the addition of nonreducing SDS loading buffer (1× final) and analyzed for discharge on a 4–12% SDS–PAGE gel under nonreducing conditions. Top gel—LI‐COR scan of fluorescently labeled UFM1 (UFM1*); bottom gel—Coomassie stained. Data information: Data in (B), (D), and (E) are representative of at least three independent experiments. Source data are available online for this figure.

**Figure EV2:**
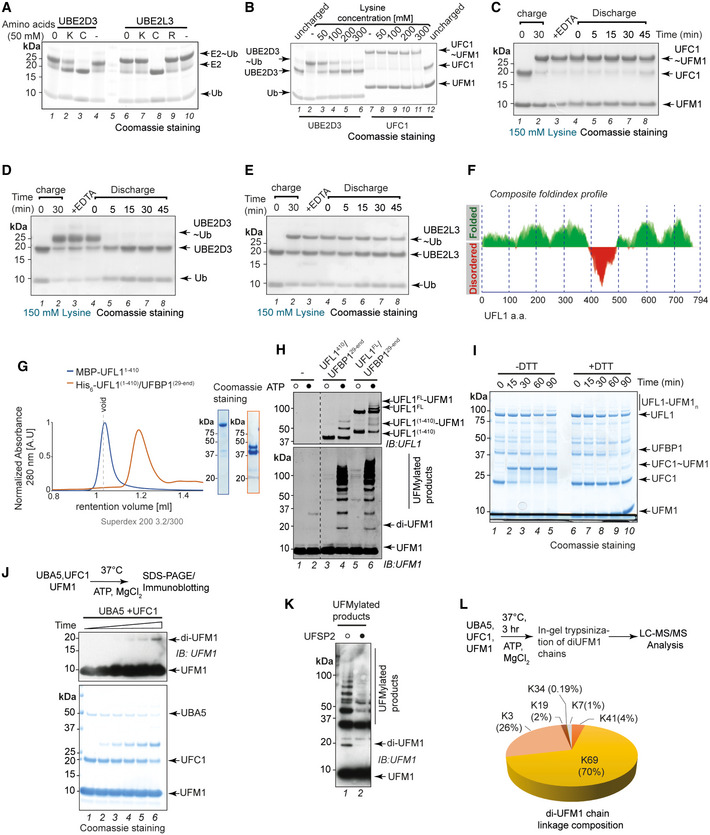
UFL1/UFBP1 is a scaffold‐type E3 ligase ASingle turnover assay to monitor discharge of Ubiquitin from UBE2D3 and UBE2L3 in the presence of free amino acids. “0” indicates time 0.BSingle turnover lysine discharge assays using UBE2D3 and UFC1 in the presence of increasing concentration of free Lysine.CTime‐dependent analysis of discharge of UFM1 from UFC1 in the presence of high concentration of free lysine (150 mM).D, ETime‐dependent analysis of discharge of Ubiquitin from UBE2D3 and UBE2L3 in the presence of 150 mM Lysine.FComposite Foldindex profile of UFL1 showing folding propensity of different regions of UFL1 to aid in construct design for soluble protein expression.GSize exclusion chromatography profile of MBP‐UFL1^1‐410^ (dark blue) and His_6_‐UFL1^1‐410^/UFBP1^29‐end^ (orange) run on Superdex™ 200 Increase 3.2/300 column. Approximately 20 μg of sample was used for analysis. (Right) Coomassie stained gel showing the purity of the proteins.H
*In vitro* UFMylation assay to check for E3 ligase activity of UFL1^1‐410^/UFBP1^29‐end^. Full length UFL1/UFBP1 complex is used as a positive control.ITime dependent analysis of transthiolation activity of UFL1. Reaction products were analysed on a 4–12% SDS PAGE gel under reducing (lane 1–5) and non‐reducing conditions (lanes 6–10).J
*In vitro* UFMylation assays to check for formation of di‐UFM1 chains by minimal reconstitution using UBA5, UFC1 and UFM1 in the presence of MgCl_2_ and ATP.KImmunoblot to check for the presence of free UFM1 chains with and without treatment of UFSP2. *In vitro* UFMylation products generated as shown in (E) was incubated with UFSP2 (2 μM) for 1 h at 37°C. The reaction was stopped by addition of SDS‐loading buffer (1× final) and run on a 4–12% SDS PAGE gel under reducing conditions followed by immunoblotting using indicated antibodies to check for the disappearance of polyUFMylated products especially di‐UFM1.LGraphical representation showing the composition of linkage forms of di‐UFM1 chains formed by minimal reconstitution of UBA5 and UFC1. Source data are available online for this figure.

Intrinsic reactivity of E2s assayed using free amino acids can give insights into the ability of E2s to transfer UBLs onto substrates and can provide clues about the type of E3 ligase they work with and the nature of the substrate (Stewart *et al*, 2016; Pao *et al*, 2018). For instance, E2s such as UBE2D3, which function together with RING/scaffold‐like E3 ligases are capable of aminolysis and are therefore reactive towards the free amino acid Lys (Figs 2A and EV2A; Wenzel *et al*, 2011). On the other hand, UBE2L3, which functions only with Cys‐dependent E3 ligases is incapable of aminolysis and lacks reactivity to free Lys on its own (Fig EV2A; Wenzel *et al*, 2011). We therefore analyzed the intrinsic reactivity and stability of the E2~UBL, i.e., UFC1~UFM1 (~ denotes the thioester bond between UFM1^G83^ and UFC1^C116^) and compared this to the well‐characterized ubiquitin E2s, UBE2D3, and UBE2L3. When the discharge of UFM1 from UFC1~UFM1 onto free amino acids was analyzed, we observed discharge onto Cys while no discharge was observed in the presence of Lys, Arg, Ser, and Thr (Fig 2B). Under the same experimental conditions, UBE2D3 discharged Ub in the presence of both Cys and Lys (Fig 2B, lanes 2 and 3). Further, using increasing concentrations of free Lys or with prolonged incubation in a time course, we find that UFC1 has negligible Lys reactivity, which is similar to UBE2L3 (Fig EV2B–E).

Since UFC1 is reactive only to cysteines, we systematically assessed potential catalytic Cys residues to determine whether UFL1 is a Cys‐dependent enzyme. The N‐terminal region of UFL1 contains four Cys residues, and sequence analysis shows that all four vary in their degree of conservation with C32 being the most conserved (Fig 2C). Based on analysis of predicted folding propensity (Fig EV2F) and secondary structure prediction, we made a C‐terminal truncation of UFL1, UFL1^1‐410^, which when expressed on its own formed soluble aggregates like full‐length UFL1 and required co‐expression of UFBP1 to obtain a heterodimeric complex (Fig EV2G). Importantly, *in vitro* UFMylation assays showed that UFL1^1‐410^/UFBP1 complex is an active E3 ligase (Fig EV2H). To identify the catalytic Cys in UFL1, we mutated each Cys individually to Ala in the UFL1^1‐410^/UFBP1 complex. To our surprise, *in vitro* UFMylation assays showed that none of the single mutants affected the autoUFMylation activity of UFL1 or UFMylation of substrates, implying that UFL1 does not contain a catalytic Cys (Fig 2D). Further, we do not observe any transthiolation products of UFL1/UFBP1 demonstrating that UFM1 is not transferred to a Cys residue in the E3 ligase (Fig EV2I). Since UFBP1 does not have any Cys residues, these results suggest that the UFL1/UFBP1 ligase complex may instead employ a scaffolding mechanism to transfer UFM1 onto substrates.

The lack of a transthiolation activity raises the possibility that the UFL1/UFBP1 ligase complex could induce aminolysis of UFC1~UFM1. Hence, we compared the discharge of UFM1 from UFC1~UFM1 onto Lys in the absence and presence of the ligase complex. Whereas UFC1 does not discharge UFM1 onto Lys on its own, it was able to do so in the presence of the UFL1/UFBP1 E3 complex (Fig 2E). Based on these observations, we suggest that UFL1/UFBP1 functions as a scaffold‐type E3 ligase that binds to charged UFC1 to promote aminolysis.

While inefficient, UFC1 on its own can assemble free UFM1 chains, an activity that is significantly enhanced in the presence of the UFL1/UFBP1 ligase complex (Fig EV2J and K). Interestingly, UFC1 assembles mainly K69 linkages (Fig EV2L) and this linkage specificity is maintained in the presence of UFL1/UFBP1 (Fig 1E). Thus, linkage specificity is determined by the E2 and is not altered by the E3 ligase complex, a feature commonly observed in ubiquitin RING E3 ligases (Deng *et al*, 2000; Deshaies & Joazeiro, 2009; Branigan *et al*, 2015). These results further strengthen our conclusion that UFL1/UFBP1 is a scaffold‐type ligase complex.

### Tandem WH domains of UFL1/UFBP1 constitute minimal E3 ligase

**Figure 3:**
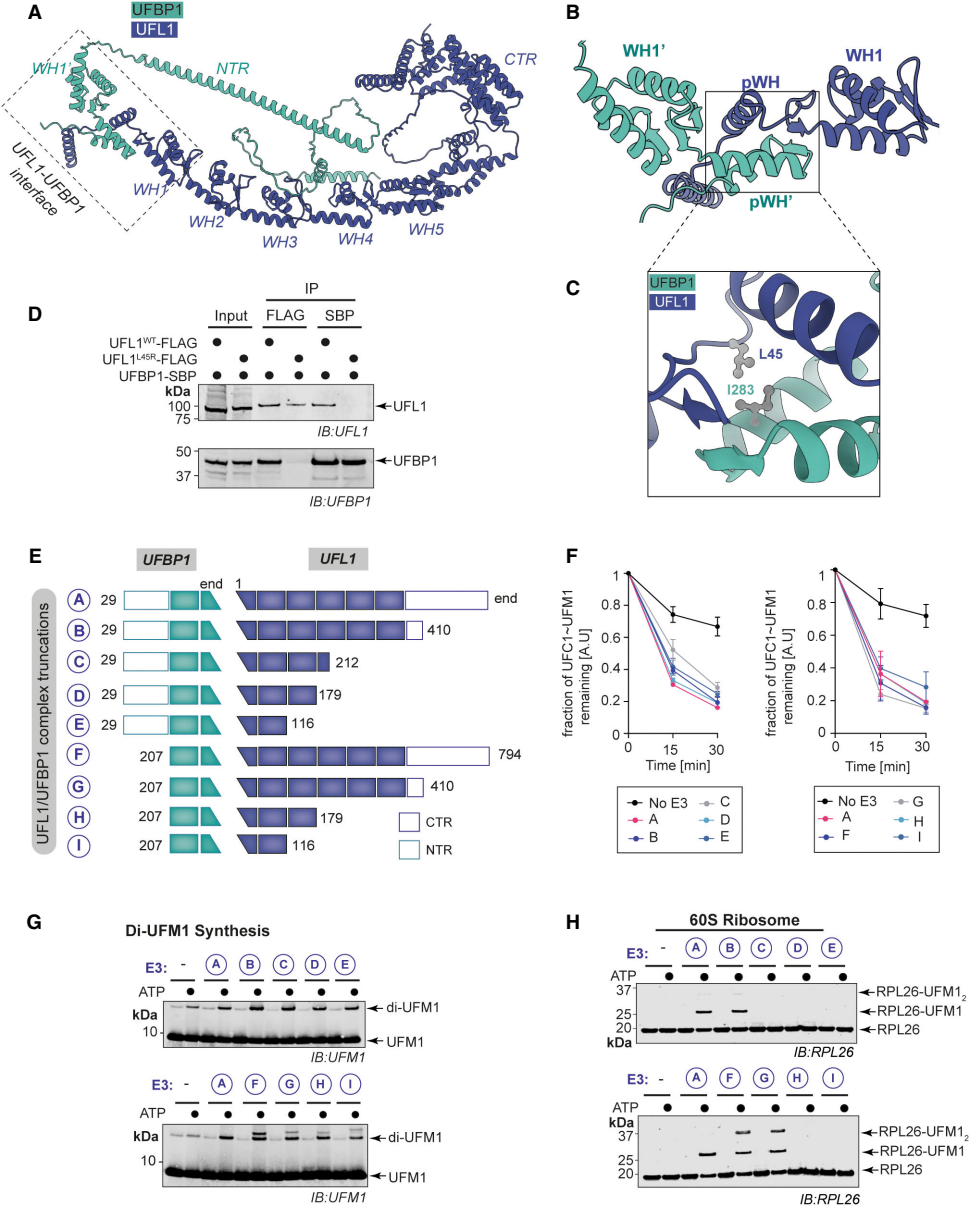
Biochemical characterization of UFL1/UFBP1 complex ACartoon representation of predicted full‐length human UFL1/UFBP1 complex using AlphaFfold. UFL1 and UFBP1 are shown in blue and teal colors, respectively. The dimeric interface of UFL1 and UFBP1 is highlighted in the dotted box. WH, Winged helix; pWH, Partial Winged helix; NTR, N‐terminal region; CTR, C‐terminal region.BView of the dimeric interface of UFL1/UFBP1 complex highlighting the formation of a full composite WH domain by two partial winged helix domains of UFL1 (blue) and UFBP1 (teal).CClose‐up view of the interface of UFL1/UFBP1 complex highlighting the interactions at the pWH‐pWH′ domain interface centered on UFL1 Leu45 (L45) and UFBP1 Ile283 (I283).DImmunoblot from immunoprecipitation (IP) assays to validate the predicted model of UFL1/UFBP1 interaction in cells. UFL1^WT^‐3xFLAG and UFL1^L45R^‐3xFLAG were transiently overexpressed along with UFBP1‐SBP in HEK293T‐UFL1 KO cells and subjected to separate pulldowns using anti‐FLAG antibody or streptavidin. Immunoprecipitated material was run on a 4–12% SDS–PAGE gel and analyzed by immunoblotting using indicated antibodies.ESchematic representation of UFL1/UFBP1 constructs with different domain boundaries designed to identify the minimal catalytic region of the UFL1/UFBP1 complex required for aminolysis.FQuantitative representation of single turnover lysine discharge assays to identify the minimal boundaries of UFL1 (left) and UFBP1 (right) required for activation of UFC1 (*n* = 3, mean ± SD). A representative gel image used for the quantitative analysis is shown in Fig EV4A and B.GImmunoblot showing *in vitro* UFMylation assay to monitor the formation of free di‐UFM1 chains in the presence of UFL1/UFBP1 complexes bearing different domain boundaries.HImmunoblot of *in vitro* assay monitoring UFMylation of 60S Ribosome in the presence of different UFL1/UFBP1 truncations as depicted in (E). Data information: Data in (F–H) are representative of at least three independent experiments. Source data are available online for this figure.

**Figure EV3:**
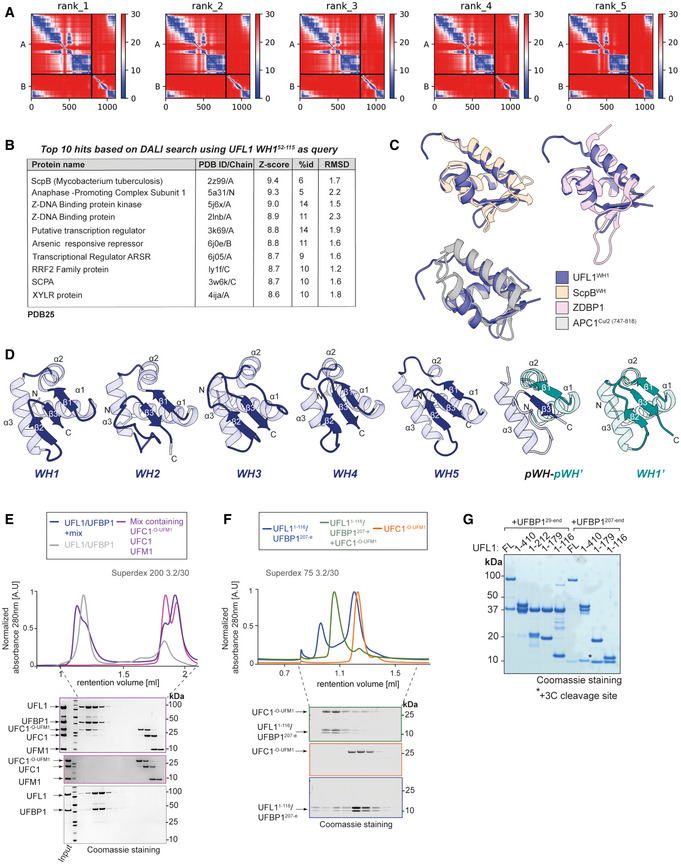
Identifying the minimal ligase domain of UFL1/UFBP1 complex APredicted Aligned Error (PAE) scores of UFL1/UFBP1 models predicted using AlphaFold.BList of structurally similar proteins from the PDB25 database predicted using UFL1 WH1 (52–115 a.a) domain as query structure using DALI server.COverlaid structures of Winged helix 1 (WH1) domain of UFL1 (52–115 a.a) and top three hits obtained from DALI search shown in cartoon representation.DComparison of winged helix domains of UFL1 (shown in blue) and UFBP1 (shown in teal) to highlight their structural similarities.ESEC elution profiles showing that full length UFL1/UFBP1 preferentialy interacts with charged E2. UFL1/UFBP1 complex was incubated with UFC1^‐O‐UFM1^ at a 1:1 molar ratio for 20 min at 4°C and loaded on a Superdex™ 200 Increase 3.2/300 column. The fractions corresponding to each peak were collected and separated on a 4–12% SDS PAGE gel followed by Coomassie staining.FMinimal catalytic region is sufficient for interaction with charged UFC1. UFL1^(1‐179)^/UFBP1^(1‐116)^ complex was incubated with UFC1^‐O‐UFM1^ at the molar ratio of 1:1 for 20 min at 4°C and analysed by analytical size exclusion chromatography as described in (E).GCoomassie stained gel showing purity of different UFL1/UFBP1 truncations. Source data are available online for this figure.

As UFL1/UFBP1 does not have any obvious sequence or domain features found in any of the known E3 ligases, we attempted to define the minimal catalytic region. As our efforts to experimentally determine the structure of this complex were not successful, we used AlphaFold to predict the structure (Fig 3A; Jumper *et al*, 2021; Mirdita *et al*, 2022). Structure prediction of the complex had good predicted aligned error (PAE) scores (Fig EV3A) and shows UFL1 and UFBP1 to form a heterodimer, which are composed of several winged helix (WH) domain repeats (Figs 3A and EV3B and C). These WH domains could be classified as PCI‐like WH domains, named so because they commonly found in components of the proteasome lid, the COP9 signalosome, and the eukaryotic translation initiator, eIF3 (Scheel & Hofmann, 2005; Stewart, 2012). Overall, UFL1 has an N‐terminal helix (a.a 1–25) followed by a partial WH (pWH) domain and five WH domains that extend into a stack of α‐helices at its C‐terminus which we refer to as CTR (C‐terminal region). Likewise, UFBP1 has an N‐terminal transmembrane segment, a long helical region which we refer to as NTR (N‐terminal region) followed by a WH (WH1′) and a partial WH (pWH′) domain. Surprisingly, the partial pWH at the N‐terminus of UFL1 complements the partial pWH′ at the C‐terminus of UFBP1 to form a composite WH (pWH‐pWH′) domain (Fig 3B). The predicted formation of this composite WH domain provides a potential explanation to why UFBP1 is required for the stability of UFL1.

The predicted seven WH repeats of the UFL1/UFBP1 complex are arranged such that α‐helix 1 from each WH domain creates a helical backbone. All predicted WH domains of UFL1 and UFBP1 have identical folds except for a β‐strand, which is missing in the composite WH domain, WH1 domain, and WH2 domain (Fig EV3D). The predicted helical nature of UFBP1 and the helical backbone of UFL1 may explain the unusual migration of UFL1/UFBP1 by SEC. To test the predicted structure of the UFL1/UFBP1 complex, we mutated a key residue at the pWH‐pWH′ interface (UFL1 L45R) to disrupt complex formation (Fig 3C). As expected, co‐immunoprecipitation experiments in HEK293 cells expressing tagged versions of UFL1 and UFBP1 revealed that the UFL1 L45R mutant is unable to form a complex with UFBP1 (Fig 3D). Further support to this model is provided by our Y2H screen, which identifies the region spanning a.a 268–298, i.e., the C‐terminal portion of UFBP1 to interact with UFL1. Hence, we conclude that the formation of the composite WH (pWH‐pWH′) domain is essential for complex formation and protein stability.

**Figure EV4:**
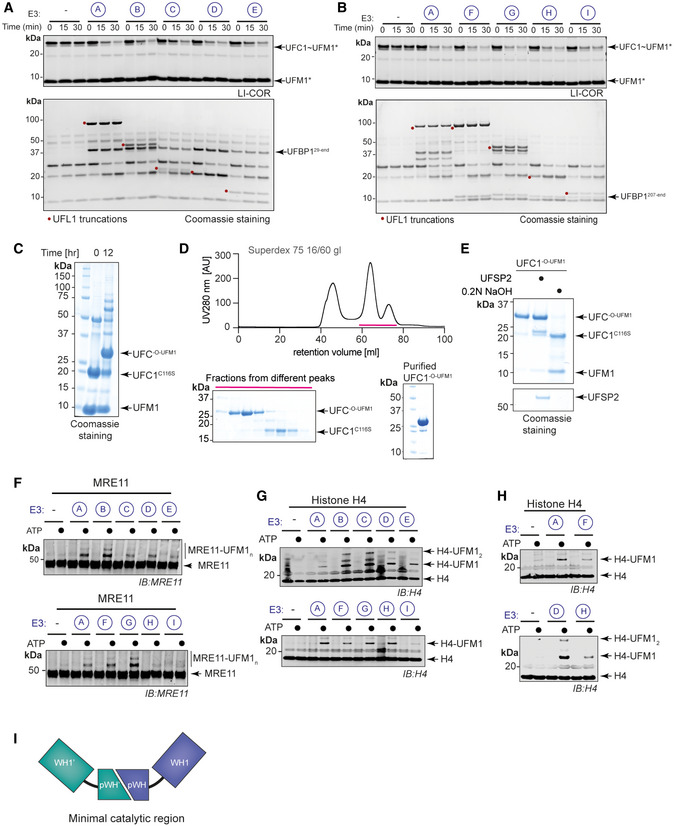
Identifying the minimal ligase domain of UFL1/UFBP1 complex ALysine discharge assays to check for activation of UFC1 in the presence of different UFL1 truncations. Top gel: LI‐COR scan of fluorescently labelled UFM1 (UFM1*); bottom gel—Coomassie stained (representative of three independent experiments); Related to Fig 3F.BLysine discharge assays as in (A) in the absence of NTR of UFBP1. Top gel: LI‐COR scan of fluorescently labelled UFM1 (UFM1*); bottom gel—Coomassie stained (representative of three independent experiments); Related to Fig 3F.CCoomassie stained gel showing analysis of *in vitro* UFMylation reaction products to check for the formation of stable oxy‐ester linked UFC1‐UFM1 conjugate (UFC1^‐O‐UFM1^).DChromatogram obtained from SEC analysis using HiLoad™ 16/60 Superdex™ 75 pg column. (Bottom left) The fractions collected were run on an SDS‐PAGE gel to identify fractions that contained pure UFC1^‐O‐UFM1^. (Bottom right) Coomassie stained gel showing analysis of purified UFC1^‐O‐UFM1^ product to check for homogeneity.EQuality check to analyse if UFC1‐UFM1 conjugate is linked through an oxy‐ester linkage by alkaline hydrolysis.F, GSubstrate UFMylation assays to check for UFMylation of MRE11 and Histone H4 respectively in the presence of different UFL1/UFBP1 truncations.HRole of UFBP1 in substrate UFMylation. (*Top*) Comparison of E3 ligase activity of UFL1/UFBP1^29‐end^ and UFL1/UFBP1^207‐end^ using Histone H4. (*Bottom*) Comparison of E3 ligase activity of UFL1^1‐179^/UFBP1^29‐end^ and UFL1^1‐179^/UFBP1^207‐end^ using purified Histone H4.ISchematic showing the tandem WH domains which constitute the minimal ligase domain. Source data are available online for this figure.

Guided by the insights from the AlphaFold predictions, we made additional truncations to map the minimal catalytic domain of the ligase complex (Fig 3E). The truncated complexes expressed well and were purified to homogeneity (Fig EV3G). We first analyzed the ability of the different truncated complexes to promote the discharge of UFM1 from UFC1~UFM1 onto Lys. All the truncations tested could discharge UFM1 and the smallest region (denoted as Complex I in Fig 3E) that is able to promote discharge contains the predicted composite WH domain and one WH domain each from UFL1 and UFBP1 (Figs 3F and EV4A and B).

Many E3 ligases bind to E2~UBL conjugates with higher affinity compared with E2 on their own (Metzger *et al*, 2014). To investigate the ability of the different UFL1/UFBP1 complexes to bind to the E2 UFC1, we used analytical SEC. Different UFL1/UFBP1 complexes as shown in Fig 3E were incubated with a mixture containing UFC1, UFM1, and a nonreactive UFC1^‐O‐UFM1^ conjugate where UFM1 is linked to C116S via an oxyester bond (Fig EV4C and D). UFC1^‐O‐UFM1^ is more stable compared to thioester‐linked UFC1~UFM1 and therefore used for these analyses. Post preparation, we incubated UFC1^‐O‐UFM1^ with the deUFMylase UFSP2 or 0.2 N NaOH and observed complete collapse only in alkaline conditions confirming that the purified UFC1^‐O‐UFM1^ is indeed linked via an oxyester linkage (Fig EV4E). Analysis of complex formation by SEC revealed that UFL1/UFBP1 has higher affinity for the UFC1^‐O‐UFM1^ conjugate compared to UFC1 or UFM1 on its own, and the complex is of high enough affinity to elute as a stable complex (Fig EV3E). Interestingly, even the smallest of the UFL1/UFBP1 variants was able to bind to UFC1^‐O‐UFM1^ (Fig EV3F). Taken together, the N‐terminal segment of UFL1 (pWH‐WH1) and the C‐terminal region of UFBP1 (WH1′‐pWH′), which we refer to as UFL1/UFBP1^min^ (denoted as I in Fig 3E) is sufficient for both binding to charged UFC1 and activating UFC1~UFM1 for aminolysis may represent the minimal ligase domain.

Next, we assayed ligase activity by monitoring diUFM1 formation. Like full‐length UFL1, the different C‐terminal UFL1 truncations co‐expressed with UFBP1 did not exhibit any loss of activity (Fig 3G). Thus, the N‐terminal region of UFL1 (pWH‐WH1) is sufficient for its ligase activity. Similarly, truncation of the N‐terminus region (NTR) of UFBP1 did not affect diUFM1 formation. Lastly, we checked the impact of truncations of different regions of the UFL1/UFBP1 complex on their ability to modify 60S ribosomes (Fig 3H). Deletion of the CTR region on UFL1 did not affect UFMylation of 60S ribosome whereas deletion of WH3, WH4, and part of WH2 completely abolished ribosome UFMylation thus underlining the importance of these regions in ribosome UFMylation. Intriguingly, deletion of the NTR of UFBP1 predominantly led to di‐UFMylation of ribosomes suggesting that this region may play a role in substrate recognition and UFMylation. We then expanded this analysis to other reported substrates such as Histone H4 and MRE11 (Fig EV4F and G). While regions important for MRE11 UFMylation were very similar to that of the 60S ribosome, H4 UFMylation was impaired only by simultaneous deletion of NTR of UFBP1 and all WH domains except pWH and WH1. Also, as observed in the case of 60S ribosome, deletion of the UFBP1 NTR significantly impaired Histone H4 UFMylation (Fig EV4H). This suggests that substrate recognition and UFMylation may follow different modes that are dependent on the substrate. In summary, using systematic truncations based on AlphaFold predictions, we here make the surprising discovery that three tandem WH domains (Fig EV4I) of UFL1/UFBP1 are sufficient for E3 ligase activity, but additional regions are required for substrate modification.

### CDK5RAP3 restricts the E3 ligase activity of UFL1/UFBP1

**Figure 4:**
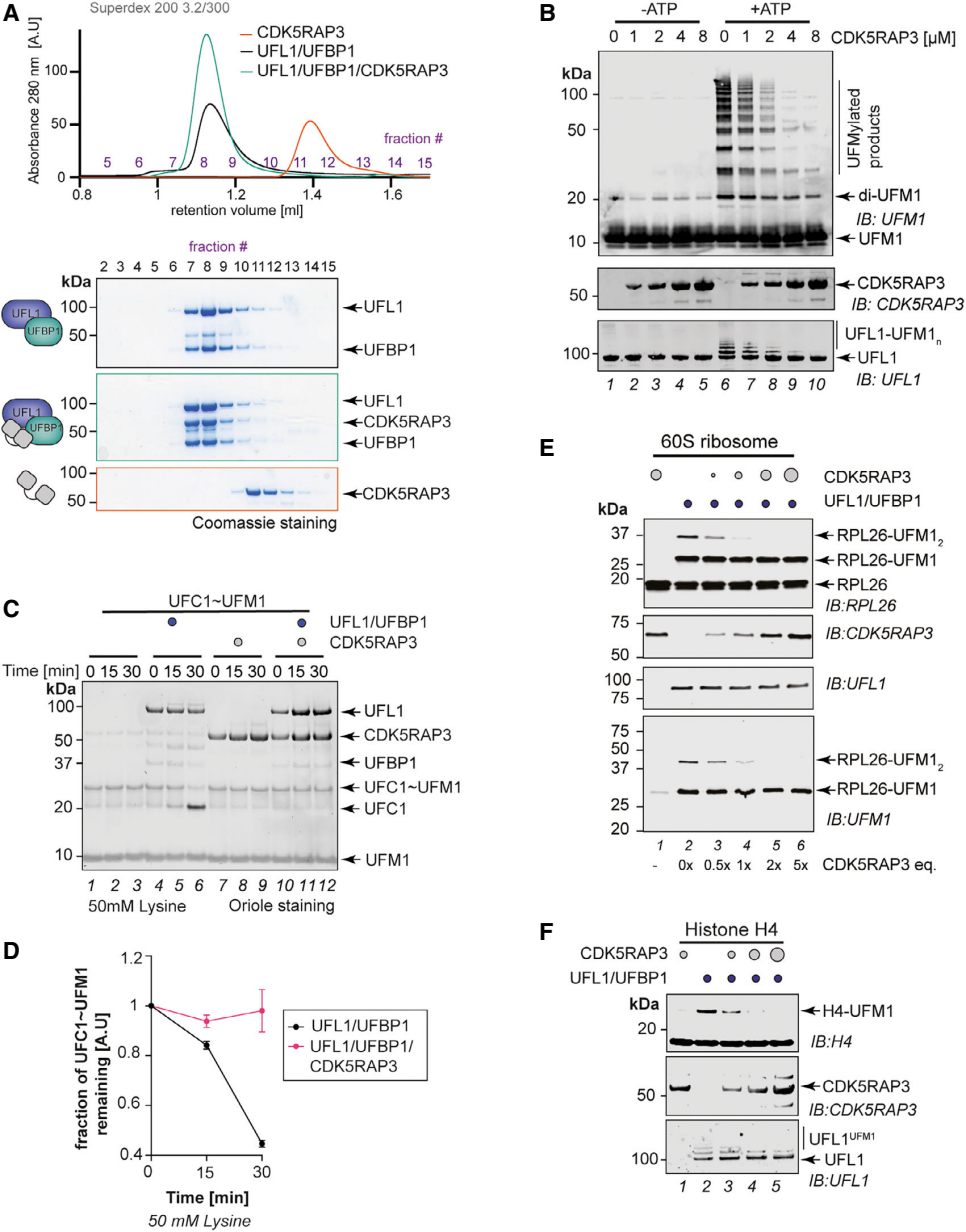
Role of CDK5RAP3 in UFMylation ACDK5RAP3 forms a stable complex with UFL1/UFBP1 *in vitro*. Thirty microliter of UFL1/UFBP1 was mixed with 15 μg of CDK5RAP3 and loaded on a Superdex™ 200 Increase 3.2/300 column analytical gel filtration column. (Bottom) Fractions were collected and analyzed on 4–12% SDS–PAGE under reducing conditions and visualized by Coomassie staining.B
*In vitro* UFMylation assay in the presence of increasing concentrations of CDK5RAP3 to monitor the E3 ligase activity of UFL1/UFBP1 complex. The reaction products were run on a 4–12% SDS–PAGE gel under reducing conditions and immunoblotting was performed using indicated antibodies.CSingle turnover lysine discharge assays to check for activation of UFC1 by UFL1/UFBP1 in the presence and absence of CDK5RAP3. The reaction products were run on a 4–12% SDS–PAGE and visualized by Oriole staining.DQuantification of the discharge of UFM1 from UFC1 in the presence and absence of CDK5RAP3 as seen in (D), *n* = 3 biological replicates, mean ± SD.ESubstrate UFMylation assays using purified 60S Ribosomes in the presence of an increasing concentration of CDK5RAP3. Reaction was performed for 10 min, stopped by the addition of SDS Loading dye, and analyzed on an SDS–PAGE gel under reducing conditions followed by immunoblotting with indicated antibodies.FImmunoblot analysis monitoring UFMylation of Histone H4 in the presence of increasing concentrations of CDK5RAP3 (1, 2, 3 μM). UFL1/UFBP1 concentrations—1 μM. Source data are available online for this figure.

**Figure EV5:**
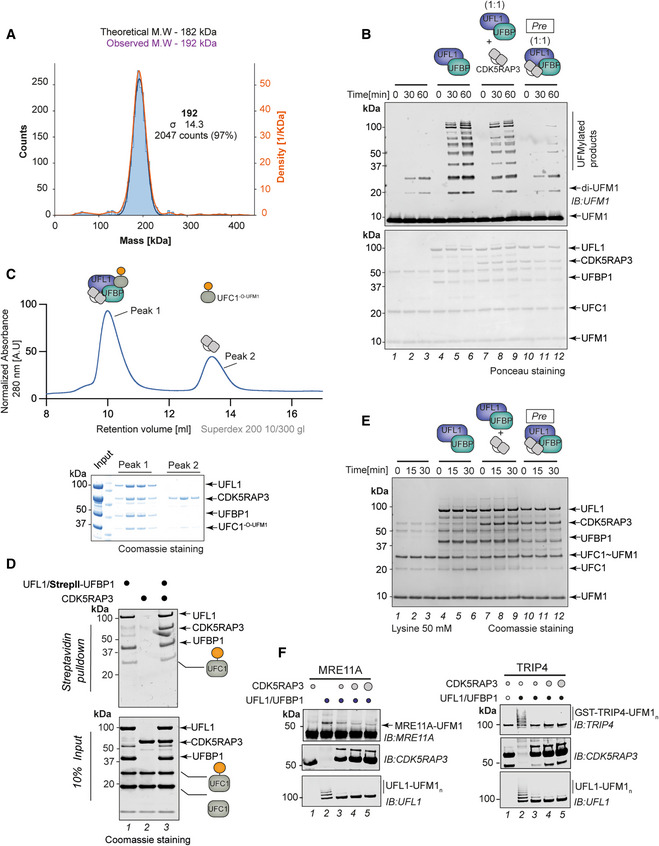
CDK5RAP3 forms a complex with UFL1/UFBP1 and inhibits ligase activity AMass photometry analysis showing the experimental molecular weight of UFL1/UFBP1/CDK5RAP3 complex.B
*In vitro* UFMylation assay to compare the E3 ligase activity of UFL1/UFBP1 mixed with CDK5RAP3 and preassembled ternary E3 ligase complex containing UFL1/UFBP1/CDK5RAP3. Ponceau‐stained nitrocellulose membrane is shown below to indicate the amounts of reaction components used in the assay.CUV traces of gel filtration chromatogram showing co‐migration of (UFL1/UFBP1)/CDK5RAP3/UFC1^‐O‐UFM1^ complex. Approximately, 200 μl of sample containing (UFL1/UFBP1)/CDK5RAP3/UFC1^‐O‐UFM1^ in the molar ratio of 1:1.5:3 was mixed and incubated at 4°C for 1 h and loaded onto a Superdex™ 200 Increase 10/300 GL column. The fractions were collected and analysed on a 4–12% SDS PAGE and visualized by Coomassie staining.DPulldown assay to check for interaction of UFL1/UFBP1 with charged UFC1 in the presence of absence of CDK5RAP3. Around 10 μM of Untagged UFC1 and 10 μM of UFC1‐O‐UFM1 were mixed with 5 μM of UFL1/UFBP1 complex in the presence and absence of CDK5RAP3.ESingle turnover lysine discharge assays to check for UFC1 discharge in the presence of UFL1/UFBP1 mixed with CDK5RAP3 and preassembled UFL1/UFBP1/CDK5RAP3 complex. The reaction products were run on a 4–12% SDS PAGE analysis and visualized by Coomassie staining.F
*In vitro* UFMylation assay to monitor UFMylation of purified substrates namely MRE11A (left) and TRIP4 (right) in the presence of increasing concentration of CDK5RAP3 (1, 2, 3 μM). UFL1/UFBP1 concentration—1 μM. Source data are available online for this figure.

We next analyzed the second hit identified in our Y2H screen, CDK5RAP3, an evolutionarily conserved 53 kDa protein that lacks any known functional domains or motifs. To determine whether CDK5RAP3 can interact with UFL1 in the context of the UFL1/UFBP1 complex, we incubated recombinant full‐length CDK5RAP3 with UFL1/UFBP1 and analyzed complex formation by analytical SEC. Analysis of UV chromatograms and corresponding fractions by SDS PAGE, confirmed that CDK5RAP3 interacts with UFL1/UFBP1 *in vitro* and forms a stable complex (Fig 4A). We further analyzed the complex by mass photometry, which confirmed the presence of a stable ternary complex with an experimental molecular mass of 192 kDa (Fig EV5A).

Since CDK5RAP3 forms an integral complex with UFL1/UFBP1 and CDK5RAP3 has recently been suggested to function as a substrate adaptor (Yang *et al*, 2019; Stephani *et al*, 2020), we wondered if it could influence E3 ligase activity or substrate UFMylation. We therefore monitored the E3 ligase activity of UFL1/UFBP1 in the presence of increasing concentrations of CDK5RAP3. Surprisingly, incubation of CDK5RAP3 with UFL1/UFBP1 impaired E3 ligase activity and UFMylation in a concentration‐dependent manner (Fig 4B). Moreover, preformed UFL1/UFBP1/CDK5RAP3 complexes purified by SEC also showed no ligase activity (Fig EV5B). These observations imply that CDK5RAP3 binds to and inhibits the ligase complex.

One possibility is that CDK5RAP3 inhibits UFMylation by blocking complex formation between UFL1/UFBP1 and UFC1^‐O‐UFM1^. However, in pull‐downs and analytical SEC, CDK5RAP3 forms a complex together with UFL1/UFBP1 and UFC1^‐O‐UFM1^ (Fig EV5C and D). Since CDK5RAP3 does not affect UFC1~UFM1 binding, we next tested if CDK5RAP3 binding influences the discharge of UFM1 from UFC1~UFM1 by monitoring the transfer of UFM1 onto free Lys. While UFM1 is readily discharged onto Lys in the presence of UFL1/UFBP1, this is completely blocked in the presence of CDK5RAP3 (Figs 4C and D, and EV5E). These observations suggest that binding of CDK5RAP3 to the UFL1/UFBP1/UFC1~UFM1 complex prevents activation of UFC1~UFM1 resulting in inhibition of UFMylation.

While these *in vitro* experiments clearly demonstrate that CDK5RAP3 inhibits E3 ligase activity, it raises the question of the role of CDK5RAP3 in substrate UFMylation. Hence, we used the cell‐free reconstitution of ribosome UFMylation described in Fig 1D and monitored UFMylation of RPL26. In the absence of CDK5RAP3, mono‐ and di‐UFMylated RPL26 species are formed, but with increasing concentrations of CDK5RAP3, the di‐UFMylation of RPL26 is completely abolished (Fig 4E). Importantly, even at the highest concentration of CDK5RAP3, monoUFMylation of RPL26 is not affected. By contrast, the addition of increasing concentrations of CDK5RAP3 decreased UFMylation of other substrates such as H4, MRE11, and TRIP4 (Figs 4F and EV5F). Hence, we propose CDK5RAP3 to be a specificity determinant, keeping the activity of the ligase complex inhibited in the absence of substrate and directing ligase activity towards the ribosomal subunit RPL26.

### Role of UFC1 TAK motif in UFMylation

**Figure 5:**
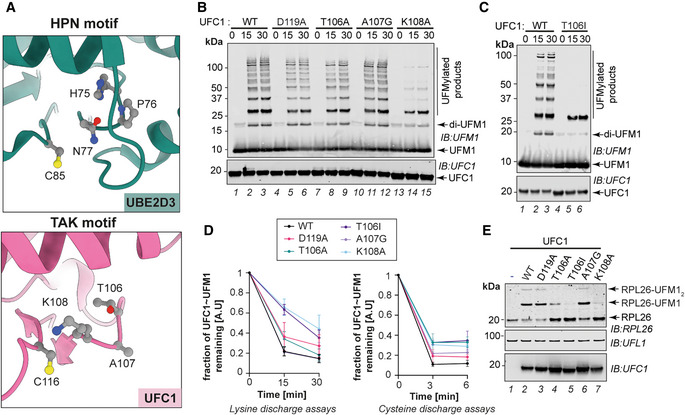
Role of TAK motif in UFC1's activity AComparison of catalytic sites of UBE2D3 (PDB ID:5EGG, shown in green) and UFC1 (PDB ID:2Z6O, shown in pink) shown as cartoons. The residues of the conserved HPN and TAK motif of UBE2D3 and UFC1, respectively, are shown in ball and stick representation.BImmunoblot showing the *in vitro* UFMylation assay to compare the activity of UFC1 Wild type and mutants in the presence of UFL1/UFBP1 complex.C
*In vitro* UFMylation assay to compare the activity of UFC1 Wild type and UFC1 T106I in the presence of UFL1/UFBP1 complex.DQuantification of lysine and cysteine discharge of UFM1 from UFC1 WT and other indicated mutants (*n* = 3 biological replicates, mean ± SD). The first time point in cysteine discharge assays indicated as “0” represents reaction products before the addition of cysteine. Following the addition of 5 mM cysteine, the reaction was incubated at RT for the specified time duration. The reaction was stopped by the addition of nonreducing SDS loading buffer (1× final) and analyzed as described above. A representative gel used for lysine quantification is given in Appendix Fig S1C and D.ESubstrate UFMylation assays using purified 60S Ribosomes in the presence of UFC1 Wild type and different mutants of UFC1. Source data are available online for this figure.

Canonical E2 enzymes contain a conserved HPN motif upstream of the catalytic cysteine (Wu *et al*, 2003; Cook & Shaw, 2012). The Asn in this motif is indispensable for the transfer of the Ub/UBL from the E2~Ub/UBL onto substrate Lys, while the His forms a hydrogen bond with the Asn to stabilize the architecture of the HPN motif (Cook & Shaw, 2012). Intriguingly, UFC1 lacks the oxyanion hole stabilizing Asn as part of the highly conserved HPN motif found in E2 enzymes, which is instead replaced by a TAK motif at this position (Fig 5A, Appendix Fig S1A). However, the importance of the alternative TAK motif is underscored by the identification of T106I mutations in patients with severe early‐onset encephalopathy (Nahorski *et al*, 2018). Having established a UFMylation assay with UFL1/UFBP1, we wanted to ascertain whether TAK motif residues are required for UFMylation. While mutation of T106A and A107G did not affect UFMylation, the K108A mutant completely abolished UFMylation (Fig 5B). In contrast to the T106A mutant, which showed no impact on activity, the disease‐causing T106I mutant showed a dramatic impairment of UFMylation (Fig 5C). Indeed, both the UFMylation deficient mutants T106I and K108A were defective in being activated by the E3 ligase complex for aminolysis (Fig 5D, Appendix Fig S1C), which in turn manifested in reduced RPL26 UFMylation (Fig 5E). However, Cys reactivity of UFC1 is not significantly impacted by mutations to T106 and K108 (Fig 5E, Appendix Fig S1D). In addition to the HPN motif, canonical E2s have a conserved negatively charged residue that activates the attacking substrate lysine (Dou *et al*, 2012; Plechanovová *et al*, 2012). In UFC1, the equivalent residue is D119, mutation of which to Ala did not show significant defects in UFMylation (Fig 5B–E, Appendix Fig S1B). Together, these results reveal that UFC1 potentially utilizes an alternative mechanism for the transfer of UFM1 to the substrate.

### Regulation of UFC1 activity by its N‐terminal helix extension

**Figure 6:**
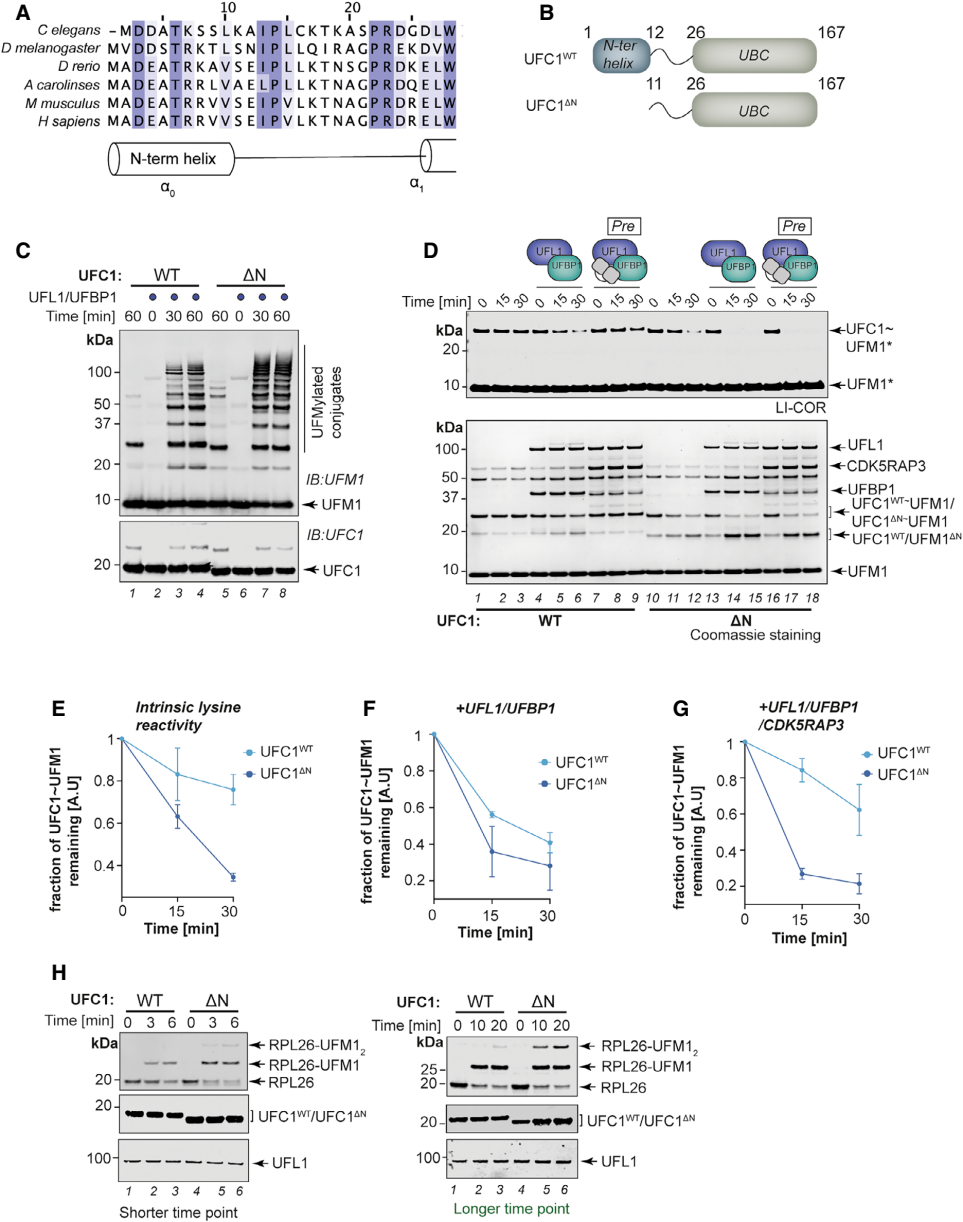
Role of the N‐terminal helix of UFC1 in UFMylation AMultiple sequence alignment of UFC1 homologs from various organisms. A graphical representation of the secondary structure is extracted from the crystal structure of UFC1 (PDB ID:2Z6O).BSchematic representation of domain features of UFC1^WT^ and UFC1^ΔN^.C
*In vitro* UFMylation assays in the presence of Wild type UFC1 (UFC1^WT^) and UFC1 lacking the N‐terminal helix (UFC1^ΔN^).DAssay to compare Lys discharge activities of UFC1^WT^ and UFC1^ΔN^ on its own, in the presence of UFL1/UFBP1 or in the presence of preassembled UFL1/UFBP1/CDK5RAP3 complex. Top gel: LI‐COR scan of fluorescently labeled UFM1 (UFM1*); bottom gel—Coomassie stained (Representative of three independent experiments).E–G(E) Quantitative analysis of the intrinsic Lys reactivity (Lysine 25 mM) of UFC1^WT^ and in UFC1^ΔN^ in the absence of E3 ligase; (F) in the presence of UFL1/UFBP1; (G) in the presence of preassembled UFL1/UFBP1/CDK5RAP3 (E–G: *n* = 3 biological replicates, mean ± SD).HEffect of deletion of N‐term helix of UFC1 in 60S ribosome UFMylation. Purified 60S ribosomes (50 nM) were incubated with 0.5 μM UBA5, 1 μM UFM1, 100 nM UFL1/UFBP1 complex in the presence of either 1 μM UFC1^WT^ or 1 μM UFC1^ΔN^ at 30°C. The reaction was stopped at indicated time points and run on a 4–12% SDS–PAGE gel under reducing conditions followed by immunoblotting using indicated antibodies (Right—shorter time duration. Left—Longer time duration). Source data are available online for this figure.

In addition to its core UBC fold, UFC1 has at its N‐terminus a conserved α‐helix (α0) whose function is unknown (Fig 6A and B). Motivated by the fact that previous studies have shown that the N‐ and C‐ terminal extensions on E2s regulate E2 activity (Stewart *et al*, 2016), we sought to determine whether α0 has a role in regulating UFMylation. We first compared the activity of UFC1^WT^ and UFC1 lacking α0 (UFC1^ΔN^) in UFMylation assays containing UFC1 with or without UFL1/UFBP1. Surprisingly, UFC1^ΔN^ showed stronger overall UFMylation compared with UFC1^WT^ in the presence of UFL1/UFBP1 (Fig 6C). This suggests an inhibitory role for the N‐terminal helical extension of UFC1. Next, we compared the discharge of UFM1 from UFC1~UFM1 and UFC1^ΔN^~UFM1 onto free Lys. In the absence of E3 ligase, UFC1^ΔN^ rapidly discharges UFM1 onto Lys (Fig 6D and E) suggesting an increase in its intrinsic lysine reactivity. In the presence of UFL1/UFBP1, the discharge of UFM1 onto Lys is enhanced when α0 of UFC1 is deleted (Fig 6D and F). Taken together with the increase in UFMylation in the presence of UFL1/UFBP1, these results further strengthen the hypothesis of an inhibitory role for the N‐terminal helix of UFC1.

Since we identified the N‐terminal α0 of UFC1 to restrain UFMylation, we analyzed if inhibition by CDK5RAP3 was mediated via this helix. Indeed, UFMylation assays together with discharge assays comparing the activity of UFC1^WT^ and UFC1^ΔN^ reveal that the inhibition of E3 ligase activity by CDK5RAP3 requires the N‐terminal helix of UFC1 (Fig 6D and G, Appendix Fig S2). As the inhibition mediated by CDK5RAP3 is relieved in the presence of 60S ribosomes, we examined the role of UFC1 α0 on UFMylation of RPL26 (Fig 6H). While ~50% of RPL26 is UFMylated at 6 min in reactions containing UFC1^WT^, near complete RPL26 UFMylation is observed at 3 min with UFC1^ΔN^ (Fig 6H, lane 3 vs. 5). Based on these results we propose that CDK5RAP3 may interact with both UFL1/UFBP1 and α0 of UFC1 to clamp the complex in an inhibited state. Together these analyses reveal a previously unappreciated regulatory role for the N‐terminal helix of UFC1 in modulating UFMylation.

## Discussion

In this study, we establish a robust *in vitro* reconstitution system using purified components of the UFM1 enzymatic pathway to reveal the minimal requirements for UFMylation and mechanistic insights into the ligase machinery. Previous reports have provided conflicting views on the roles of UFBP1 and CDK5RAP3, with some suggesting that they are mainly substrates of UFMylation (Tatsumi *et al*, 2010; preprint: Gak *et al*, 2020). Further, it has been suggested that UFBP1 must be UFMylated first at K267 before it can associate with UFL1 to subsequently support UFL1 ligase activity (Yoo *et al*, 2014). Since both UFL1 and UFBP1 complexes are purified from bacteria, our work unequivocally demonstrates that UFL1 and UFBP1 can associate in the absence of any PTMs and the formation of this complex is essential for it to function as an E3 ligase.

Scaffold‐type E3 ligases such as RING E3 ligases bind to both E2 and substrate to bring them together for substrate transfer. Since UFL1 can bind to both UFC1 and its supposed substrate UFBP1 at the same time, it was suggested that UFL1 may be a scaffold‐type E3 ligase (Komatsu *et al*, 2004). However, direct evidence of whether UFL1 is a scaffold/adaptor‐type E3 ligase or a Cys‐based HECT‐like enzyme was lacking. More recently, a UFMylation assay that relied on UFL1 present in mammalian cell extracts to which *in vitro* generated biotinylated E2~UFM1 thioesters were added, found that UFMylation occurred even when cell extracts were treated with cysteine‐alkylating reagents, further suggesting that UFL1 could be a scaffold‐type E3 ligase (preprint: Gak *et al*, 2020). Our mutational analysis showing that the UFL1/UFBP1 complex lacks a single catalytic Cys together with the ability of the E3 ligase to promote UFC1~UFM1 aminolysis firmly establishes that UFL1/UFBP1 is a scaffold‐type E3 ligase. Further reinforcing this conclusion is the observation that K69‐linkage specificity is imparted by the E2 in ligase‐free di‐UFM1 formation, and this K69‐linkage specificity is unaltered by the E3 ligase. Indeed, this is a feature observed in RING E3 ligases where linkage specificity is determined by the E2 enzyme (Deshaies & Joazeiro, 2009; Deol *et al*, 2019). Since UFL1/UFBP1 promotes aminolysis, we speculate that UFL1/UFBP1 binding induces a closed UFC1~UFM1 conformation akin to RING and atypical SUMO E3 ligases (Pichler *et al*, 2004; Reverter & Lima, 2005; Plechanovová *et al*, 2012; Pruneda *et al*, 2012; Cappadocia *et al*, 2015).

UFC1 differs from prototypical E2s since it lacks the catalytic HPN motif, has an exposed catalytic Cys with a lower pKa, lacks the C‐terminal α‐helix observed in canonical E2s, and has an additional α‐helix at its N‐terminus (α0) (Mizushima *et al*, 2007; Gundogdu & Walden, 2019; Kumar *et al*, 2021). Further, it is unclear whether the canonical “backside” interaction can occur in UFC1 (Brzovic *et al*, 2006; Middleton *et al*, 2017). These unique features of UFC1 and the unconventional features of the ligase complex make it likely that the UFM1 machinery employs a unique mechanism to transfer UFM1 from UFC1 onto the substrate. Interestingly, the UBE2E enzymes also have an intrinsically disordered N‐terminal extension that has an inhibitory role in restricting Ub transfer thereby limiting polyUb chain formation (Schumacher *et al*, 2013). Future work will reveal whether E3 binding induces a closed UFM1~UFM1 conformation and whether α0 impedes this process. Nevertheless, our data seem to suggest that α0 has a regulatory role in potentially preventing UFM1 discharge in the absence of the E3 ligase.

The recent advances in structure prediction (Jumper *et al*, 2021) enabled us to identify the regions of UFL1/UFBP1 essential for ligase activity as comprising a tandem repeat of three WH domains—one full WH domain each from UFL1 and UFBP1 and the composite WH domain formed at the point of interaction between the two proteins. As this region can bind to UFC1~UFM1 and activate the E2 for UFM1 discharge, we define this to be the minimal catalytic domain. Further structural studies will be required to reveal why three WH domains are required to form a functional ligase complex and the identification of linchpin residues in UFL1/UFBP1 that play similar roles to RING E3 ligases to activate the E2 for aminolysis (Plechanovová *et al*, 2012; Pruneda *et al*, 2012). Intriguingly, the anaphase‐promoting complex (APC) subunit APC2 contains a WHB domain that binds the “backside” of UCBH10/UBE2C, located at a face opposite from the catalytic site and is an allosteric site in several E2s (Brzovic *et al*, 2003; Brown *et al*, 2015). This backside binding of the WHB domain is specific to UBE2C and is important for the transfer of Ub onto specific substrates (Brown *et al*, 2015). Our observations also raise the question of the function of the other WH/PCI domains in UFL1/UFBP1 to ligase function. One possibility is that the WH domains may mediate substrate recognition, enable binding to UFM1, or serve either to either regulate recruitment or processivity of chain formation. Given that the WH domain of Cockayne syndrome group B (CSB) can bind to ubiquitin, it is tempting to speculate that one of the predicted WH domains of UFL1/UFBP1 may bind to UFM1 (Takahashi *et al*, 2019). WH domains commonly recognize nucleic acids and are typically found in transcription factors or as protein interaction domains (Harami *et al*, 2013). To our knowledge, this is the first demonstration of catalytic activity mediated by WH domains (Aravind *et al*, 2005), leading us to propose moonlighting functions for other WH domain‐containing proteins. It also raises the possibility that other WH domains could have E3 ligase activity for UFMylation or other UBLs. Since UFL1 has been proposed to have nuclear functions in telomere maintenance and DNA damage response (Lee *et al*, 2019; Qin *et al*, 2019, 2020), another possibility is that the WH domains may mediate DNA binding. A further possibility is the use of the WH domains of UFL1/UFBP1 for recognizing rRNA or translating mRNA during ribosome UFMylation. While our mutational analyses validate the predicted structure of the ligase complex, it is possible that the ligase adopts a completely different conformation when bound to the E2, CDK5RAP3, and substrates. The flexible linkers at the NTR and CTR of UFBP1 and UFL1, respectively (Fig 3A) may enable the complex to adopt alternative conformations.

Our *in vitro* studies show that the formation of UFM1 chains and autoUFMylation is inhibited by CDK5RAP3 (Fig 2C). Indeed, previous studies have observed an altered pattern of UFMylation in the absence of CDK5RAP3 leading to the suggestion that it may be a substrate adaptor (Yang *et al*, 2019). Interestingly, CDK5RAP3 was suggested to function as a sensor for ER stress to induce autophagic degradation of aberrant proteins formed as a result of ribosomal stalling (Stephani *et al*, 2020). Several multidomain and multiprotein E3 ligases such as PARKIN and Cullin Ring Ligases (CRLs) are inhibited and their activation is a carefully orchestrated multistep process (Walden & Rittinger, 2018; Baek *et al*, 2021). Based on our observations, we propose that ligase complexes containing UFL1, UFBP1, and CDK5RAP3 represent an autoinhibited state. The surprising finding that ribosome UFMylation is not abolished in the presence of CDK5RAP3 but rather restricted to monoUFMylation leads us to suggest that CDK5RAP3 regulates ligase activity in the following manner: (i) in the absence of substrate, binding of CDK5RAP3 inhibits E3 ligase activity; (ii) this autoinhibition is relieved when substrates such as the 60S ribosome are encountered. This release from inhibition may involve conformational changes induced upon recognition of structural features on the substrate by UFL1, UFBP1, or CDK5RAP3 to mediate substrate UFMylation. Further, CDK5RAP3 prevents UFM1 from being attached to another UFM1 molecule thereby restricting UFM1 chain formation on substrates. Such multi‐layered regulation possibly prevents spurious UFMylation ensuring ribosome UFMylation only in the right context. While previous studies have suggested roles for UFMylation to facilitate UFL1‐UFBP1 interaction, and UFMylation and phosphorylation to enhance UFMylation (Tatsumi *et al*, 2010; Lemaire *et al*, 2011; Yoo *et al*, 2014; Qin *et al*, 2019), our minimal reconstitution clearly demonstrates that the ligase complex assembles in the absence of any PTM. Although we cannot rule out a role for phosphorylation in enhancing ligase activity, the rapid UFMylation of ribosomes by the reconstituted ligase suggests that this is unlikely.

Our rebuilding approach provides insights into the assembly, minimal requirements, and mechanism of the UFL1/UFBP1/CDK5RAP3 ligase and reveals principles of protein UFMylation. The *in vitro* reconstitution system we describe here using purified components lays the foundation for future biochemical and structural studies to understand the molecular mechanism of this unusual E3 ligase complex and substrate UFMylation. Further, the cell‐free UFMylation system can be applied to understand the logic of ribosome UFMylation and its relationship to ribosome quality control pathways. Ultimately, extending these studies into a cellular setting will be needed to understand how the ligase complex is activated to attach UFM1 onto ribosomes at the ER and how UFMylation is regulated in cells.

## Materials and Methods

### Plasmids, cloning, and mutagenesis

The details of cDNA constructs used in this study are given in Appendix Table S1. Cloning of most of the constructs was performed by MRC‐PPU Reagents and Services (University of Dundee). Briefly, mutagenesis was carried out using Q5 site‐directed mutagenesis kit (NEB) with KOD polymerase (Novagen) according to the manufacturer's protocol. Following mutagenesis, cDNA constructs were amplified using *E. coli* DH5α and purified using QIAprep spin mini‐prep kit (Qiagen). All cDNA constructs were verified by DNA sequencing and services, the University of Dundee using DYEnamic ET terminator chemistry (Amersham Biosciences) on Applied Biosystems automated DNA sequencers.

### Recombinant protein expression and purification

#### Purification of His6‐tagged proteins

Recombinant His_6_‐3C‐UBA5, His_6_‐3C‐UFM1^WT^, His_6_‐3C‐UFM1^Konly^, His_6_‐3C‐UFM1^KtoR^, His_6_‐3C‐Cys‐UFM1^WT^, His_6_‐3C‐UFC1^WT^, His_6_‐3C‐UFC1^ΔN^, and His_6_‐3C‐MRE11 were expressed in *E. coli* BL21 (DE3) and purified using Ni^2+^‐NTA affinity chromatography as the first step. Briefly, *E. coli* BL21 cultures expressing His_6_‐tagged proteins were grown in 2xTY medium at 37°C until OD_600_ reached 0.6–0.8. Final concentration of 0.3 mM IPTG was added and the cultures were incubated at 18°C for 16 h. Cells were harvested, resuspended, and lysed in lysis buffer containing 25 mM Tris–pH 8, 300 mM NaCl, 10% Glycerol, 2 mM DTT, 1 mM Benzamidine, 1 mM AEBSF, 1× protease inhibitor cocktail (Roche) by ultrasonication. Lysed cells were then clarified by centrifugation at 30,000 *g* for 30 min at 4°C. The clarified lysate was then incubated with pre‐equilibrated Ni^2+^‐NTA Agarose beads (Amintra, Abcam) for 2 h in binding buffer containing 25 mM Tris–pH 8, 300 mM NaCl, 10% Glycerol, and 10 mM Imidazole. The beads were then washed extensively using wash buffer containing 25 mM Tris–pH 8, 300 mM NaCl, 10% Glycerol, 2 mM DTT, and 20 mM Imidazole. Finally, the bound protein was eluted using elution buffer containing 25 mM Tris–pH 8, 300 mM NaCl, 10% Glycerol, 2 mM DTT, and 300 mM Imidazole. Wherever necessary, His_6_‐tags were cleaved off by incubating tagged proteins with PreScission protease at 4°C overnight. A final size exclusion chromatography step was performed using HiLoad™ 16/60 Superdex™ 75 pg and HiLoad™ 16/60 Superdex™ 200 pg columns (GE Healthcare Life Sciences) with buffer containing 25 mM Tris–pH 8.0, 150 mM NaCl, 10% Glycerol, and 2 mM DTT. The purified proteins were then concentrated using Amicon™ Ultra 15 concentrators (MERCK Millipore) and stored in −80°C.

#### Purification of GST‐tagged proteins

GST‐TEV‐TRIP4 was expressed in *E. coli* BL21 (DE3) strain as described above. Cells were harvested and lysed in lysis buffer containing 25 mM Tris–pH 7.5, 300 mM NaCl, 10% Glycerol, and 2 mM DTT using ultrasonication. Pre‐equilibrated Glutathione 4B‐sepharose beads (Amintra, abcam) were incubated with clarified lysate for 2 h. The beads were then washed with high salt buffer containing 25 mM Tris–pH 7.5, 500 mM NaCl, 10% glycerol and 2 mM DTT. Further, the beads were washed with low salt buffer containing 25 mM Tris–pH 8, 150 mM NaCl, 10% glycerol, and 2 mM DTT. The protein was then cleaved off the tag by incubation with Precision protease at 4°C overnight. A second ion exchange chromatography step was carried out using Resource Q (6 ml) (GE Healthcare Life Sciences) column with low salt buffer (25 mM Tris–pH 7.5, 150 mM NaCl, 10% glycerol, 2 mM DTT) and high salt buffer (25 mM Tris–pH 7.5, 500 mM NaCl, 10% glycerol, and 2 mM DTT). A final size exclusion chromatography step was performed using HiLoad™ 16/60 Superdex™ 75 pg (GE Healthcare Life Sciences) and the protein was buffer exchanged in buffer 25 mM Tris–pH 7.5, 150 mM NaCl, 10% glycerol, and 2 mM DTT. The purified proteins were then concentrated and stored in −80°C.

#### Co‐expression and purification of UFL1/UFBP1 complex

His_6_‐TEV‐UFL1 and StrepII‐3C‐UFBP1^(29‐end)^ were cloned in a pET Duet1 construct and expressed in *E. coli* BL21 codon plus RIPL (Agilent) cells. Bacterial cultures were grown in 2xTY medium at 37°C until OD_600_ reached 0.6. Final concentration of 0.3 mM IPTG was added to induce the expression and the cultures were incubated at 18°C for 16 h. Cells were harvested and resuspended in buffer containing 25 mM Tris–pH 8.0, 300 mM NaCl, 2 mM DTT, 1 mM Benzamidine, 1 mM AEBSF, and protease inhibitor cocktails (Roche). Cells were lysed by high pressure homogenization using an Emulsiflex C3 homogenizer (Avestin). The lysate was then clarified by Ultracentrifugation at 30,000 *g* for 30 min at 4°C. Affinity purification was carried out in two steps. In the first step, the clarified lysate was passed through HisTrap™ FF (GE Healthcare Life Sciences) column. After binding, the column was washed with 10 column volumes (CVs) of binding buffer containing 25 mM Tris–pH 8.0, 300 mM NaCl, 20 mM Imidazole, and 2 mM DTT. After washing, bound proteins were eluted using buffer containing 25 mM Tris–pH 8.0, 300 mM NaCl, 300 mM Imidazole, and 2 mM DTT by applying a concentration gradient of Imidazole. The eluted proteins were then passed through StrepTrap™ (GE Healthcare Life Sciences) column pre‐equilibrated with binding buffer containing 25 mM Tris–pH 8.0, 300 mM NaCl and 2 mM DTT. After 2 CVs of washing with binding buffer, the proteins were eluted using 25 mM Tris–pH 8.0, 300 mM NaCl, 2 mM DTT, and 2.5 mM Desthiobiotin. Finally, purified proteins were passed through HiLoad™ 16/60 Superdex™ 200 pg (GE Healthcare Life Sciences) and the fractions corresponding to the complex were collected. The purified protein was then stored in −80°C in buffer containing 25 mM Tris–pH 8.0, 300 mM NaCl, 5% Glycerol, and 2 mM DTT until further use.

#### Preparation and labeling of IRDye800CW‐UFM1

Cys‐UFM1^1‐83^ which contains a Cys residue upstream of M1 was purified as described above and exchanged into a fresh buffer containing 50 mM HEPES pH 7.5, 150 mM NaCl and 0.5 mM TCEP using CentriPure P10 columns (EMP Biotech). Protein was then mixed with IRDye® 800CW Maleimide (LI‐COR®) at a 5:1 molar ratio and incubated for 2 h at RT. Unreacted dye was removed by a three‐step buffer exchange process using CentriPure P10 columns (EMP Biotech) (once) and PD‐10 Sephadex G‐25 column (twice) in a sequential manner according to the manufacturer's instruction. Finally, to remove residual unreacted dye material, the labeled protein was dialyzed into buffer containing 25 mM Tris–pH 8, 150 mM NaCl and 2 mM DTT using a dialysis membrane (Thermo Scientific) (MWCO 3000) overnight at 4°C and stored at −80°C until further use.

### Biochemical assays

#### Discharge assays

To analyze the intrinsic reactivity of UFC1, a single turnover discharge assay was employed. Firstly, UFC1 was charged with UFM1^WT^ or labeled UFM1 by incubating 0.5 μM UBA5, 10 μM UFC1 and 20 μM UFM1 in a buffer containing 50 mM HEPES pH 7.5, 50 mM NaCl, 0.5 mM DTT and10 mM MgCl_2_. The charging reaction was initiated by the addition of 10 mM ATP and incubation of the reaction mix at 37°C for 20–30 min. To stop further charging of UFM1 onto UFC1, the reaction was quenched by the addition of 50 mM EDTA and subsequent incubation at RT for 10 min. To initiate discharge, the quenched mixture was incubated with 50 mM lysine (pH 8.0) or other amino acids namely Serine, Threonine, Arginine, and Cysteine at 37°C. The reaction was stopped at each of the time points by the addition of SDS Loading dye without any reducing agent to the reaction mix and run on a 4–12% SDS–PAGE under non‐reducing conditions followed by Coomassie staining or visualization using LI‐COR® Odyssey.

In reactions involving the UBE2D3 and UBE2L3, 0.5 μM UBE1 was incubated with 10 μM UBE2D3/UBE2L3 and 20 μM Ub in reaction buffer containing 50 mM HEPES pH 7.5, 50 mM NaCl, 10 mM ATP and 10 mM MgCl_2_ at 37°C for 20 min. The reaction was quenched, and discharge was analyzed as described above.

In discharge assays involving UFL1/UFBP1 and CDK5RAP3, the quenched reaction mix was added to a cocktail containing 1 μM UFL1/UFBP1 or 1 μM or 2 μM of CDK5RAP3. Discharge was initiated by the addition of 50 mM lysine (pH 8.0) and incubated at 37°C for indicated time duration. The reaction was stopped and analyzed as described above.

#### *In vitro* UFMylation assays

To check for UFMylation *in vitro*, 0.25 μM UBA5, 5 μM UFC1, 1 μM UFL1, and 20–30 μM UFM1 were incubated in reaction buffer containing 50 mM HEPES 7.5, 10 mM MgCl_2_, and 5 mM ATP for 1 h at 37°C. The reaction was stopped by the addition of SDS loading buffer (1× final) containing a reducing agent. The reaction products were then separated on a 4–12% SDS–PAGE gel under reducing conditions and analyzed by immunoblotting using indicated antibodies. In UFMylation assays involving substrates, around 1–2 μM of recombinant substrates were incubated with 0.25 μM UBA5, 5 μM UFC1, 1 μM UFL1, and 30 μM of UFM1 in buffer containing 50 mM HEPES 7.5, 10 mM MgCl_2_, and 5 mM ATP for 1 h at 37°C. Following incubation, the reaction was stopped and analyzed using SDS–PAGE or Immunoblotting using indicated antibodies.

#### Ribosome UFMylation assays

60S ribosomes were a generous gift from the Puglisi lab, and were also purified in‐house as described previously (Johnson *et al*, 2019; Lapointe *et al*, 2021).For reconstituting 60S Ribosome UFMylation, approximately 50 nM purified 60S ribosomes were mixed with, 0.5 μM UBA5, 1 μM UFC1, 0.5 μM UFM1, and 100 nM UFL1/UFBP1 in a reaction buffer containing 25 mM HEPES pH 7.5, 100 mM NaCl, 10 mM MgCl_2_ and 5 mM ATP and incubated at 30°C for 10 min or indicated time duration. The reaction was stopped by the addition of SDS loading buffer (1× final) and run on a 4–12% SDS–PAGE gel under reducing conditions followed by immunoblotting using indicated antibodies.

Ribosome UFMylation assay as described in Fig 4E was performed at 30°C with 1 μM UFC1, 0.5 μM UBA5, 0.5 μM UFM1, 0 nM or 75 nM UFL1/UFBP1, 50 nM purified 60S ribosomes, and increasing concentrations of CDK5RAP3 (0, 38, 75, 150 or 375 nM) in a reaction buffer of 25 mM HEPES pH 7.5, 100 mM NaCl, 10 mM MgCl_2_ and 5 mM ATP. The reaction was quenched by the addition of SDS‐Load buffer (1× final) with reducing agent. Western blots show 10 min reaction time; immunoblots for RPL26 (Abcam, 59567) and UFL1 (Bethyl, A303‐455M) were run on the same gel, which was cut and probed for these proteins separately. Immunoblots for CDK5RAP3 (Bethyl, A300‐871A) and UFM1 (Abcam, Ab109307) were run on separate gels.

#### Preparation of UFC1^‐O‐UFM1^

UFC1^‐O‐UFM1^ was prepared by incubating 40 μM UFC1^C116S^ with 40 μM UFM1, 1 μM UBA5 in buffer containing 50 mM Tris–pH 8.8, 10 mM MgCl_2_, and 5 mM ATP overnight at 25°C. The reaction was incubated briefly with 20 mM DTT to remove any non‐specific di‐sulfide linked adducts formed and passed through HiLoad™ 16/60 Superdex™ 75 pg with buffer containing 25 mM Tris–pH 8.0, 150 mM NaCl, 5% Glycerol and 2 mM DTT. The fractions corresponding to UFC1^‐O‐UFM1^ were collected and analyzed on 4–12% SDS–PAGE gel. Finally, the fractions containing UFC1^‐O‐UFM1^ were pooled, concentrated, and stored in −80°C until further use.

### Pulldown assays

To analyze the interaction of the E3 ligase with the E2, pulldown assays were performed. Approximately 10 μM of untagged UFC1 or UFC1^‐O‐UFM1^ was incubated with 5 μM of full‐length UFL1/UFBP1 in binding buffer containing 25 mM Tris–pH 8.0, 300 mM NaCl, 2 mM DTT for 1 h at 4°C. Following incubation, 30 μl of pre‐equilibrated Streptavidin Sepharose beads (50% slurry) (IBA Life Sciences) was added and further incubated for about 1 h at 4°C. Following binding, centrifuge tubes containing the reaction mix were spun down at 500 *g* for 3 min and the supernatant was discarded to remove unbound proteins. To remove weakly bound proteins, the beads were washed thrice with 1 ml of ice‐cold binding buffer. Finally, the bound proteins were eluted by the addition of binding buffer containing 2.5 mM Desthiobiotin (pH 8.0) and incubation at 4°C for 30 min. The eluates were then analyzed on 4–12% SDS–PAGE under reducing conditions followed by Coomassie staining.

### Immunoprecipitation analysis

HEK293T Ufl1 KO cells were transfected with UFBP1 C‐terminally tagged with streptavidin binding peptide (SBP) together with UFL1WT‐3xFLAG or UFL1L45R‐3xFLAG expressing plasmids using Lipofectamine LTX (Thermo Life Sciences) (related to Fig 3C). 0.5% NP‐40 lysates were subjected to pulldown using M2 anti‐FLAG or streptavidin affinity gels (Sigma‐Aldrich), separated by SDS–PAGE, and immunoblotted with UFBP1 & UFL1 antibodies.

### Analytical gel filtration chromatography analysis

For analyzing the interaction between UFL1/UFBP1 and CDK5RAP3, around 30 μg of UFL1/UFBP1 and 15 μg of CDK5RAP3 were mixed and incubated for 30 min on ice. Then, around 50 μl of the sample was loaded on Superdex™ 200 Increase 3.2/300 column (GE Healthcare Lifesciences) pre‐equilibrated with buffer containing 25 mM Tris–pH 8.0, 300 mM NaCl and 2 mM DTT. The fractions corresponding to each peak were collected and further analyzed on a 4–12% SDS–PAGE under reducing conditions followed by Coomassie staining.

To check whether UFL1/UFBP1 could be reconstituted *in trans*, around 15 μg of purified His_6_‐UFL1 and Untagged UFBP1^(29‐end)^ were mixed and incubated on ice for 2 h. After incubation, the sample was loaded on Superdex™ 200 Increase 3.2/300 column (GE Healthcare Lifesciences) column pre‐equilibrated with buffer containing 25 mM Tris–pH 8.0, 300 mM NaCl, 2 mM DTT, and analyzed as described above.

### Alphafold predictions

The structure of UFL1/UFBP1 was predicted using the ColabFold Google Colab notebook “AlphaFold_advanced” (Jumper *et al*, 2021) (https://github.com/sokrypton/ColabFold). The predicted model with the highest IDDT score is shown in the main figure and the PAE scores for different models ranking 1–5 are shown in Fig EV3A.

### DALI analysis

DALI server was used to perform structural similarity analysis to identify and compare structurally similar proteins (Holm, 2020). The WH1 domain of UFL1 (a.a 52–115) was used as the query model and searched against PDB25 database. The top 10 models in the order of the Z‐score is shown in Fig EV3B.

### Bioinformatic analysis

For the generation of a composite Foldindex profile, a previously reported method was adapted (Prilusky *et al*, 2005; Ahel *et al*, 2020). First, individual Foldindex profiles of UFL1 from different organisms were generated locally using a custom Foldindex program provided by Dr. Juraj Ahel. Then, a composite Foldindex profile was prepared by blending the images in Adobe illustrator with 30% opacity.

For the generation of multiple sequence alignments, the protein sequences of target proteins and their homologs were manually downloaded from UNIPROT database in fasta format. Multiple sequence alignment was performed using Jalview version 2.11.1.7 program (MAFFT algorithm module using L‐INS‐I) (Waterhouse *et al*, 2009; Katoh & Toh, 2010).

### Mass photometry data acquisition and analysis

Protein samples were prepared in a solution containing 25 mM Tris–pH 8.0, 300 mM NaCl, and 2 mM DTT and stored on ice before loading onto the Refeyn One^MP^ instrument (Refeyn). Typically, a set of protein standards (NativeMark™ Unstained Protein Standard, Invitrogen) are used for calibration and to generate a standard curve. Following calibration, approximately 10 μl of diluted protein sample in the concentration range of 5–25 nM was introduced into the flow chamber and movies of 60s duration were recorded. Data acquisition was performed using Acquire MP (Refeyn Ltd, v1.1.3) and analyzed using Discover MP software. Data shown here are the representation of at least three independent acquisitions (*n* ≥ 3).

### SEC‐MALS data acquisition and analysis

Size exclusion chromatography and multiangle light scattering (SEC‐MALS) experiments were performed on a Dionex Ultimate 3000 HPLC system with an inline Wyatt miniDAWN TREOS MALS detector and Optilab T‐rEX refractive index detector. In addition, the elution profile of the protein was monitored with UV 280 attached to the Dionex system. For size exclusion chromatography, Superdex™ 200 Increase 10/300 GL column (GE Healthcare LifeSciences) was used. Fifty microliter of the purified UFL1/UFBP1 stored in buffer containing 25 mM Tris–pH 8.0, 300 mM NaCl and 2 mM DTT was loaded into the SEC column with Dionex autoloader at a concentration of 4 mg/ml and a flow rate of 0.3 ml/min was maintained throughout the experiment. Molar masses spanning elution peaks were calculated using ASTRA v6.0.0.108 (Wyatt).

### LC–MS/MS sample preparation, data acquisition, and analysis

First, an *in vitro* UFMylation reaction was performed to generate UFMylated products including free UFM1 chains. Then, the reaction products were run on a 4–12% SDS–PAGE gel to separate the products based on electrophoretic mobility. Next, the bands corresponding to di‐UFM1 chains were excised and in‐gel digestion was performed according to the previously described protocol (Shevchenko *et al*, 2007). Digested peptides were analyzed by LC–MS/MS on an Exploris 480 coupled to an Ultimate 3000 nanoLC system (Thermo Fisher Scientific) for Fig 2D and on an Exploris 240 (Thermo Fisher Scientific) coupled to an Evosep One (Evosep) for Fig 4D. For the analysis performed on the Ultimate 3000, samples were loaded on a 100 μm × 2 cm trap column (Thermo Fisher Scientific #164564‐CMD) and analyzed on a 75 μm × 50 cm analytical column (Thermo Fisher Scientific #ES903) using a gradient from 3 to 35% Buffer B (80% LC–MS grade acetonitrile, 0.08% formic acid in water) over 53 min. The columns were then washed with 95% Buffer B for 2 min prior to equilibration in 97% Buffer A (0.1% formic acid in LC–MS grade water). For the analysis performed on the Evosep One, samples were loaded onto the Evotips as per manufacturer recommendations and analyzed using the 30 SPD Method. Peptides were then analyzed in on either the Exploris 240 or 480 using data dependant with an MS1 resolution of 60,000, AGC target of 300%, and maximum injection time of 25 or 28 ms. Peptides were then fragmented using TOP 2 s method, MS2 resolution of 15,000, NCE of 30 or 32%, AGC of 100%, and maximum injection time of 100 ms. Peptide identification was performed in Mascot using a restricted and frequently updated database containing ~2,000 protein sequences of interest (MRC db). Carbamidomethylation (C) was set at fixed modification and Oxidation (M), Deoxidation (M) and the addition of the dipeptide Glycine‐Valine (K) were set as variable modifications. Peptides were searched using an MS1 tolerance of 10 ppm and MS2 tolerance of 0.06 Da, and a maximum of two missed cleavages were allowed. Only hits identified with an FDR (false discovery rate) <1% were selected and further analyzed in Scaffold viewer V5. For semi‐quantitative analysis of VG modification on individual sites, total ion chromatogram (TIC) values were obtained from the LC–MS and represented graphically.

## Author contributions

**Joshua J Peter:** Conceptualization; formal analysis; validation; investigation; methodology; writing – original draft; writing – review and editing. **Helge M Magnussen:** Investigation; methodology; writing – review and editing. **Paul A DaRosa:** Investigation; methodology; writing – review and editing. **David Millrine:** Investigation; methodology. **Stephen P Matthews:** Investigation; methodology; writing – review and editing. **Frederic Lamoliatte:** Formal analysis; investigation; writing – review and editing. **Ramasubramanian Sundaramoorthy:** Investigation. **Ron R Kopito:** Funding acquisition; investigation; methodology; writing – review and editing. **Yogesh Kulathu:** Conceptualization; supervision; funding acquisition; investigation; methodology; writing – original draft; project administration; writing – review and editing.

## Disclosure and competing interests statement

The authors declare that they have no conflict of interest.

## Supporting information



Appendix

Expanded View Figures PDF

Source Data for Figure 1

Source Data for Figure 2

Source Data for Figure 3

Source Data for Figure 4

Source Data for Figure 5

Source Data for Figure 6

Source Data for Expanded View and Appendix

PDF+

## Data Availability

This study includes no data deposited in external repositories. Original, uncropped, and unprocessed scans of all gels used in the figures are shown in Source Data.
