# Supplementary material for: A non‐canonical scaffold‐type E3 ligase complex mediates protein UFMylation
Source: EMBO J. 2022 Sep 19;41(21):e111015. doi: 10.15252/embj.2022111015 (PMC9627666; doi:10.15252/embj.2022111015)
Supplement: Supplementary file 1 — Appendix [file EMBJ-41-e111015-s011.pdf]

Appendix

Supplementary S1-S2

Table S1

S1. Role of TAK motif in UFC1 activity

S2. Role of N-terminus of UFC1 in regulating UFMylation

Table S1

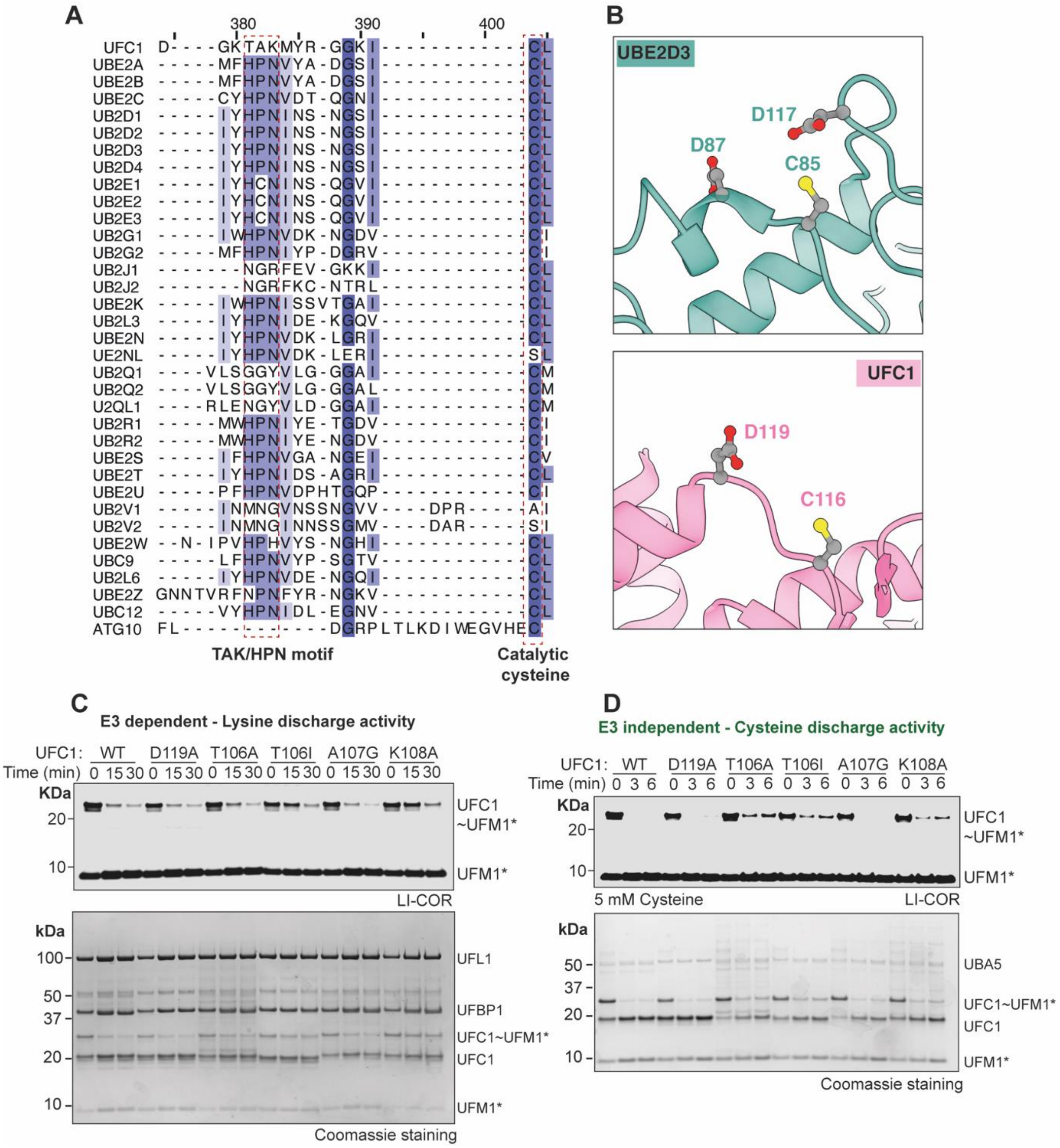

Figure S1:- *Role of TAK motif in UFC1 acitivty*

A. Multiple Sequencing Alignment of human E2 enzymes highlighting the HPN/TAK motif and the catalytic cysteine. Primary sequence of all indicated E2 enzymes were manually downloaded from UNIPROT database and Multiple Sequence Alignment (MSA) was performed using Jalview version 2.11.1.7 programme (MAFFT algorithm module using L-INS-I).

B. 3D structure of UBE2D3 (Top, shown in green, PDB ID:5EGG) and UFC1 (Bottom,shown in pink, PDB ID:2Z6O) shown in cartoon representation highlighting the conserved Aspartate residues (shown in ball and stick representation) in the vicinity of the catalytic cysteine.

C. A representative gel related to figure 5D showing single turnover Lysine discharge assays to compare the activity of UFC1 upon mutation of D119 and conserved TAK motif. Top gel: LICOR scan of fluorescently labelled UFM1(UFM1*); bottom gel – Coomassie stained (Representative of three independent experiments).

D. A representative gel related to figure 5D showing single turnover discharge assays to check for intrinsic cysteine reactivity upon mutation of TAK motif and D119 in UFC1. Top gel: LICOR scan of fluorescently labelled UFM1(UFM1*); bottom gel – Coomassie stained (Representative of three independent experiments).

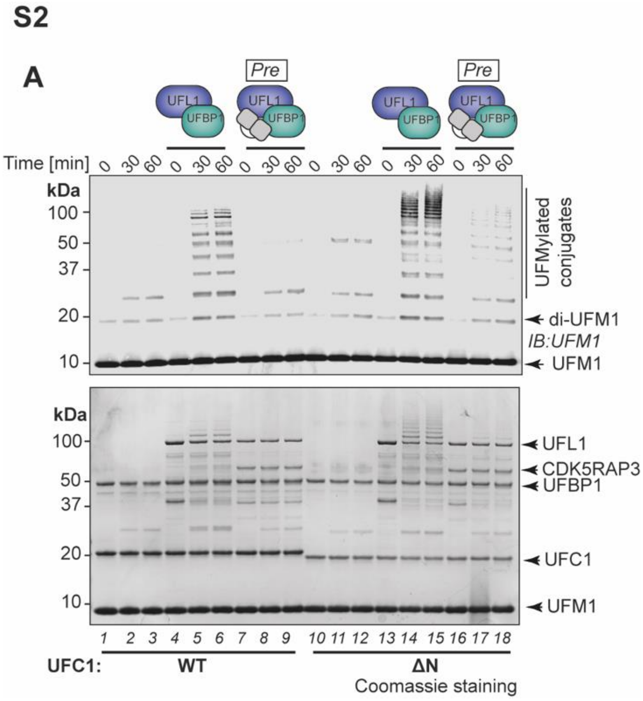

Figure S2:- Role of the N-terminus of UFC1 in regulating UFMylation

A. *In vitro* UFMylation assay to compare the activites of $UFC1^{WT}$ and $UFC1^{\Delta N}$ on its own, in the presence of UFL1/UFBP1 or in the presence of preassembled UFL1/UFBP1/CDK5RAP3 complex (Representative of three independent experiments).

Table S1

REAGENT or RESOURCE	SOURCE	IDENTIFIER
**Antibodies**		
Anti-UFM1	Abcam	ab109305
Anti-UBA5	Universal Biologicals	A304-115A-T
Anti-UFC1	Abcam	ab189252
Anti-UFL1	Abcam	ab227506
Anti-UFBP1	Proteintech	21445-1-AP
Anti-CDK5RAP3	Bethyl Laboratories	A300-870A
Anti-TRIP4	Bethyl Laboratories	A300-203A-M
Anti-MRE11A	Bethyl Laboratories	A300-181A
Anti-H4	Abcam	ab10158
Anti-RPL26	Bethyl laboratories	A300-686A-M
Anti-rabbit IgG, HRP-linked Antibody	CST	70745
IRDye 800CW anti-Rabbit	LI-COR	926-32211
IRDye 680CW anti-Rabbit	LI-COR	926-68071
**Chemicals and other consumables**		
IRDye® 800CW Maleimide	LI-COR	929-80020
Phusion® High-Fidelity DNA Polymerase	New England Biolabs	M0530
Q5® site directed mutagenesis kit	New England Biolabs	E0554S
QIAGEN Plasmid Mini kit	Qiagen Ltd.	12123
dNTPs	Thermo Fisher	R0181
4-(2-aminoethyl) benzene sulphonyl fluoride hydrochloride (AEBSF)	Apollo Scientific	BIMB2003
Benzamidine	Apollo Scientific	1670-14-0
Tris (2-carboxyethyl) phosphine hydrochloride (TCEP)	Apollo Scientific	BIT0122
Isopropyl thio-β-D galactoside (IPTG)	Formedium	IPTG100
Dithiothreitol (DTT)	Formedium	DTT100
Roche cOmplete EDTA-free protease inhibitor cocktail	Roche	11873580001
L-Lysine monohydrochloride	Sigma-Aldrich	L8662-25G
L-Serine	Merck	S4211-25G
L-Threonine	Merck	T0387-10MG
L-Cysteine	Sigma-Aldrich	168149

L-Arginine	Sigma-Aldrich	A8094-100G
Adenosine 5’-triphosphate (ATP)	Apollo Scientific	BIB3003
Ni^{2+}NTA agarose	Amintra, Abcam	ab270549
Glutathione SH-4B sepharose	Amintra, Abcam	Ab270237
Anti-FLAG M2 affinity gel	Sigma-Aldrich	A2220
Streptavidin-agarose	Sigma-Aldrich	S1638
Amylose Resin	New England Biolabs	E8021L
HisTrap FF columns	GE Healthcare Life Sciences	17525501
StrepTrap columns	GE Healthcare Life Sciences	28907547
Strep-Tactin Sepharose resin	IBA Lifesciences	2-1201-010
3C (PreScission) protease	MRC PPU Reagents and Services	DU34905
TEV protease	MRC PPU Reagents and Services	DU6811
**Recombinant proteins**		
MBP-3C-UFL1$^{1-410}$	This Study	DU67192
His_6-3C-UFL1^{FL}	This Study	DU63471
GST-3C-UFL1^{FL}	This Study	DU47296
mMBP-3C-DDRGK1	This Study	DU59678
His_6-3C-UBA5	This Study	DU32106
GST-3C-UFC1	This Study	DU55394
GST-3C-UFC1^{C116S}	This Study	DU59294
His_6-3C-UFC1^{WT}	This Study	DU59469
His_6-3C-UFC1$^{\Delta N}$	This Study	DU72732
His_6-3C-UFC1^{T106A}	This Study	DU72736
His_6-3C-UFC1^{T106I}	This Study	DU72737
His_6-3C-UFC1^{A107G}	This Study	DU72735
His_6-3C-UFC1^{K108A}	This Study	DU72735
His_6-3C-UFC1^{D119A}	This Study	DU68535
GST-3C-UFM1$^{(1-83)\ K0}$	This Study	DU59472
GST-3C-UFM1$^{(1-83)\ K3R}$	This Study	DU59442
His_6-3C-UFM1$^{(1-83)\ K7R}$	This Study	DU59443
His_6-3C-UFM1$^{(1-83)\ K19R}$	This Study	DU59444
His_6-3C-UFM1$^{(1-83)\ K34R}$	This Study	DU59445
His_6-3C-UFM1$^{(1-83)\ K41R}$	This Study	DU59562

His$_6$-3C-UFM1$^{(1\text{-}83)\ K69R}$	This Study	DU59446
His$_6$-TEV-Cys-UFM1$^{(1\text{-}83)}$	This Study	DU55017
His$_6$-3C-UFM1$^{(1\text{-}83)\ K3\ only}$	This Study	DU72726
His$_6$-3C-UFM1$^{(1\text{-}83)\ K7\ only}$	This Study	DU72727
His$_6$-3C-UFM1$^{(1\text{-}83)\ K19\ only}$	This Study	DU72728
His$_6$-3C-UFM1$^{(1\text{-}83)\ K34\ only}$	This Study	DU72729
His$_6$-3C-UFM1$^{(1\text{-}83)\ K41\ only}$	This Study	DU72730
His$_6$-3C-UFM1$^{(1\text{-}83)\ K69\ only}$	This Study	DU72731
His$_6$-TEV-UFL1^{FL} /StrepII-3C-DDRGK1$^{29\text{-}end}$	This Study	DU63479
His$_6$-TEV-UFL1$^{(1\text{-}410)}$ /StrepII-3C-DDRGK1$^{29\text{-}end}$	This Study	DU66224
His$_6$-TEV- UFL1$^{(1\text{-}212)}$ /StrepII-3C-DDRGK1$^{29\text{-}end}$	This Study	DU66218
His$_6$-TEV-UFL1$^{(1\text{-}179)}$ /StrepII-3C-DDRGK1$^{29\text{-}end}$	This Study	DU73844
His$_6$-TEV-UFL1$^{(1\text{-}116)}$ /StrepII-3C-DDRGK1$^{29\text{-}end}$	This Study	DU73843
His$_6$-TEV-UFL1^{FL} /StrepII-DDRGK1$^{207\text{-}end}$	This Study	DU73842
His$_6$-TEV-UFL1$^{(1\text{-}410)}$/StrepII-3C-DDRGK1$^{207\text{-}end}$	This Study	DU72733
His$_6$-TEV-UFL1$^{(1\text{-}179)}$/StrepII-3C-DDRGK1$^{207\text{-}end}$	This Study	DU73851
His$_6$-TEV-UFL1$^{(1\text{-}116)}$/StrepII-3C-DDRGK1$^{207\text{-}end}$	This Study	DU73845
His$_6$-TEV-UFL1$^{C32A(1\text{-}410)}$/StrepII-3C-DDRGK1 $^{29\text{-}end}$	This Study	DU66308
His$_6$-TEV-UFL1$^{C143A(1\text{-}410)}$/StrepII-3C-DDRGK1 $^{29\text{-}end}$	This Study	DU66287
His$_6$-TEV-UFL1$^{C300A(1\text{-}410)}$/StrepII-3C-DDRGK1 $^{29\text{-}end}$	This Study	DU66288
His$_6$-TEV-UFL1$^{C374A(1\text{-}410)}$/StrepII-3C-DDRGK1 $^{29\text{-}end}$	This Study	DU66289
His$_6$-TEV-UFL1$^{(1\text{-}410)\ C32A,C143A,C300A,C372A}$ /StrepII-3C-DDRGK $^{29\text{-}end}$	This Study	DU66290
MBP-3C-CDK5RAP3	This Study	DU59674
His$_6$-3C-MRE11$^{(1\text{-}411)}$	This Study	DU66124
GST-TEV-TRIP4	This Study	DU59564
UBE1$^{(2\text{-}1058)}$	MRC PPU Reagents and Services	DU3026
UBE2D3	MRC PPU Reagents and Services	DU15703
UBE2L3	MRC PPU Reagents and Services	DU3772
