# Supplementary material for: A non‐canonical scaffold‐type E3 ligase complex mediates protein UFMylation
Source: EMBO J. 2022 Sep 19;41(21):e111015. doi: 10.15252/embj.2022111015 (PMC9627666; doi:10.15252/embj.2022111015)
Supplement: Supplementary file 2 — Expanded View Figures PDF [file EMBJ-41-e111015-s006.pdf]

Expanded View Figures

Figure EV1. ***In vitro*** **reconstitution of the active UFM1 E3 ligase.**

A Size exclusion chromatography profile of UFL1 run on Superdex™ 200 Increase 10/300 GL column (shown in red). Overlay of UV chromatogram of molecular weight standards of different sizes (shown in grey) run under same buffer conditions.
B Results obtained from ULTIMATE Y2H™ screening (Hybrigenics) for binary interactions with UFL1. Asterisk (*) denotes proteins hits obtained with low confidence.
C Schematic describing the strategy for co-expression and purification of UFL1/UFBP1 complex.
D SEC-MALS analysis of UFL1/UFBP1 complex. The theoretical and observed molecular weights are indicated above.
E Analytical gel filtration chromatography analysis showing UV traces of UFL1 and UFBP1 run on their own and a mixture containing UFL1 and UFBP1. Superdex™ 200 Increase 3.2/300 column was used for analysis.
F UFBP1 does not activate UFL1 when added exogenously. *In vitro* UFMylation assays to compare the E3 ligase activity of UFL1 and UFBP1 expressed alone and together as a complex. 0.25 μM UBA5, 5 μM UFC1 and 10 μM UFM1 was incubated with 1 μM UFL1 or 1 μM UFBP1 or 1 μM UFL1/UFBP1 complex for 1 h at 37°C in the buffer containing 50 mM HEPES 7.5, 0.5 mM DTT, 10 mM $MgCl_2$ and 10 mM ATP. The reaction was stopped by addition of 3× SDS loading dye and run on a 4–12% denaturing SDS PAGE gel under reducing conditions and immunoblotting was performed using indicated antibodies.
G MS^2 spectra showing the peptide derived from *in vitro* UFMylation assay showing the VG-remnant on K69 of UFM1.

Source data are available online for this figure.

© 2022 The Authors

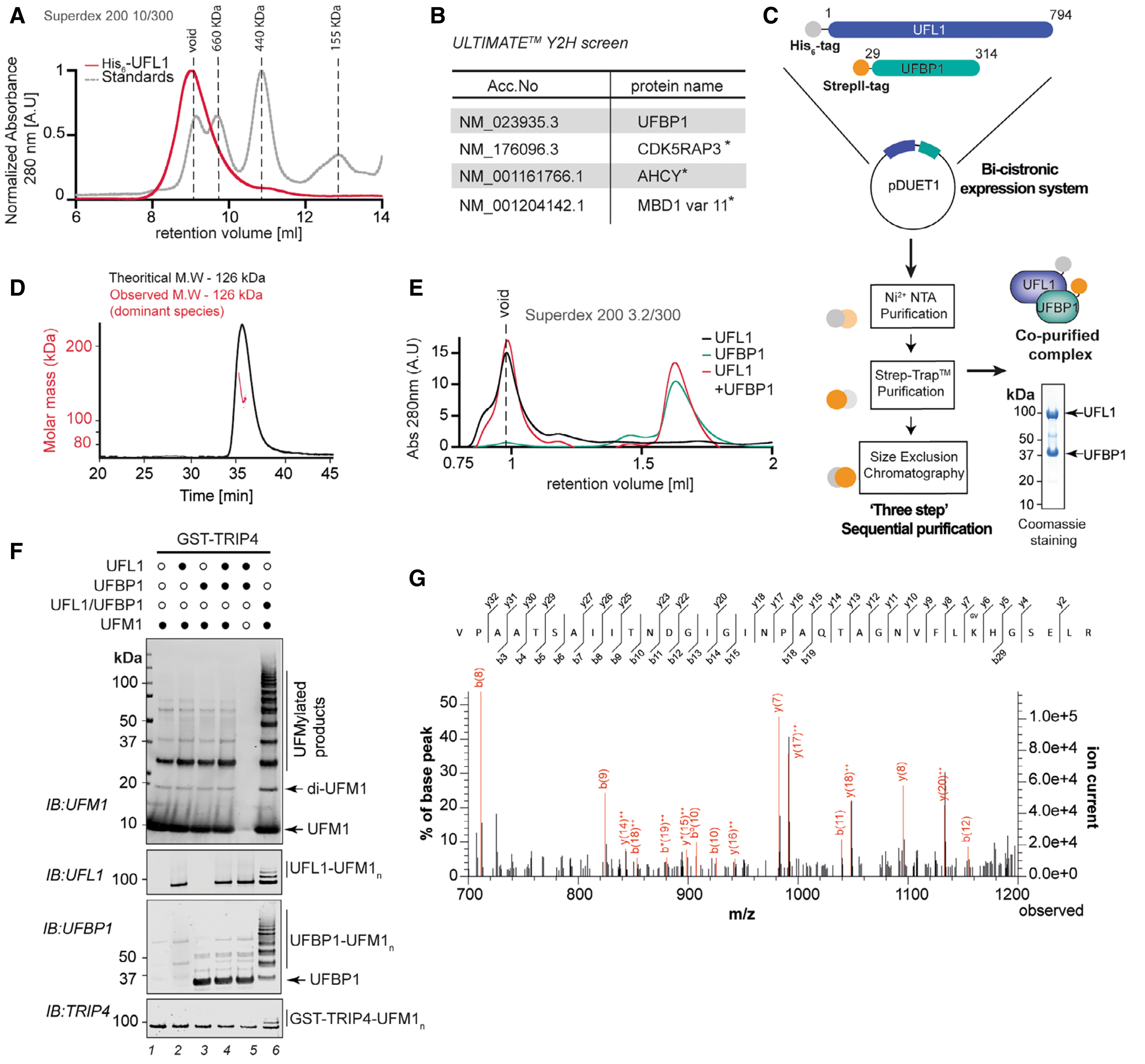
A
Superdex 200 10/300
void
660 KDa
440 KDa
155 KDa
His6-UFL1
Standards
Normalized Absorbance 280 nm [A.U]
retention volume [ml]
B
ULTIMATE™ Y2H screen
Acc.No
protein name
NM_023935.3
UFBP1
NM_176096.3
CDK5RAP3*
NM_001161766.1
AHCY*
NM_001204142.1
MBD1 var 11*
C
UFL1
His6-tag
UFBP1
StrepII-tag
pDUET1
Bi-cistronic expression system
Ni2+ NTA Purification
Strep-Trap™ Purification
Size Exclusion Chromatography
'Three step' Sequential purification
Co-purified complex
kDa
UFL1
UFBP1
Coomassie staining
D
Theoritical M.W - 126 kDa
Observed M.W - 126 kDa
(dominant species)
Molar mass (kDa)
Time [min]
E
void
Superdex 200 3.2/300
UFL1
UFBP1
UFL1 +UFBP1
Abs 280nm (A.U)
retention volume [ml]
F
GST-TRIP4
UFL1
UFBP1
UFL1/UFBP1
UFM1
kDa
UFMylated products
di-UFM1
UFM1
IB:UFM1
IB:UFL1
UFL1-UFM1n
IB:UFBP1
UFBP1-UFM1n
UFBP1
IB:TRIP4
GST-TRIP4-UFM1n
G
V P A A T S A I I T N D G I G I N P A Q T A G N V F L K H G S E L R
% of base peak
ion current
m/z
observed

Figure EV1.

Figure EV2. UFL1/UFBP1 is a scaffold-type E3 ligase.

A Single turnover assay to monitor discharge of Ubiquitin from UBE2D3 and UBE2L3 in the presence of free amino acids. “0” indicates time 0.
B Single turnover lysine discharge assays using UBE2D3 and UFC1 in the presence of increasing concentration of free Lysine.
C Time-dependent analysis of discharge of UFM1 from UFC1 in the presence of high concentration of free lysine (150 mM).
D, E Time-dependent analysis of discharge of Ubiquitin from UBE2D3 and UBE2L3 in the presence of 150 mM Lysine.
F Composite Foldindex profile of UFL1 showing folding propensity of different regions of UFL1 to aid in construct design for soluble protein expression.
G Size exclusion chromatography profile of MBP-UFL1$^{1\text{-}410}$ (dark blue) and His$_6$-UFL1$^{1\text{-}410}$/UFBP1$^{29\text{-end}}$ (orange) run on Superdex™ 200 Increase 3.2/300 column. Approximately 20 μg of sample was used for analysis. (Right) Coomassie stained gel showing the purity of the proteins.
H *In vitro* UFMylation assay to check for E3 ligase activity of UFL1$^{1\text{-}410}$/UFBP1$^{29\text{-end}}$. Full length UFL1/UFBP1 complex is used as a positive control.
I Time dependent analysis of transthiolation activity of UFL1. Reaction products were analysed on a 4–12% SDS PAGE gel under reducing (lane 1–5) and non-reducing conditions (lanes 6–10).
J *In vitro* UFMylation assays to check for formation of di-UFM1 chains by minimal reconstitution using UBA5, UFC1 and UFM1 in the presence of $MgCl_2$ and ATP.
K Immunoblot to check for the presence of free UFM1 chains with and without treatment of UFSP2. *In vitro* UFMylation products generated as shown in (E) was incubated with UFSP2 (2 μM) for 1 h at 37°C. The reaction was stopped by addition of SDS-loading buffer (1× final) and run on a 4–12% SDS PAGE gel under reducing conditions followed by immunoblotting using indicated antibodies to check for the disappearance of polyUFMylated products especially di-UFM1.
L Graphical representation showing the composition of linkage forms of di-UFM1 chains formed by minimal reconstitution of UBA5 and UFC1.

Source data are available online for this figure.

 © 2022 The Authors

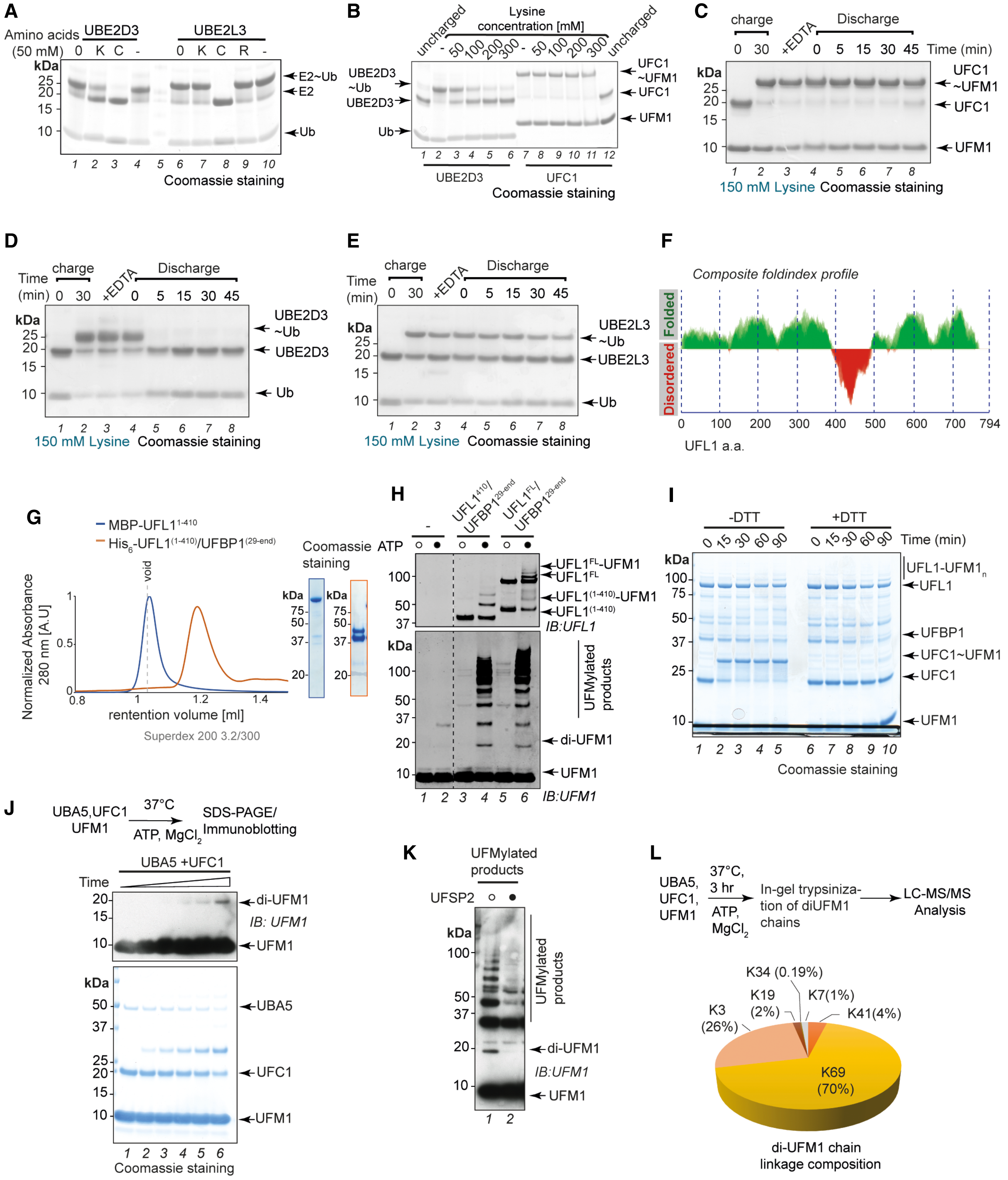
A
Amino acids (50 mM)
UBE2D3
UBE2L3
0 K C -
0 K C R -
kDa
25
20
15
10
E2~Ub
E2
Ub
1 2 3 4 5 6 7 8 9 10
Coomassie staining
B
uncharged
Lysine concentration [mM]
- 50 100 200 300 - 50 100 200 300
uncharged
UBE2D3 ~Ub
UBE2D3
Ub
UFC1 ~UFM1
UFC1
UFM1
1 2 3 4 5 6 7 8 9 10 11 12
UBE2D3
UFC1
Coomassie staining
C
charge
+EDTA
Discharge
0 30
0 5 15 30 45 Time (min)
kDa
UFC1 ~UFM1
UFC1
UFM1
1 2 3 4 5 6 7 8
150 mM Lysine Coomassie staining
D
Time (min)
charge
+EDTA
Discharge
UBE2D3 ~Ub
UBE2D3
Ub
150 mM Lysine Coomassie staining
E
UBE2L3 ~Ub
UBE2L3
Ub
150 mM Lysine Coomassie staining
F
Composite foldindex profile
Folded
Disordered
0 100 200 300 400 500 600 700 794
UFL1 a.a.
G
MBP-UFL1 1-410
His6-UFL1(1-410)/UFBP1(29-end)
void
Normalized Absorbance 280 nm [A.U]
rentention volume [ml]
Superdex 200 3.2/300
Coomassie staining
H
-
UFL1 410/ UFBP1 29-end
UFL1 FL/ UFBP1 29-end
ATP
UFL1FL-UFM1
UFL1FL
UFL1(1-410)-UFM1
UFL1(1-410)
IB:UFL1
UFMylated products
di-UFM1
UFM1
1 2 3 4 5 6 IB:UFM1
I
-DTT
+DTT
0 15 30 60 90
Time (min)
UFL1-UFM1n
UFL1
UFBP1
UFC1~UFM1
UFC1
UFM1
1 2 3 4 5 6 7 8 9 10
Coomassie staining
J
UBA5,UFC1 UFM1
37°C
ATP, MgCl2
SDS-PAGE/ Immunoblotting
UBA5 +UFC1
Time
di-UFM1
IB: UFM1
UFM1
UBA5
UFC1
UFM1
1 2 3 4 5 6
Coomassie staining
K
UFMylated products
UFSP2
di-UFM1
IB:UFM1
UFM1
1 2
L
UBA5, UFC1, UFM1
37°C, 3 hr
ATP, MgCl2
In-gel trypsinization of diUFM1 chains
LC-MS/MS Analysis
K34 (0.19%)
K19 (2%)
K7(1%)
K3 (26%)
K41(4%)
K69 (70%)
di-UFM1 chain linkage composition

Figure EV2.

Figure EV3. Identifying the minimal ligase domain of UFL1/UFBP1 complex.

A Predicted Aligned Error (PAE) scores of UFL1/UFBP1 models predicted using AlphaFold.
B List of structurally similar proteins from the PDB25 database predicted using UFL1 WH1 (52–115 a.a) domain as query structure using DALI server.
C Overlaid structures of Winged helix 1 (WH1) domain of UFL1 (52–115 a.a) and top three hits obtained from DALI search shown in cartoon representation.
D Comparison of winged helix domains of UFL1 (shown in blue) and UFBP1 (shown in teal) to highlight their structural similarities.
E SEC elution profiles showing that full length UFL1/UFBP1 preferentialy interacts with charged E2. UFL1/UFBP1 complex was incubated with UFC1$^{\text{-O-UFM1}}$ at a 1:1 molar ratio for 20 min at 4°C and loaded on a Superdex™ 200 Increase 3.2/300 column. The fractions corresponding to each peak were collected and separated on a 4–12% SDS PAGE gel followed by Coomassie staining.
F Minimal catalytic region is sufficient for interaction with charged UFC1. UFL1$^{(1\text{-}179)}$/UFBP1$^{(1\text{-}116)}$ complex was incubated with UFC1$^{\text{-O-UFM1}}$ at the molar ratio of 1:1 for 20 min at 4°C and analysed by analytical size exclusion chromatography as described in (E).
G Coomassie stained gel showing purity of different UFL1/UFBP1 truncations.

Source data are available online for this figure.

 © 2022 The Authors

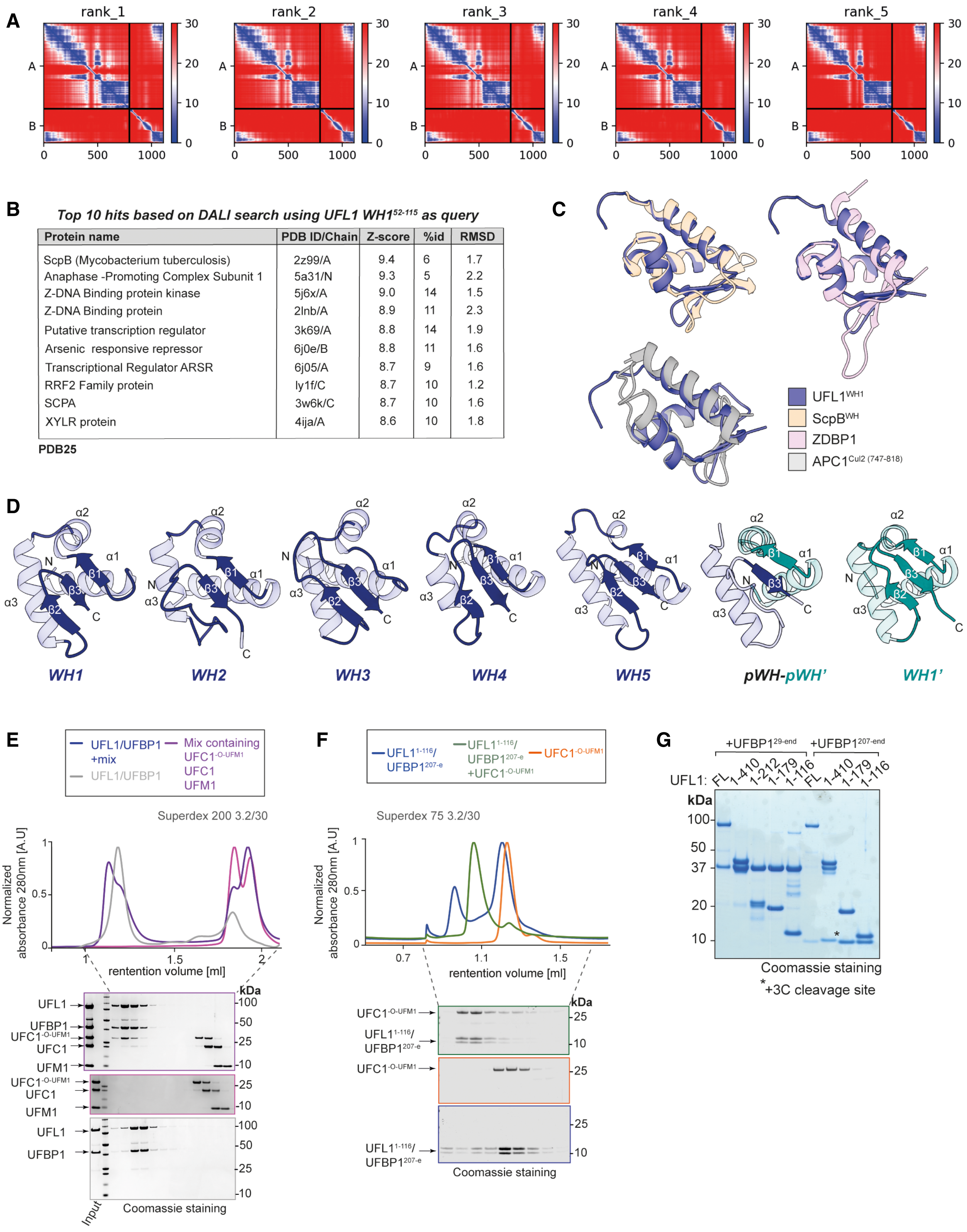
A
rank_1
rank_2
rank_3
rank_4
rank_5
B
Top 10 hits based on DALI search using UFL1 WH1^52-115 as query

Protein name	PDB ID/Chain	Z-score	%id	RMSD
ScpB (Mycobacterium tuberculosis)	2z99/A	9.4	6	1.7
Anaphase -Promoting Complex Subunit 1	5a31/N	9.3	5	2.2
Z-DNA Binding protein kinase	5j6x/A	9.0	14	1.5
Z-DNA Binding protein	2lnb/A	8.9	11	2.3
Putative transcription regulator	3k69/A	8.8	14	1.9
Arsenic responsive repressor	6j0e/B	8.8	11	1.6
Transcriptional Regulator ARSR	6j05/A	8.7	9	1.6
RRF2 Family protein	ly1f/C	8.7	10	1.2
SCPA	3w6k/C	8.7	10	1.6
XYLR protein	4ija/A	8.6	10	1.8

PDB25
C
UFL1^WH1
ScpB^WH
ZDBP1
APC1^Cul2 (747-818)
D
WH1
WH2
WH3
WH4
WH5
pWH-pWH'
WH1'
E
UFL1/UFBP1 +mix
UFL1/UFBP1
Mix containing UFC1^-O-UFM1 UFC1 UFM1
Superdex 200 3.2/30
Normalized absorbance 280nm [A.U]
rentention volume [ml]
UFL1
UFBP1
UFC1^-O-UFM1
UFC1
UFM1
kDa
Input
Coomassie staining
F
UFL1^1-116/ UFBP1^207-e
UFL1^1-116/ UFBP1^207-e +UFC1^-O-UFM1
UFC1^-O-UFM1
Superdex 75 3.2/30
Normalized absorbance 280nm [A.U]
rentention volume [ml]
Coomassie staining
G
+UFBP1^29-end
+UFBP1^207-end
UFL1: FL 1-410 1-212 1-179 1-116 FL 1-410 1-179 1-116
kDa
Coomassie staining
* +3C cleavage site

Figure EV3.

Figure EV4. Identifying the minimal ligase domain of UFL1/UFBP1 complex.

A Lysine discharge assays to check for activation of UFC1 in the presence of different UFL1 truncations. Top gel: LI-COR scan of fluorescently labelled UFM1 (UFM1*); bottom gel—Coomassie stained (representative of three independent experiments); Related to Fig 3F.

B Lysine discharge assays as in (A) in the absence of NTR of UFBP1. Top gel: LI-COR scan of fluorescently labelled UFM1 (UFM1*); bottom gel—Coomassie stained (representative of three independent experiments); Related to Fig 3F.

C Coomassie stained gel showing analysis of *in vitro* UFMylation reaction products to check for the formation of stable oxy-ester linked UFC1-UFM1 conjugate ($UFC1^{-O-UFM1}$).

D Chromatogram obtained from SEC analysis using HiLoad™ 16/60 Superdex™ 75 pg column. (Bottom left) The fractions collected were run on an SDS-PAGE gel to identify fractions that contained pure $UFC1^{-O-UFM1}$. (Bottom right) Coomassie stained gel showing analysis of purified $UFC1^{-O-UFM1}$ product to check for homogeneity.

E Quality check to analyse if UFC1-UFM1 conjugate is linked through an oxy-ester linkage by alkaline hydrolysis.

F, G Substrate UFMylation assays to check for UFMylation of MRE11 and Histone H4 respectively in the presence of different UFL1/UFBP1 truncations.

H Role of UFBP1 in substrate UFMylation. (*Top*) Comparison of E3 ligase activity of UFL1/$UFBP1^{29\text{-end}}$ and UFL1/$UFBP1^{207\text{-end}}$ using Histone H4. (*Bottom*) Comparison of E3 ligase activity of $UFL1^{1\text{-}179}$/$UFBP1^{29\text{-end}}$ and $UFL1^{1\text{-}179}$/$UFBP1^{207\text{-end}}$ using purified Histone H4.

I Schematic showing the tandem WH domains which constitute the minimal ligase domain.

Source data are available online for this figure.

 © 2022 The Authors

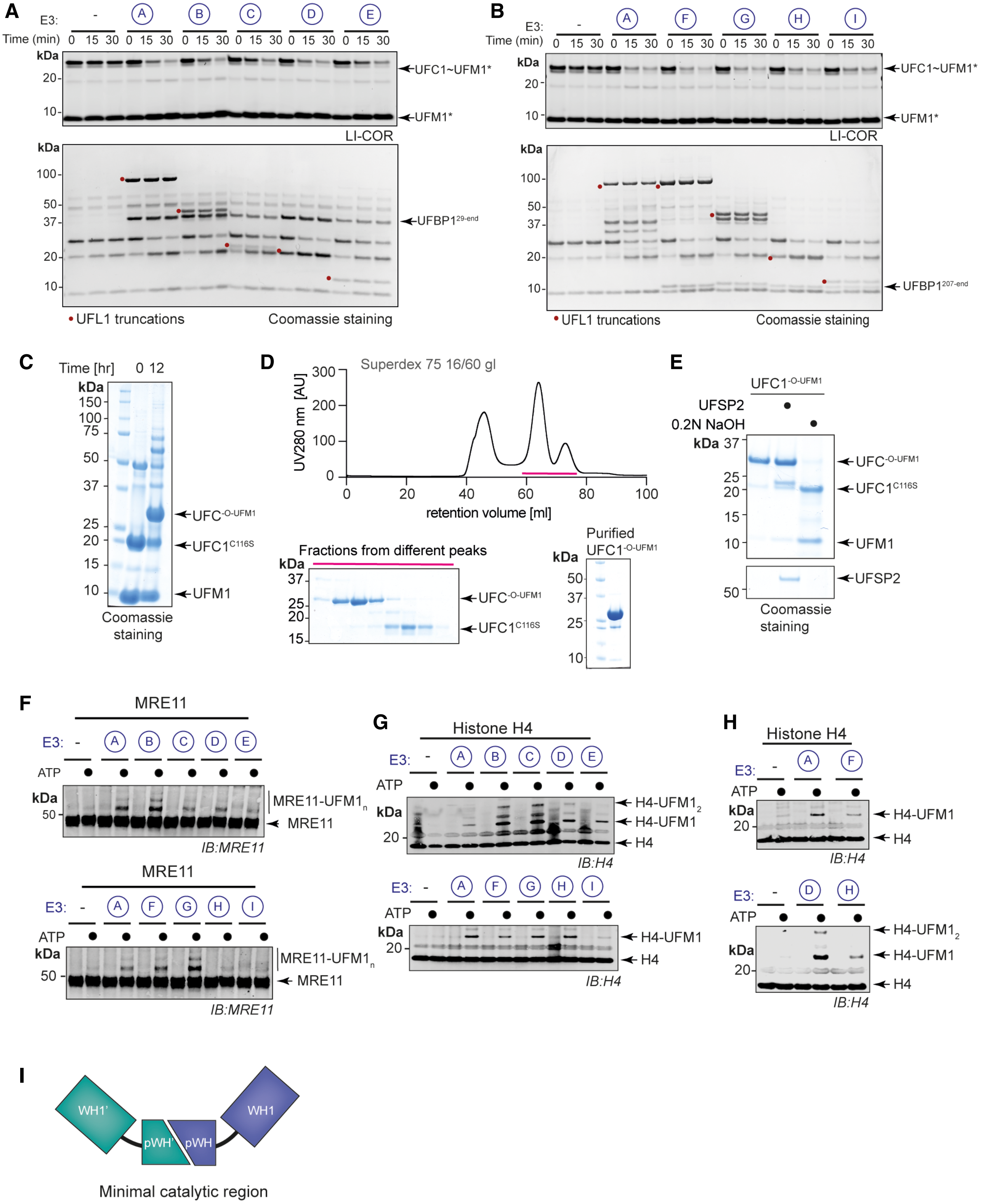
A
E3: - A B C D E
Time (min) 0 15 30 0 15 30 0 15 30 0 15 30 0 15 30 0 15 30
kDa
20
10
UFC1~UFM1*
UFM1*
LI-COR
kDa
100
50
37
20
10
UFBP1 29-end
• UFL1 truncations
Coomassie staining
B
E3: - A F G H I
Time (min) 0 15 30 0 15 30 0 15 30 0 15 30 0 15 30 0 15 30
kDa
20
10
UFC1~UFM1*
UFM1*
LI-COR
kDa
100
50
37
20
10
UFBP1 207-end
• UFL1 truncations
Coomassie staining
C
Time [hr] 0 12
kDa
150
100
75
50
37
25
20
15
10
UFC-O-UFM1
UFC1 C116S
UFM1
Coomassie staining
D
Superdex 75 16/60 gl
UV280 nm [AU]
300
200
100
0
0 20 40 60 80 100
retention volume [ml]
Fractions from different peaks
kDa
37
25
20
15
UFC-O-UFM1
UFC1 C116S
Purified UFC1-O-UFM1
kDa
50
37
25
10
E
UFC1-O-UFM1
UFSP2
0.2N NaOH
kDa 37
25
20
15
10
50
UFC-O-UFM1
UFC1 C116S
UFM1
UFSP2
Coomassie staining
F
MRE11
E3: - A B C D E
ATP
kDa
50
MRE11-UFM1n
MRE11
IB:MRE11
MRE11
E3: - A F G H I
ATP
kDa
50
MRE11-UFM1n
MRE11
IB:MRE11
G
Histone H4
E3: - A B C D E
ATP
kDa
20
H4-UFM1 2
H4-UFM1
H4
IB:H4
E3: - A F G H I
ATP
kDa
20
H4-UFM1
H4
IB:H4
H
Histone H4
E3: - A F
ATP
kDa
20
H4-UFM1
H4
IB:H4
E3: - D H
ATP
kDa
20
H4-UFM1 2
H4-UFM1
H4
IB:H4
I
WH1'
pWH'
pWH
WH1
Minimal catalytic region

Figure EV4.

Figure EV5. CDK5RAP3 forms a complex with UFL1/UFBP1 and inhibits ligase activity.

A Mass photometry analysis showing the experimental molecular weight of UFL1/UFBP1/CDK5RAP3 complex.
B *In vitro* UFMylation assay to compare the E3 ligase activity of UFL1/UFBP1 mixed with CDK5RAP3 and preassembled ternary E3 ligase complex containing UFL1/UFBP1/CDK5RAP3. Ponceau-stained nitrocellulose membrane is shown below to indicate the amounts of reaction components used in the assay.
C UV traces of gel filtration chromatogram showing co-migration of (UFL1/UFBP1)/CDK5RAP3/UFC1$^{\text{-O-UFM1}}$ complex. Approximately, 200 μl of sample containing (UFL1/UFBP1)/CDK5RAP3/UFC1$^{\text{-O-UFM1}}$ in the molar ratio of 1:1.5:3 was mixed and incubated at 4°C for 1 h and loaded onto a Superdex™ 200 Increase 10/300 GL column. The fractions were collected and analysed on a 4–12% SDS PAGE and visualized by Coomassie staining.
D Pulldown assay to check for interaction of UFL1/UFBP1 with charged UFC1 in the presence of absence of CDK5RAP3. Around 10 μM of Untagged UFC1 and 10 μM of UFC1-O-UFM1 were mixed with 5 μM of UFL1/UFBP1 complex in the presence and absence of CDK5RAP3.
E Single turnover lysine discharge assays to check for UFC1 discharge in the presence of UFL1/UFBP1 mixed with CDK5RAP3 and preassembled UFL1/UFBP1/CDK5RAP3 complex. The reaction products were run on a 4–12% SDS PAGE analysis and visualized by Coomassie staining.
F *In vitro* UFMylation assay to monitor UFMylation of purified substrates namely MRE11A (left) and TRIP4 (right) in the presence of increasing concentration of CDK5RAP3 (1, 2, 3 μM). UFL1/UFBP1 concentration—1 μM.

Source data are available online for this figure.

 © 2022 The Authors

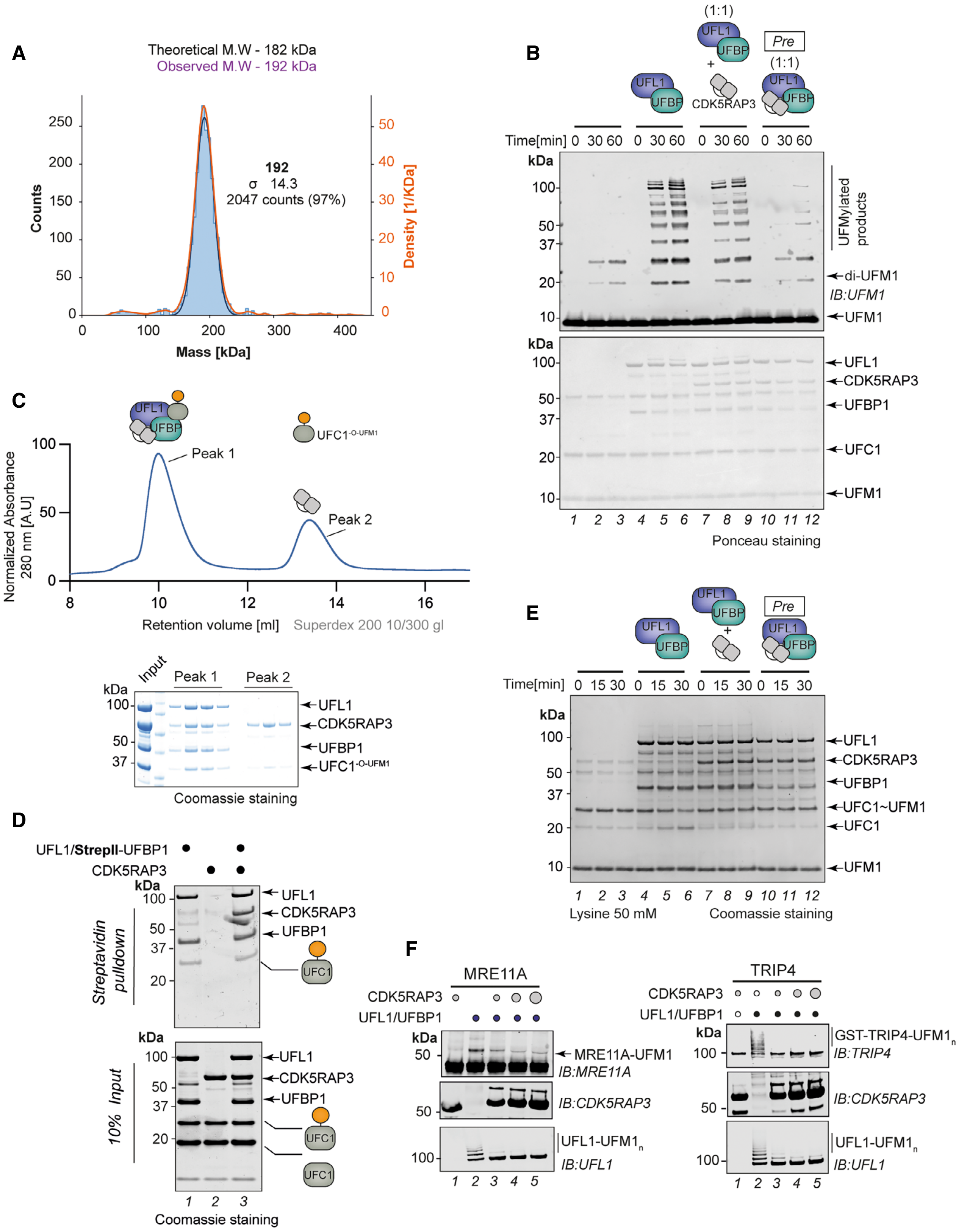
A
Theoretical M.W - 182 kDa
Observed M.W - 192 kDa
192
σ 14.3
2047 counts (97%)
Counts
Density [1/KDa]
Mass [kDa]
B
(1:1)
UFL1
UFBP
CDK5RAP3
Pre
Time[min] 0 30 60 0 30 60 0 30 60 0 30 60
kDa
UFMylated products
di-UFM1
IB:UFM1
UFM1
UFL1
CDK5RAP3
UFBP1
UFC1
1 2 3 4 5 6 7 8 9 10 11 12
Ponceau staining
C
Peak 1
Peak 2
UFC1-O-UFM1
Normalized Absorbance 280 nm [A.U]
Retention volume [ml]
Superdex 200 10/300 gl
Input
Coomassie staining
D
UFL1/StrepII-UFBP1
CDK5RAP3
Streptavidin pulldown
10% Input
UFC1
1 2 3
Coomassie staining
E
Time[min] 0 15 30 0 15 30 0 15 30 0 15 30
UFC1~UFM1
Lysine 50 mM
Coomassie staining
F
MRE11A
TRIP4
UFL1/UFBP1
MRE11A-UFM1
IB:MRE11A
IB:CDK5RAP3
UFL1-UFM1n
IB:UFL1
GST-TRIP4-UFM1n
IB:TRIP4
1 2 3 4 5

Figure EV5.
