# Supplementary figures and images for: A non‐canonical scaffold‐type E3 ligase complex mediates protein UFMylation
Source: EMBO J. 2022 Sep 19;41(21):e111015. doi: 10.15252/embj.2022111015 (PMC9627666; doi:10.15252/embj.2022111015)

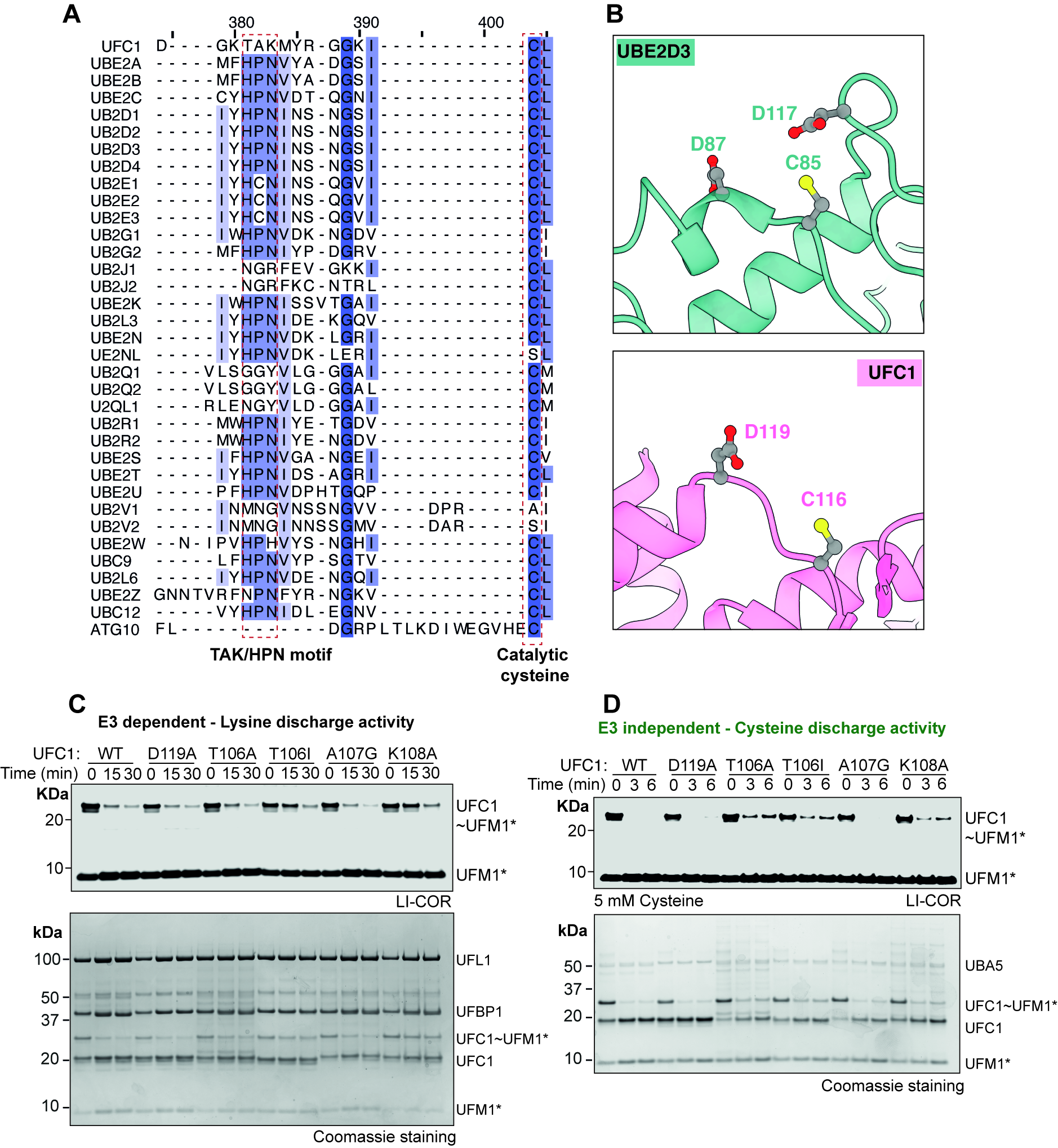

Supplement: Supplementary file 3 — Source Data for Figure 1 [file EMBJ-41-e111015-s004.tif]

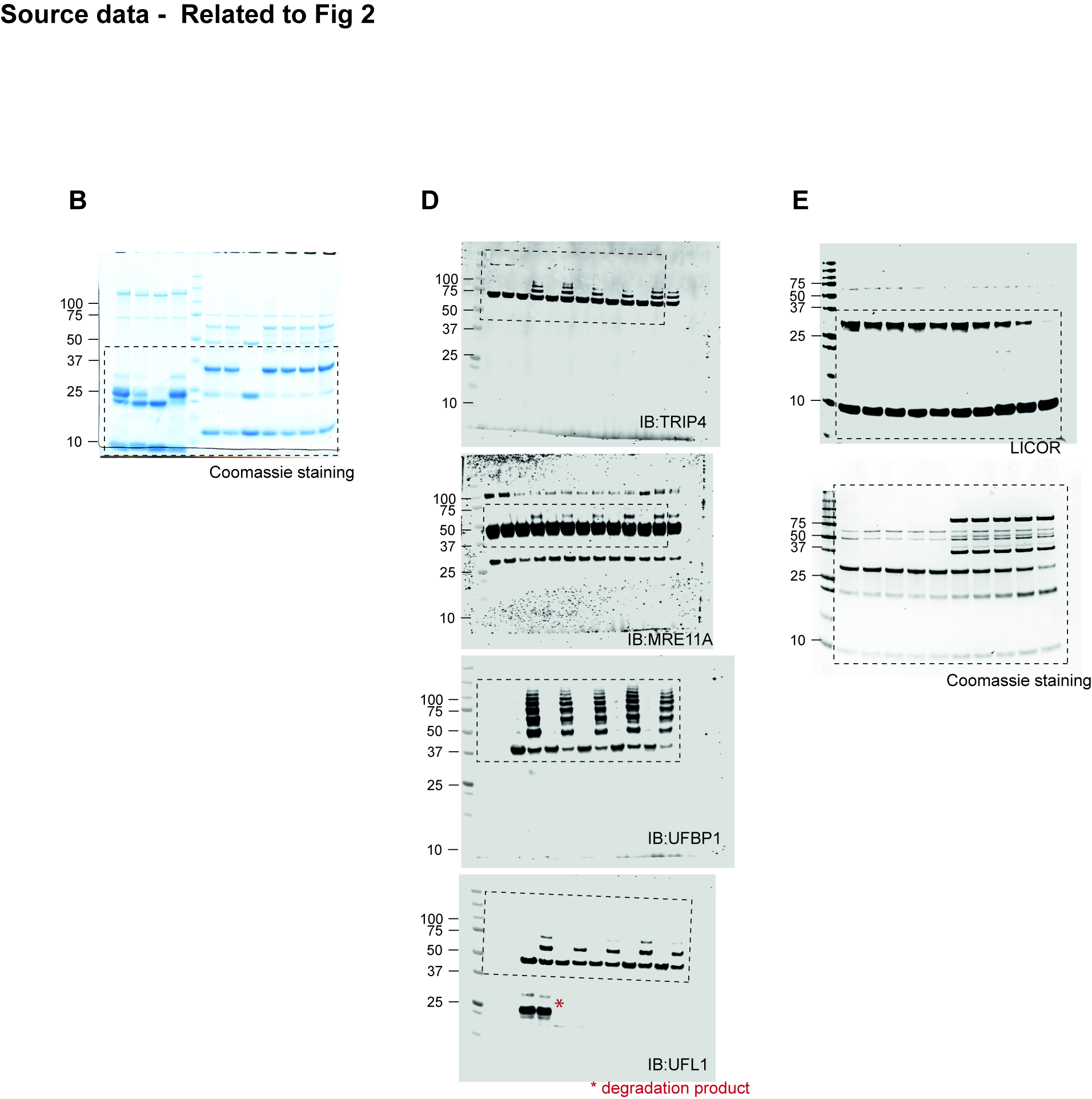

Supplement: Supplementary file 4 — Source Data for Figure 2 [file EMBJ-41-e111015-s002.tif]

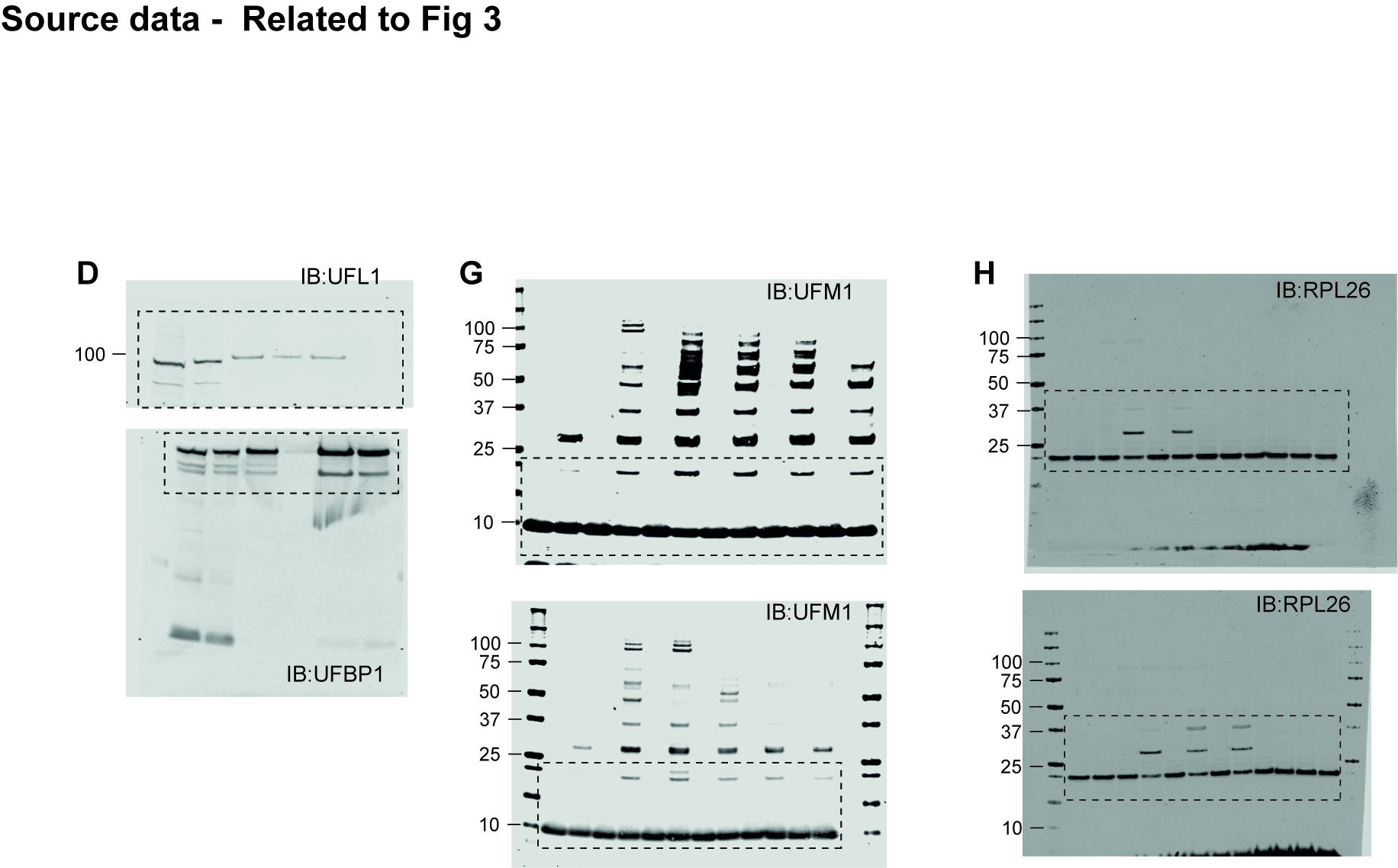

Supplement: Supplementary file 5 — Source Data for Figure 3 [file EMBJ-41-e111015-s005.tif]

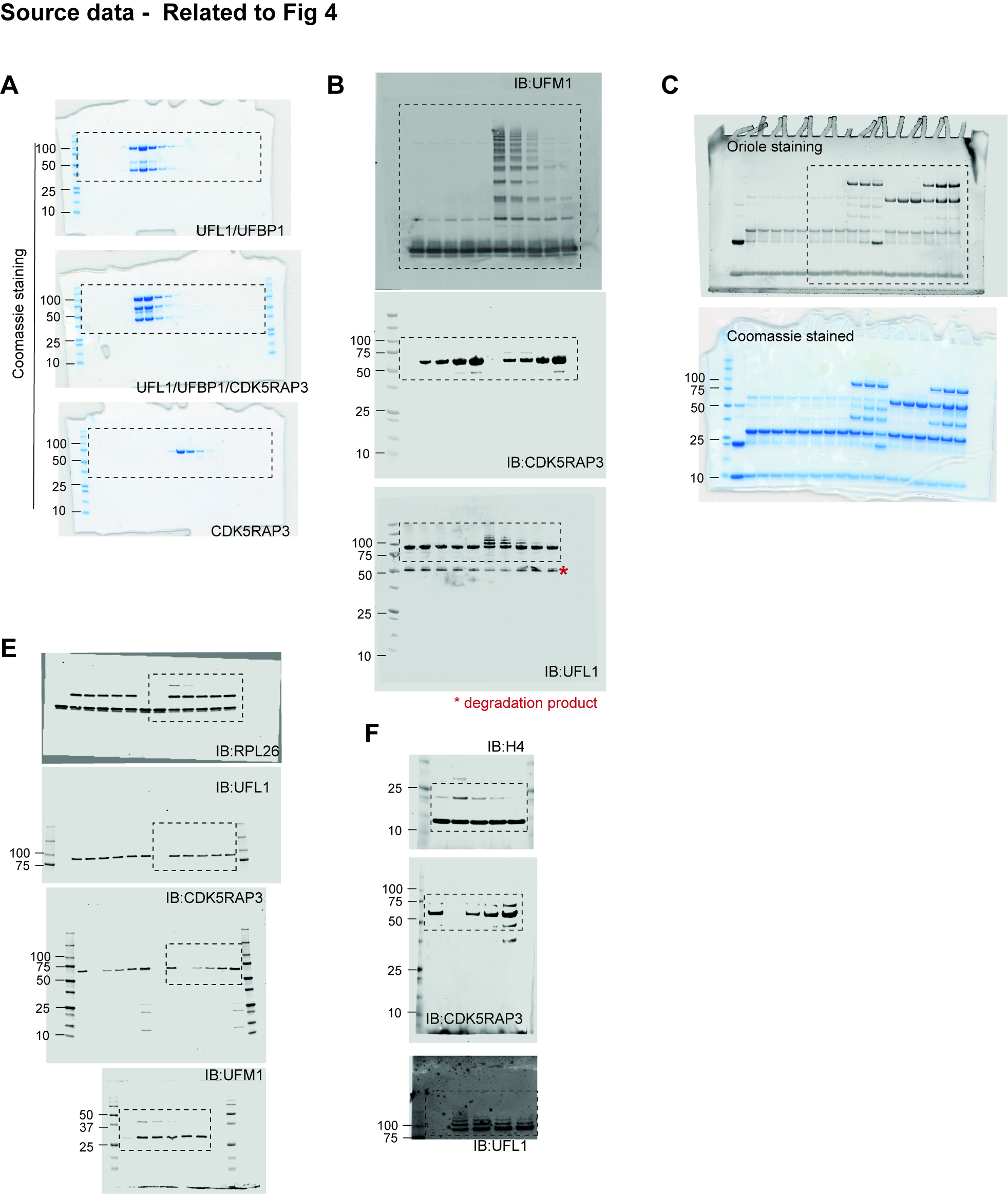

Supplement: Supplementary file 6 — Source Data for Figure 4 [file EMBJ-41-e111015-s003.tif]

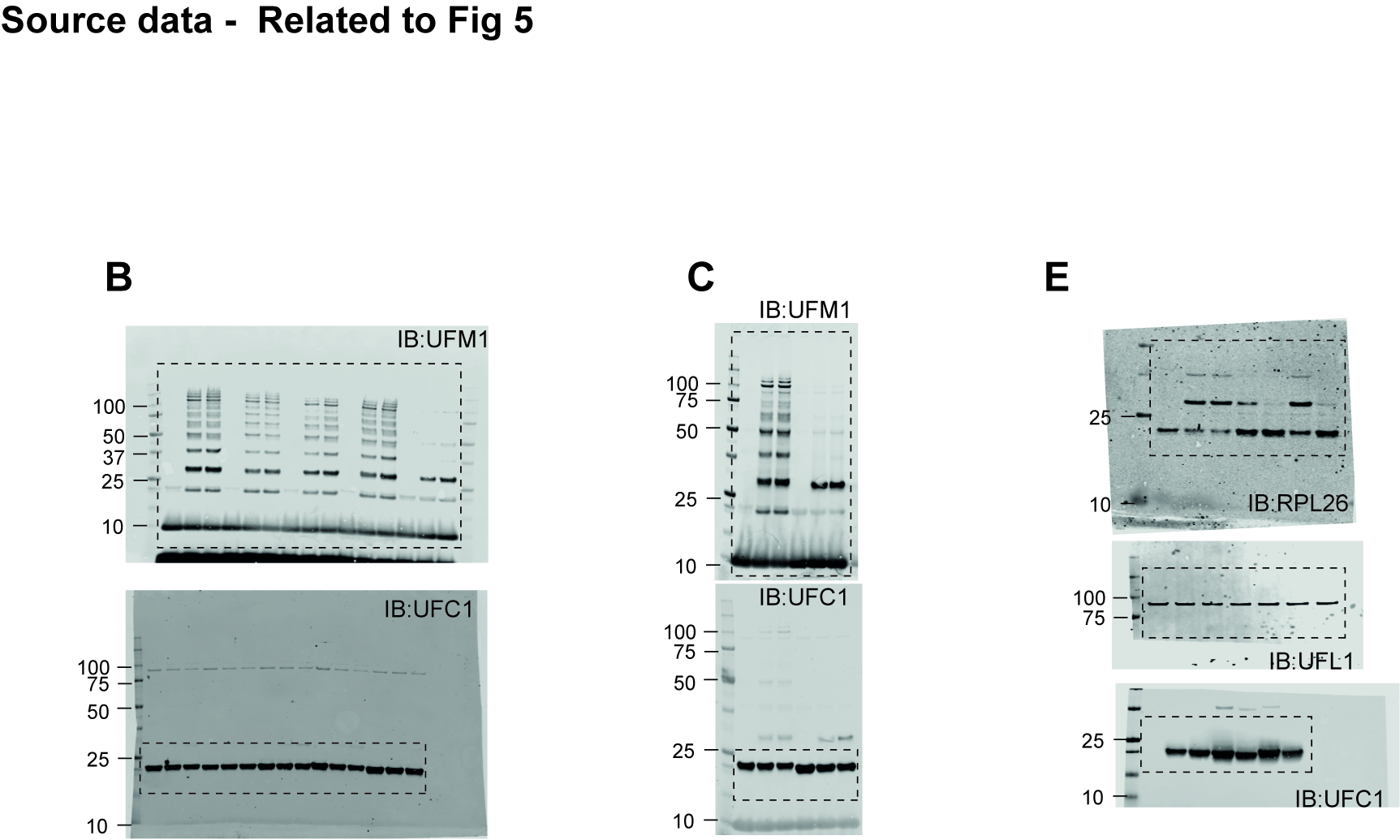

Supplement: Supplementary file 7 — Source Data for Figure 5 [file EMBJ-41-e111015-s009.tif]

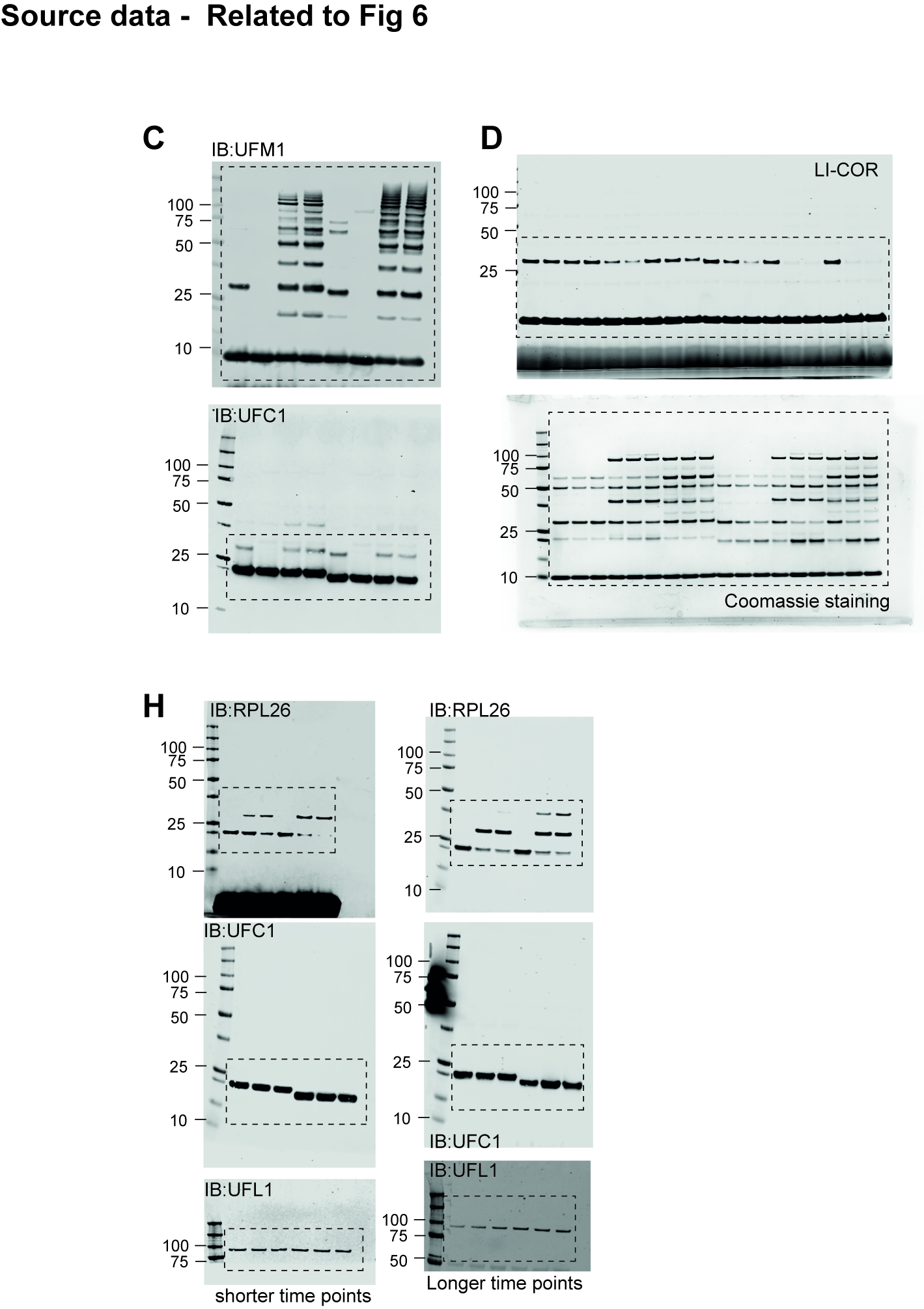

Supplement: Supplementary file 8 — Source Data for Figure 6 [file EMBJ-41-e111015-s008.tif]

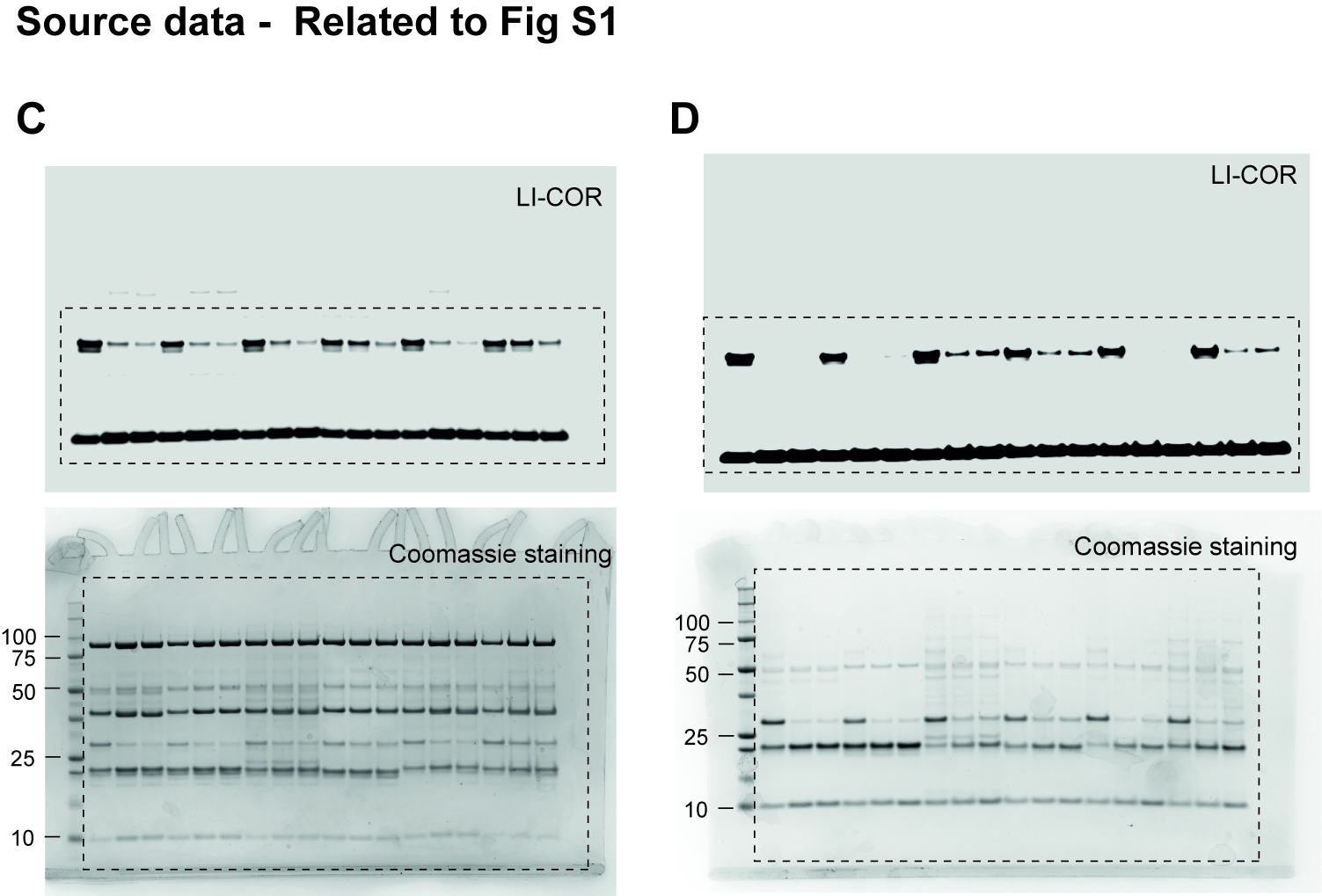

Supplement: Supplementary file 9 — Source Data for Expanded View and Appendix [file EMBJ-41-e111015-s007.zip › EMBOJ-2022-111015R1-FigureS1_Source_Data-sd.tif]

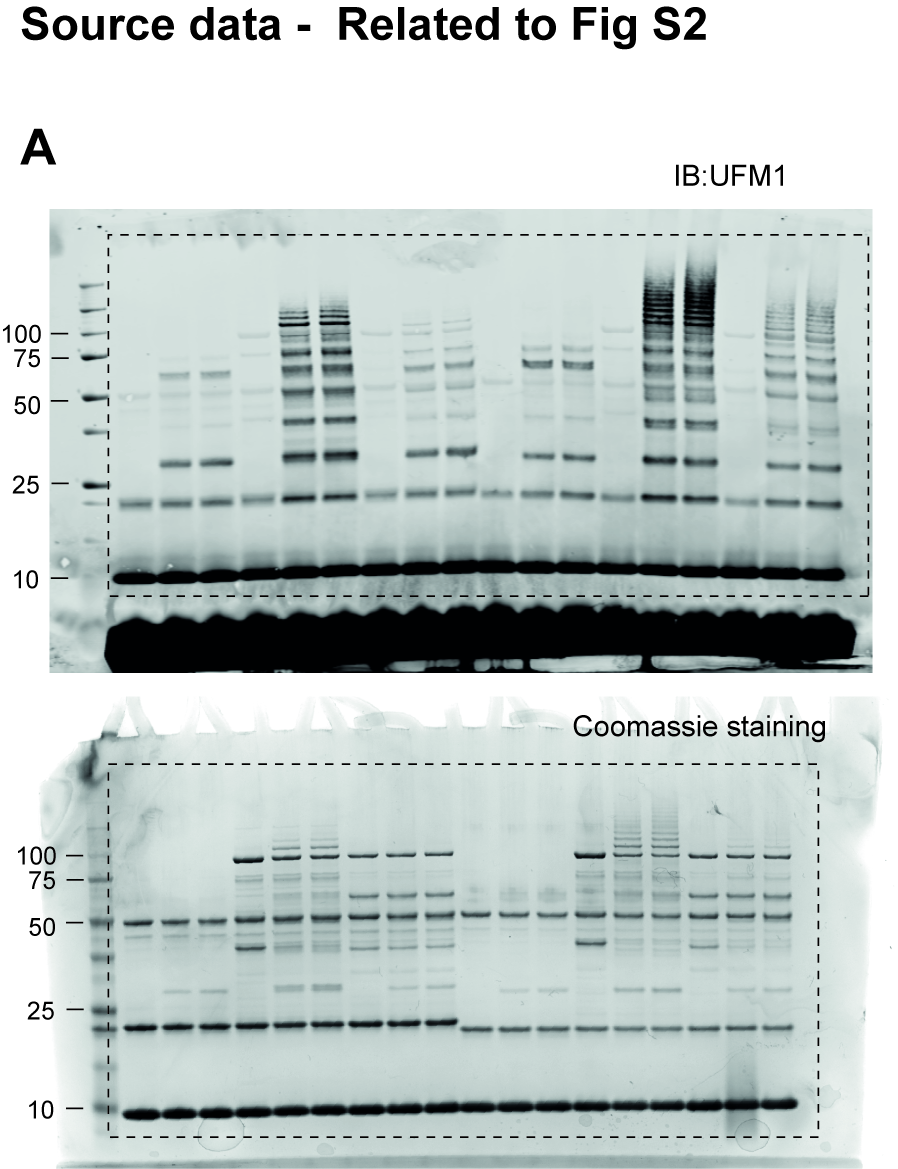

Supplement: Supplementary file 9 — Source Data for Expanded View and Appendix [file EMBJ-41-e111015-s007.zip › EMBOJ-2022-111015R1-FigureS2_Source_Data-sd.tif]

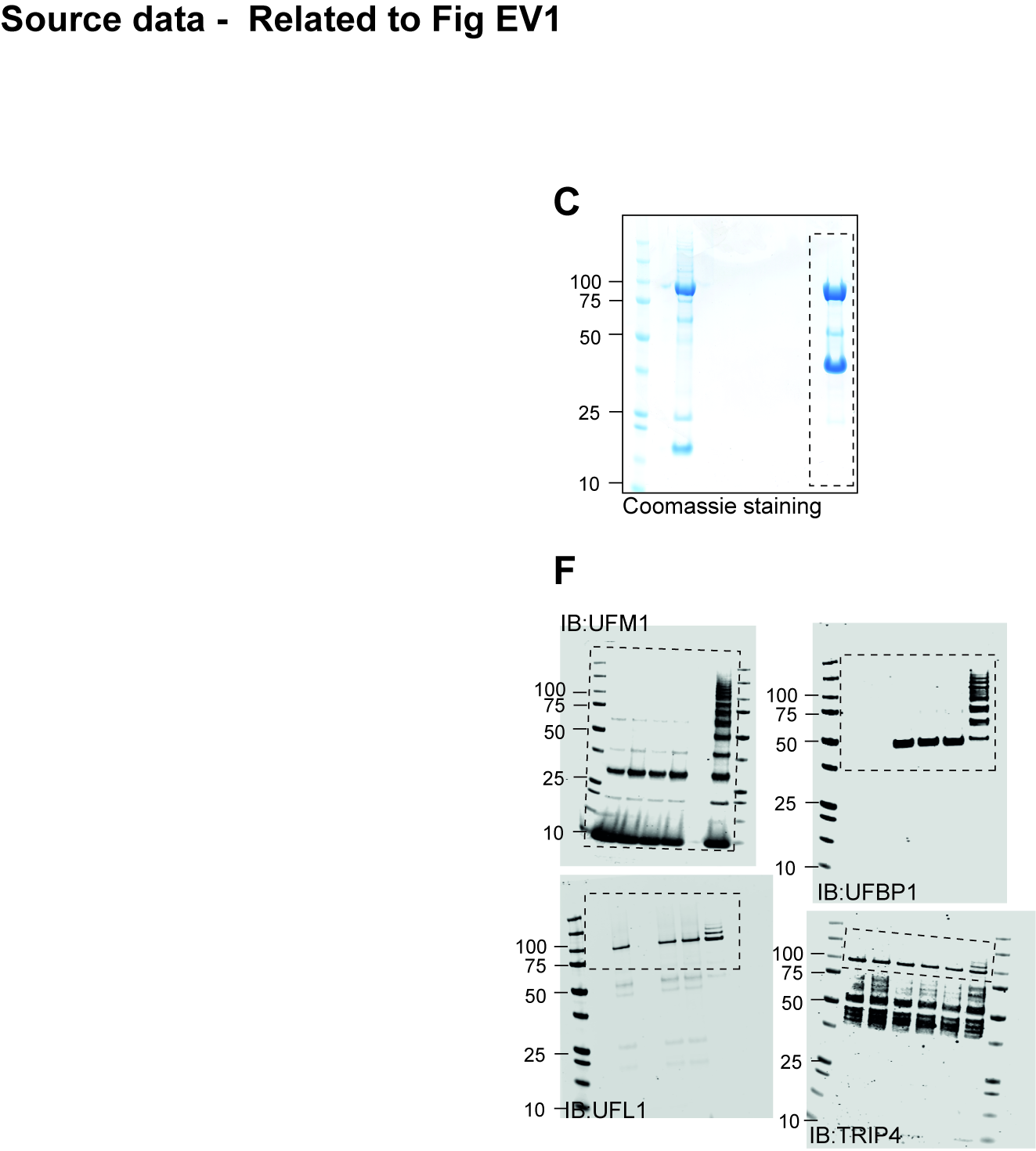

Supplement: Supplementary file 9 — Source Data for Expanded View and Appendix [file EMBJ-41-e111015-s007.zip › EMBOJ-2022-111015R1-FigureEV1_Source_Data-sd.tif]

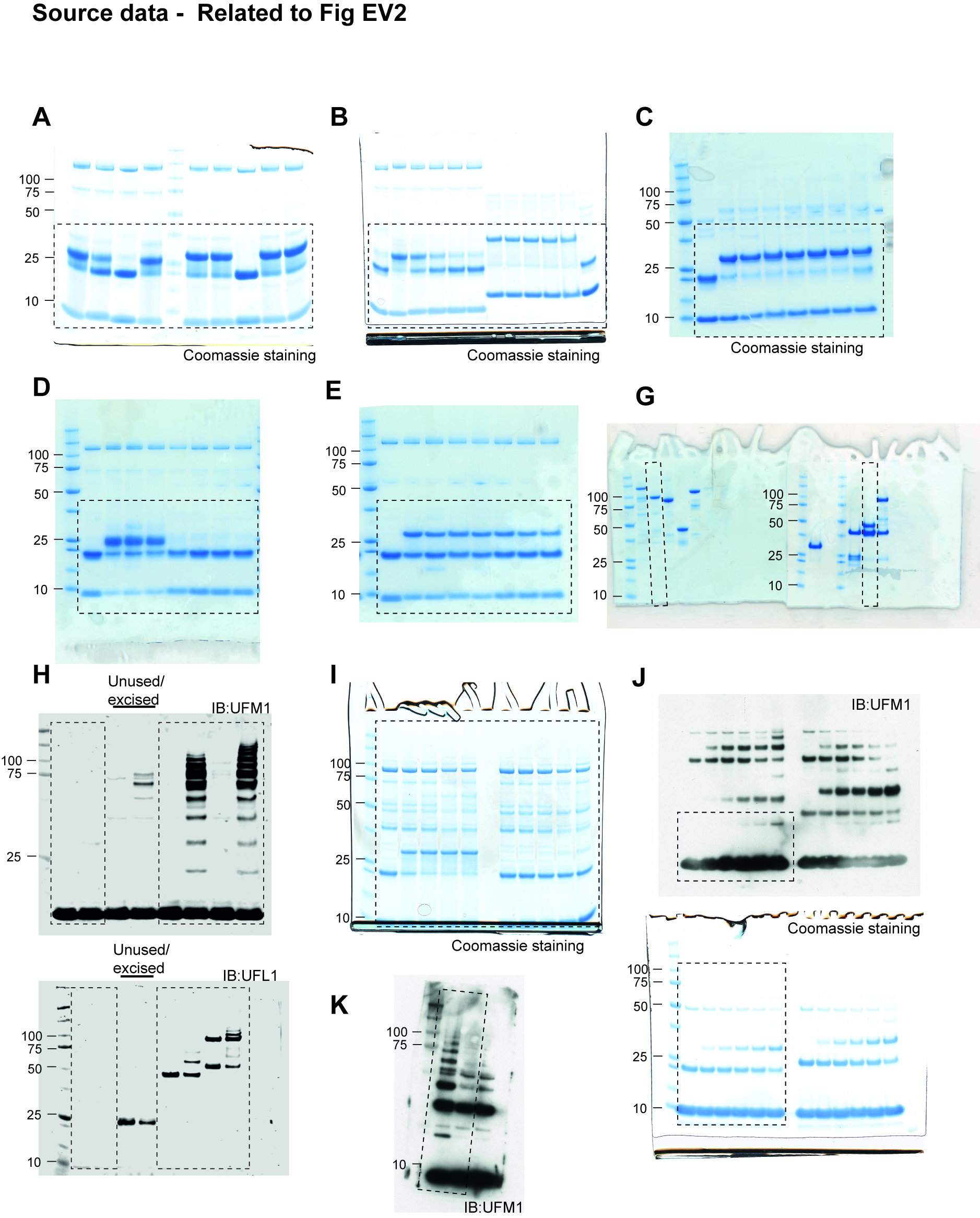

Supplement: Supplementary file 9 — Source Data for Expanded View and Appendix [file EMBJ-41-e111015-s007.zip › EMBOJ-2022-111015R1-FigureEV2_Source_Data-sd.tif]

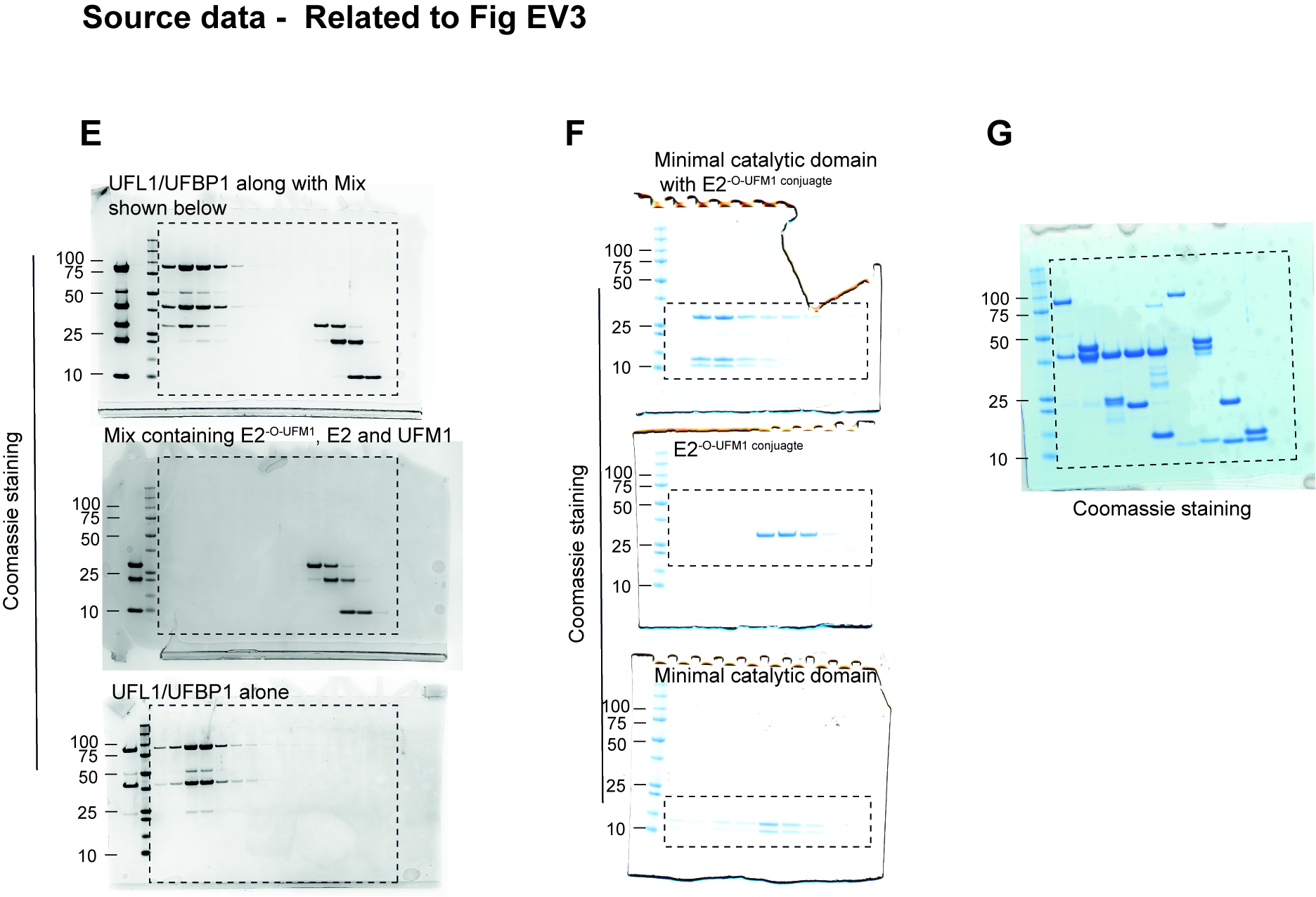

Supplement: Supplementary file 9 — Source Data for Expanded View and Appendix [file EMBJ-41-e111015-s007.zip › EMBOJ-2022-111015R1-FigureEV3_Source_Data-sd.tif]

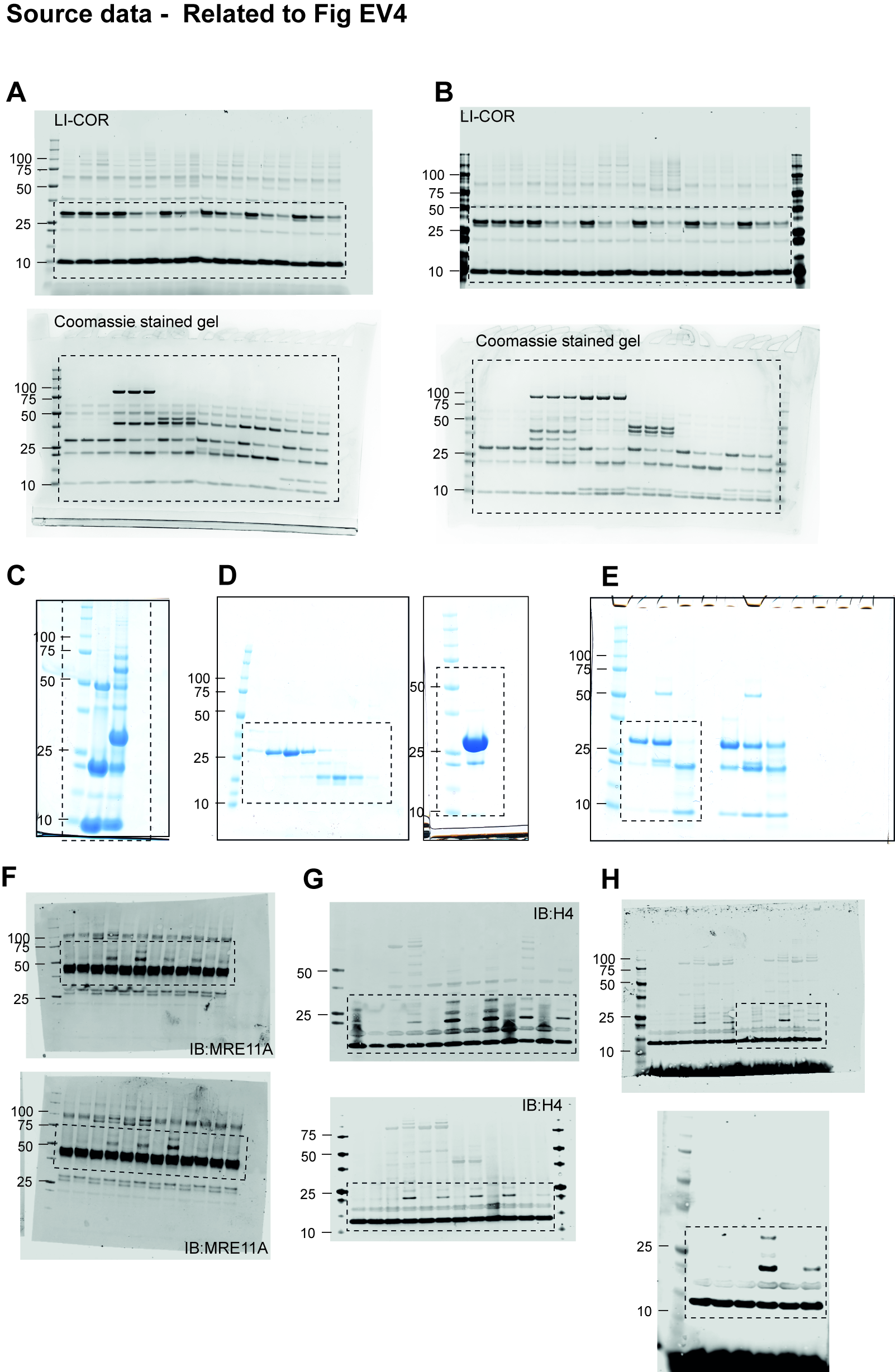

Supplement: Supplementary file 9 — Source Data for Expanded View and Appendix [file EMBJ-41-e111015-s007.zip › EMBOJ-2022-111015R1-FigureEV4_Source_Data-sd.tif]

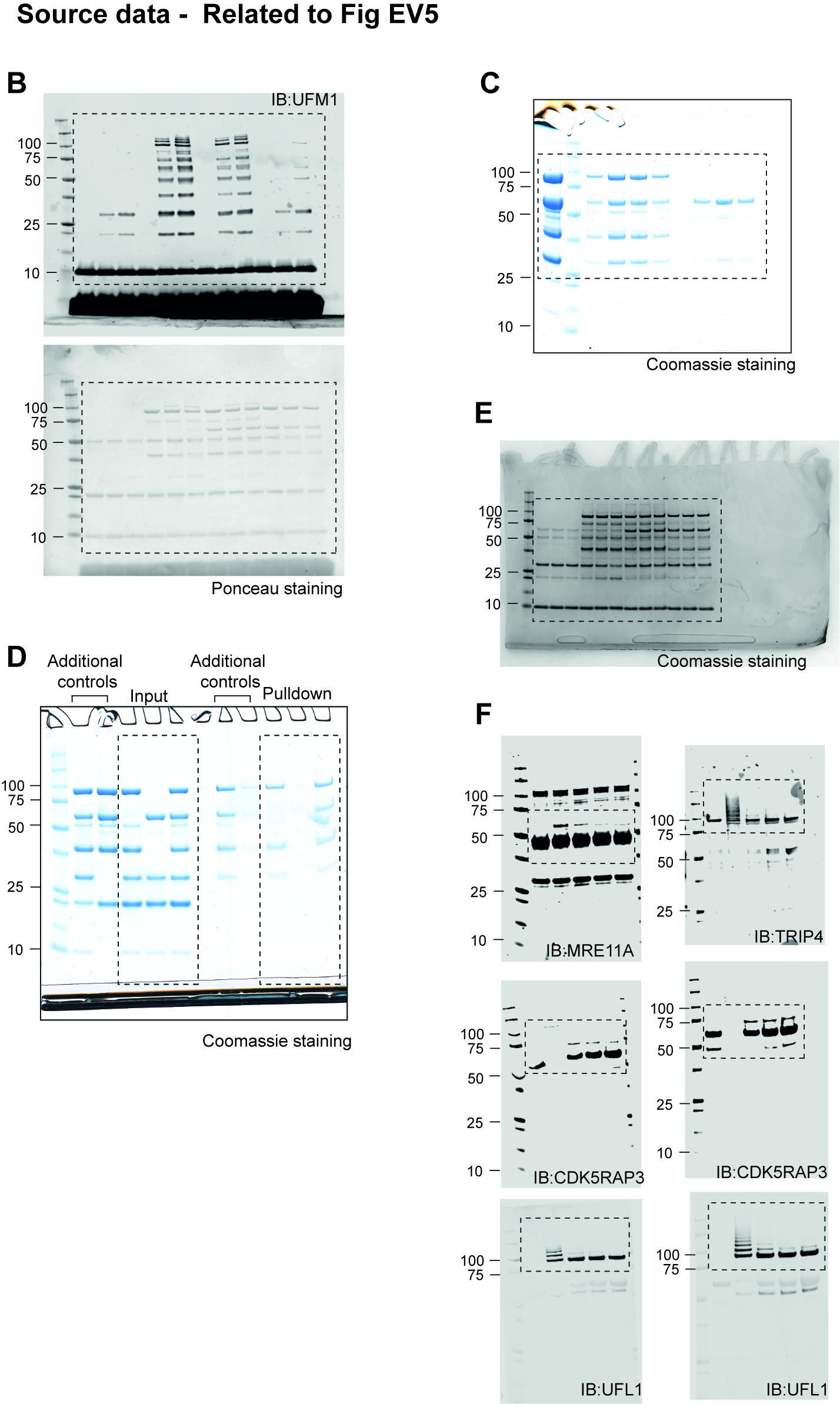

Supplement: Supplementary file 9 — Source Data for Expanded View and Appendix [file EMBJ-41-e111015-s007.zip › EMBOJ-2022-111015R1-FigureEV5_Source_Data-sd.tif]
