# Supplementary material for: A non‐canonical scaffold‐type E3 ligase complex mediates protein UFMylation
Source: EMBO J. 2022 Sep 19;41(21):e111015. doi: 10.15252/embj.2022111015 (PMC9627666; doi:10.15252/embj.2022111015)
Supplement: Supplementary file 11 [file EMBJ-41-e111015-s010.pdf]

THE EMBO JOURNAL

A non-canonical scaffold-type E3 ligase complex mediates protein UFMylation

Joshua Peter, Helge Magnussen, Paul DaRosa, David Millrine, Stephen Matthews, Frederic Lamoliatte, Ramasubramanian Sundaramoorthy, Ron R. Kopito, and Yogesh Kulathu
DOI: 10.15252/embj.2022111015

Corresponding author(s): Yogesh Kulathu (y.kulathu@dundee.ac.uk)

Review Timeline:

Submission Date:	24th Feb 22
Editorial Decision:	15th Mar 22
Revision Received:	12th Jul 22
Editorial Decision:	22nd Aug 22
Revision Received:	25th Aug 22
Accepted:	29th Aug 22

Editor: Hartmut Vodermaier

Transaction Report:

(Note: With the exception of the correction of typographical or spelling errors that could be a source of ambiguity, letters and reports are not edited. Depending on transfer agreements, referee reports obtained elsewhere may or may not be included in this compilation. Referee reports are anonymous unless the Referee chooses to sign their reports.)

1st Editorial Decision 15th Mar 2022

Thank you for submitting your manuscript on reconstitution of the UFMylation machinery to The EMBO Journal. I had sent it to three expert referees, who have now returned the reports copied below. As you will see, the referees acknowledge the technical quality as well as the potential importance of this work, but at the same time also raise a number of concerns that would need to be addressed prior to publication. In this respect, while I would not see it warranted to extend this work towards in vivo studies of processes like ER stress or RQC, as requested by referee 2, it will be important to mutationally validate the AlphaFold-based structural predictions in biochemical, and ideally also some cellular, assays. Moreover, addressing the various specific queries of both referees 1 and 3 and improving presentational issues (as recommended by referee 3) should in my view greatly strengthen the overall impact and wider significance of the study. When revising presentation, please also capitalize on the extended format of an EMBO Journal article (with 7-9 main figures, and up to 5 Expanded View figures that would also be typeset and directly available in the HTML version), in order to make the article as reader-friendly as possible.

Referee #1:

In this manuscript, Peter et al. have developed an in vitro reconstituted UFMylation system and perform an extensive biochemical analysis probing how the UFM E2 conjugating enzyme and E3 ligase function together to UFMylate target proteins. The authors find that the active UFM E3 is a scaffold comprising an obligate heterodimer of UFL1 and UFBP1 and that the adaptor protein CDK5RAP3 attenuates the ligase activity of the E3. The authors also nicely apply AlphaFold-Multimer to guide experiments that identified the minimal fragment of the UFL1/UFBP1 complex harboring E3 ligase activity and show that the E3 activates the lysine reactivity of the E2. Despite its increasingly apparent biological importance, UFMylation has been relatively understudied, particularly at the level of E2 and E3 mechanisms. Though some aspects border on incremental, this rigorous and scholarly study will provide a solid platform for future investigations into this system and should be of interest to a broad readership. For these reasons, this reviewer supports publication of this manuscript in EMBO should the following minor issues be addressed:

* Lines 353-355. Is an alternative interpretation not that CDK5RAP3 specifically interferes with UFM chains being extended on the substrate?

* Did the authors attempt to crystallize and/or perform AlphaFold-Multimer analysis of the minimal E3 in complex with UFC1-UFM1? Same question for CDK5RAP3 interaction with E3/E2. Some of the complexes seem tractable by cryo-EM, has this been attempted?

* Fig 4H- Do the 'B' and 'C' variants of the UFBP1/UFL1 complex harboring C-terminal truncations of Ufl1 exhibit enhanced activity relative to WT complex? If so, what is the basis for this observation?

* The lack of conservation of the HPN motif in UFC1 begs the question of whether the unique TAK motif plays some role in

negative regulation by the N-terminal helix or whether it is indeed important at all. Additionally, whether TAK motif plays some role in patterns of activity observed for UFC1 is worth investigating. Does the identity of these residues play a role in cysteine selectivity in the absence of E3, in the enhanced lysine reactivity in the presence of E3, or the negative regulation by CDK5RAP3 etc.? Additionally, what is the identity of residues known to play a key role in lysine reactivity of ubiquitin E2s (e.g. UbcH5 D87/D117) and how does this factor into the observed data?

Referee #2:

In this study, the authors successfully reconstituted in vitro ufmylation assay with recombinant E3-ligase complex UFL1-UFBP1. They found mainly that the UFL1-UFBP1 complex serves as a scaffold type E3 ligase and that a UFL1-binnding protein CDK5RAP3 has an inhibitory effect on in vitro ufmylation, which advances our understanding of the working principle of Ufmylation reaction. While beautiful in vitro biochemical data and nice predicted structural models by Alphafold2 (AF2), the authors failed to provide in vivo data, and predicted structural data by AF2 are not actually validated. Most problematic issue lack of the physiological significance of Ufmylation in ER-stress, Ribosomal-associated quality control and ER-phagy, the study will be strengthened. While the authors showed the predicted structure of UFL1-UFBP1 complex, they did not construct UFL1 and UFBP1 mutants which show defective interaction each other and verify the effect(s) on Ufmylation assay in cells. They claimed K69-polyUfmylation, but such poluUfmylation had not been observed even in cells lacking UFSP2, a deUfmylating enzyme.

Referee #3:

The manuscript by Peter et al (from the laboratory of Yogesh Kulathu) focuses on deciphering the core elements required for transfer of UFM1 (ubiquitin-fold modifier 1) onto substrates. They extend previous work to define an E3 ligase complex, which comprises both UFL1 and UFBP1, with both proteins appearing to be important for activity. They then take a reductionist approach to identify the critical domains required for activity, including building a model of the E3 complex. Lastly, they go on to identify proteins/elements that appear to regulate transfer of UFM1. The manuscript contains considerable data and will be of interest to those in the field, but in other ways it is quite diffuse with a number of unanswered questions remaining. Some reorganisation of the material may help.

Main points:

1) Figure 1 leads the author to conclude that UFC1 is a Cys reactive E2, with quite compelling data presented. Meanwhile Figure 3 indicates that in the presence of the E3 aminolysis occurs, with no requirement for transfer to cysteines. As a result the authors conclude that the identified E3 acts as a scaffold, with transfer of UFM1 directly from UFC1 to a lysine on the substrate. Further discussion is required to reconcile these observations.
2) Related to the above point, the authors should consider reorganising their manuscript, with potentially Figure 2 and related material as the initial focus?
3) Considerable emphasis is placed on the Alphafold model presented in Figure 4A&4B. The authors may wish to discuss limitations of the model and should consider ways to build confidence in this model of the complex - do the expected parts of the two proteins cross-link to each other? Does the conformation of the NTR seem likely, or would it be more likely to pack against other elements/proteins?
4) The inhibition afforded by interaction of UFL1/UFBP1 with CDK5RAP3 is impressive, but it is difficult to reconcile its ability to 'prevent activation of UFC1~UFM1' (page 12, line 340) but not block mono UFMylation of the substrate, RPL26. The mechanism by which this is achieved is difficult to conceive. The authors then go on to suggest that the N-terminus of UFC1 is important for inhibition by CDK5RAP5, but again it is hard to reconcile this with the observations seen in Figure 5.
5) Parts of the discussion are good and help place the results reported in context, but other sections appear somewhat speculative and I suspect difficult for the general reader to decipher. Effort to better distinguish what is shown and what is proposed/possible may help.

Minor points:

1) The abstract needs some revision, currently it does not entice the general reader.
2) Page 3, line 47 - sentence should be reordered.
3) Page 3, line 69 - the role of the backside of UbcH5b should be revised taking into account the work of Danny Huang (especially Buetow et al., 2016 and Patel et al., 2019) that show UbB is an allosteric activator of the E2. The reference to dimerization is misleading.
4) Legend S1, needs lysine added after 150 mM
5) Figure 2A/2B, the apparent differences in the sizes of the complex should be discussed here - 2A suggests a higher order oligomer.
6) Figure labelling is a bit confusing - e.g. Figure 2D it might be better to indicate in each lane what is present - as currently presented the misaligned dots are not immediately clear.
7) Figure 3A, the breaks in the sequence should be clearer. It would also be helpful to include a domain diagram of UFL1 so the relative position of the Cys residues is more obvious to the reader.
8) Figure 3C, the reaction appears complete at 15 minutes, shorter time points might be helpful.
9) Figure 4C is confusing - do the authors intend to indicate fusion proteins? If not, it would be better to separate the two proteins

in the schematic.
10) Figure 4D, a representative gel should be included for these assays or is this data from Figure S5? If so it should be stated in the legend. Also in this case it may be better to show all the data points rather than error bars (also applies to 5E and 6E-6G).
11) Figure 4E is hard to interpret, at the very least a trace of the UFL1/UFBP1complex run under the same conditions is required. Co-elution of the conjugate with the E3 complex is not very convincing.
12) Figure 5E legend should refer to the gel shown in panel D (not E).
13) Page 10, line 278 'for which we performed' could be replaced with 'using' but this sentence and the next could be rewritten and simplified.
14) In many gels lane numbers are included but they are rarely referred to in the text. In some figures e.g. Figure 6D it may be helpful to point to specific lanes which should be compared.
The manuscript suffers from a number of very similar abbreviations and while this is a consequence of the field the authors could do more to help the reader. For example, nowhere is UFM1 defined, while referring to UFC1~UFM1 as a conjugate might help the reader recall that this is equivalent to a E2~Ub conjugate. In other places the clarity of the text could be improved by spelling things out a little more.

Response to Reviewers' comments:

We would like to thank the reviewers for their interest in our work and for their constructive comments and suggestions, which have allowed us to strengthen our findings and improve the manuscript. A point-by-point response addressing each of their comments is detailed below.

Referee #1:
In this manuscript, Peter et al. have developed an in vitro reconstituted UFMylation system and perform an extensive biochemical analysis probing how the UFM E2 conjugating enzyme and E3 ligase function together to UFMylate target proteins. The authors find that the active UFM E3 is a scaffold comprising an obligate heterodimer of UFL1 and UFBP1 and that the adaptor protein CDK5RAP3 attenuates the ligase activity of the E3. The authors also nicely apply AlphaFold-Multimer to guide experiments that identified the minimal fragment of the UFL1/UFBP1 complex harboring E3 ligase activity and show that the E3 activates the lysine reactivity of the E2. Despite its increasingly apparent biological importance, UFMylation has been relatively understudied, particularly at the level of E2 and E3 mechanisms. Though some aspects border on incremental, this rigorous and scholarly study will provide a solid platform for future investigations into this system and should be of interest to a broad readership. For these reasons, this reviewer supports publication of this manuscript in EMBO should the following minor issues be addressed:
We thank the reviewer for their positive comments about our study.

* Lines 353-355. Is an alternative interpretation not that CDK5RAP3 specifically interferes with UFM chains being extended on the substrate?
We postulate that CDK5RAP3 is a substrate adaptor directing UFMylation to the ribosomal subunit RPL26. In this manner, it prevents UFM1 from being attached to another UFM1 molecule thereby restricting UFM1 chain formation on substrates. We have included this alternative interpretation in Discussion.

*Did the authors attempt to crystallize and/or perform AlphaFold-Multimer analysis of the minimal E3 in complex with UFC1-UFM1? Same question for CDK5RAP3 interaction with E3/E2. Some of the complexes seem tractable by cryo-EM, has this been attempted?
We thank the reviewer for the suggestion. Indeed, we tried crystallization and also cryo-EM. Unfortunately, despite extensive efforts with both approaches we failed to obtain a structure. We are actively working on this and hope to obtain a structure that reveals the molecular mechanism of this atypical ligase complex.

*Fig 4H- Do the 'B' and 'C' variants of the UFBP1/UFL1 complex harboring C-terminal truncations of Ufl1 exhibit enhanced activity relative to WT complex? If so, what is the basis for this observation?
To simplify these observations, we have now compared all the truncated variants with 60S ribosome as the substrate. The reason for choosing 60S as the substrate is that UFMylation of RPL26 occurs efficiently with ~50% modification within 5 minutes at 30°C whereas other substrates such as Histone H4, MRE11A and TRIP4 require 1 h incubation at 37°C to result in modification of only a small fraction of the substrate.

* The lack of conservation of the HPN motif in UFC1 begs the question of whether the unique TAK motif plays some role in negative regulation by the N-terminal helix or whether it is indeed important at all. Additionally, whether TAK motif plays some role in patterns of activity observed for UFC1 is worth investigating. Does the identity of these residues play a role in cysteine selectivity in the absence of E3, in the enhanced lysine reactivity in the presence of E3, or the negative regulation by CDK5RAP3 etc.? Additionally, what is the

identity of residues known to play a key role in lysine reactivity of ubiquitin E2s (e.g. UbcH5 D87/D117) and how does this factor into the observed data?

We thank the reviewer for these suggestions and have now performed a more detailed analysis of the TAK motif and the N-terminal helix which has revealed interesting insights into UFC1. With these new results, we have created new figures (see Figure 5, Fig S1 and Fig 6) to include these findings.

Referee #2:
In this study, the authors successfully reconstituted in vitro ufmylation assay with recombinant E3-ligase complex UFL1-UFBP1. They found mainly that the UFL1-UFBP1 complex serves as a scaffold type E3 ligase and that a UFL1-binnding protein CDK5RAP3 has an inhibitory effect on in vitro ufmylation, which advances our understanding of the working principle of Ufmylation reaction. While beautiful in vitro biochemical data and nice predicted structural models by Alphafold2 (AF2), the authors failed to provide in vivo data, and predicted structural data by AF2 are not actually validated. Most problematic issue lack of the physiological significance of Ufmylation in ER-stress, Ribosomal-associated quality control and ER-phagy, the study will be strengthened. While the authors showed the predicted structure of UFL1-UFBP1 complex, they did not construct UFL1 and UFBP1 mutants which show defective interaction each other and verify the effect(s) on Ufmylation assay in cells. They claimed K69-polyUfmylation, but such poluUfmylation had not been observed even in cells lacking UFSP2, a deUfmylating enzyme.
We thank the reviewer for their comments. The main focus of this work was to define the minimal requirements of UFMylation and provide a characterization of the molecular players involved. Examining the role of UFMylation in ER stress, RQC and ERphagy, we feel are therefore outside the scope of this present manuscript.

We agree that it is important to validate the predicted structure of the UFL1-UFBP1 complex. We have now analysed the predicted structure and engineered mutations that would disrupt the interaction between the two proteins. By reconstituting UFL1 knockout cells with wild type and mutant UFL1, we can nicely show that the association between UFL1-UFBP1 can be disrupted by a single point mutation in the composite WH domain predicted by AlphaFold. This result is now included in the revised manuscript and supports the predicted structure of the complex.

As for K69-linked UFMylation our work clearly demonstrates that K69-linked polyUFM1 chains are assembled in vitro. We show this both with the use of UFM1 Lys to Arg mutants and also using mass spectrometry. Previous work (Yoo et al. Mol Cell 2014) have found ASC1/TRIP4 to be modified with K69-linked UFM1 chains in HEK293 cells.

Referee #3:

The manuscript by Peter et al (from the laboratory of Yogesh Kulathu) focuses on deciphering the core elements required for transfer of UFM1 (ubiquitin-fold modifier 1) onto substrates. They extend previous work to define an E3 ligase complex, which comprises both UFL1 and UFBP1, with both proteins appearing to be important for activity. They then take a reductionist approach to identify the critical domains required for activity, including building a model of the E3 complex. Lastly, they go on to identify proteins/elements that appear to regulate transfer of UFM1. The manuscript contains considerable data and will be of interest to those in the field, but in other ways it is quite diffuse with a number of unanswered questions remaining. Some reorganisation of the material may help.

We thank the reviewer for their positive evaluation of our work and helpful suggestions which we have incorporated into the revised manuscript

Main points:

1) Figure 1 leads the author to conclude that UFC1 is a Cys reactive E2, with quite compelling data presented. Meanwhile Figure 3 indicates that in the presence of the E3 aminolysis occurs, with no requirement for transfer to cysteines. As a result the authors conclude that the identified E3 acts as a scaffold, with transfer of UFM1 directly from UFC1 to a lysine on the substrate. Further discussion is required to reconcile these observations.
2) Related to the above point, the authors should consider reorganising their manuscript, with potentially Figure 2 and related material as the initial focus?
We thank the reviewer for these suggestions and have reorganized the manuscript by presenting the results in Figure 2 first and merging Figure 1 (old) with Figure 3. We have also restructured the figures to make sure that these points regarding UFL1/UFBP1 being a scaffold type ligase complex come across properly.

3) Considerable emphasis is placed on the Alphafold model presented in Figure 4A&4B. The authors may wish to discuss limitations of the model and should consider ways to build confidence in this model of the complex - do the expected parts of the two proteins cross-link to each other? Does the conformation of the NTR seem likely, or would it be more likely to pack against other elements/proteins?
We have now added a sentence to discuss limitations of the model and the possibility that the complex adopts a different conformation when bound to substrates such as the ribosome. Additionally, we have made point mutations at the interface between UFL1 and UFBP1 to disrupt complex formation, which provides some confidence to the Alphafold prediction of how UFL1 binds to UFBP1.

4) The inhibition afforded by interaction of UFL1/UFBP1 with CDK5RAP3 is impressive, but it is difficult to reconcile its ability to 'prevent activation of UFC1~UFM1' (page 12, line 340) but not block mono UFMylation of the substrate, RPL26. The mechanism by which this is achieved is difficult to conceive. The authors then go on to suggest that the N-terminus of UFC1 is important for inhibition by CDK5RAP5, but again it is hard to reconcile this with the observations seen in Figure 5.
Our results suggest that CDK5RAP3 binds to UFL1/UFBP1, UFC1~UFM1 in such a way that it inhibits the complex preventing discharge of UFM1 or activation. In the presence of appropriate substrate such as the ribosome, this autoinhibition is relieved and UFM1 is discharged onto RPL26. We postulate that CDK5RAP3 regulates E3 ligase activity and is a substrate adaptor directing UFMylation to the ribosome subunit RPL26. In this manner, it prevents UFM1 from being attached to another UFM1 molecule thereby restricting UFM1 chain formation on substrates. A full understanding of the precise mechanism will require structural biology efforts which we are currently pursuing.

5) Parts of the discussion are good and help place the results reported in context, but other sections appear somewhat speculative and I suspect difficult for the general reader to decipher. Effort to better distinguish what is shown and what is proposed/possible may help.
We thank the reviewer for pointing this out and have carefully gone through the discussion and made these changes.

Minor points:

1) The abstract needs some revision, currently it does not entice the general reader.
Done

2) Page 3, line 47 - sentence should be reordered.
Done

3) Page 3, line 69 - the role of the backside of UbcH5b should be revised taking into account the work of Danny Huang (especially Buetow et al., 2016 and Patel et al., 2019) that show UbB is an allosteric activator of the E2. The reference to dimerization is misleading.
Done

4) Legend S1, needs lysine added after 150 mM
Done

5) Figure 2A/2B, the apparent differences in the sizes of the complex should be discussed here - 2A suggests a higher order oligomer.

We speculate that the shape of UFL1/UFBP1 complex could explain its behaviour in Size exclusion chromatography (see Fig 3A). This prompted us to cross-check the actual molecular weight using other techniques such as Mass photometry (new version:Fig 1B, old version: Fig 2B) and SEC-MALS (new version:Fig S1D, old version:Fig S2D). Using these two techniques, we find the experimental molecular weight of the UFL1/UFBP1 to be closer to 131KDa and 126KDa respectively. (Also discussed in the results section- Page 10 - lines 274, 275)

6) Figure labelling is a bit confusing - e.g. Figure 2D it might be better to indicate in each lane what is present - as currently presented the misaligned dots are not immediately clear.

We have made changes in the figure and included two more dots to indicate the presence and absence of ATP/E3 ligase in the reactions. We have also included the lane numbers in the figure legend.

7) Figure 3A, the breaks in the sequence should be clearer. It would also be helpful to include a domain diagram of UFL1 so the relative position of the Cys residues is more obvious to the reader.
In order to make the breaks visible, we have now placed a thick red line (Please see figure 2C in new version). An issue with the domain diagram is, we don't introduce Alphafold models at this point in the manuscript. And apart from domains that Alphafold predicted, we couldn't predict any domains using primary sequence of UFL1.

8) Figure 3C, the reaction appears complete at 15 minutes, shorter time points might be helpful.
We agree with the reviewer. Therefore, we have repeated this assay and used shorter time points to monitor the discharge (minutes 0, 1, 3, 5,15). One experiment of 3 repeats has been shown in Fig 2E (new version).

9) Figure 4C is confusing - do the authors intend to indicate fusion proteins? If not, it would be better to separate the two proteins in the schematic.
We thank the reviewer for pointing this out and we have now removed the lines connecting the two proteins in the schematic.

10) Figure 4D, a representative gel should be included for these assays or is this data from Figure S5? If so it should be stated in the legend. Also, in this case it may be better to show all the data points rather than error bars (also applies to 5E and 6E-6G).
Done

11) Figure 4E is hard to interpret, at the very least a trace of the UFL1/UFBP1complex run

under the same conditions is required. Co-elution of the conjugate with the E3 complex is not very convincing.
A separate UV trace of UFL1/UFBP1 complex and a Coomassie stained gel showing corresponding fractions is included in the figure now.

12) Figure 5E legend should refer to the gel shown in panel D (not E).
We thank the reviewer for pointing this error. We have now rectified it.

13) Page 10, line 278 'for which we performed' could be replaced with 'using' but this sentence and the next could be rewritten and simplified.
We thank the reviewer for this suggestion. We have now replaced those sentences

14) In many gels lane numbers are included but they are rarely referred to in the text. In some figures e.g. Figure 6D it may be helpful to point to specific lanes which should be compared.
Done

The manuscript suffers from a number of very similar abbreviations and while this is a consequence of the field the authors could do more to help the reader. For example, nowhere is UFM1 defined, while referring to UFC1~UFM1 as a conjugate might help the reader recall that this is equivalent to a E2~Ub conjugate. In other places the clarity of the text could be improved by spelling things out a little more.

We have reorganised the figures and rewritten the manuscript to improve the clarity and flow of the manuscript.

1st Revision - Editorial Decision 22nd Aug 2022

Thank you for submitting your revised manuscript on UFM1 conjugation mechanisms for our consideration. I apologize for the delay in getting back to you with a response, but the re-reviews from two of the original referees arrived while I was away from the office. I am happy to say that the referees are for the most part satisfied with the revisions, and only referee 3 retains a number of minor presentational concerns (copied below) that I would ask you to incorporate into the manuscript text during a final round of minor revision. I would appreciate if you would make those modifications, with "track changes" remaining activated, directly in the attached text file. In the same file, you will see that I made some minor changes to the title and abstract, mainly to make them somewhat more explicit and accessible to a broad readership - please check and choose between the two proposed title alternatives.

Referee #1:

In their revised manuscript, Peter et al. have suitably addressed the key questions and concerns of reviewers and have considerably strengthened the major conclusions of their study. This reviewer supports publication of the revised manuscript in EMBO Journal.

Referee #3:

The revised manuscript is much improved. Reordering of the Results section has made the data more accessible to the audience .

Line 262 - It is important to continue to remind the reader that the proposed structure is a model. For examples i.e. 'the predicted formation of this composite WH domain provides a potential explanation'

In Figure 3A the position of the NTR seems unlikely? Although a sentence about the limitations of the model has been added, this sentence could be better connected to features of the model shown in Figure 3A.

Line 264 - this paragraph still seems to overstate the conclusions given the model structure. It is an attractive model, but the L45R mutant only shows that L45 is important for complex formation, it does not show that it makes the predicted contact. It could simply cause UFL1 to unfold. Prrof of the contact would require additional experiments such crosslinking or charge-swap mutations.

D119 should be highlighted in Figure 5A.

Line 301: complex 1 is not denoted in figure 3E, or in the extended version?

In the last paragraph of the discussion, the importance of extending the studies into a cellular setting should be highlighted, more than just structural work and cell free systems will be needed to understand how UFMylation is regulated in cells.

Response to reviewers:
Referee #1:

In their revised manuscript, Peter et al. have suitably addressed the key questions and concerns of reviewers and have considerably strengthened the major conclusions of their study. This reviewer supports publication of the revised manuscript in EMBO Journal.

Referee #3:

The revised manuscript is much improved. Reordering of the Results section has made the data more accessible to the audience .

Line 262 - It is important to continue to remind the reader that the proposed structure is a model. For examples i.e. 'the predicted formation of this composite WH domain provides a potential explanation'
Done

In Figure 3A the position of the NTR seems unlikely? Although a sentence about the limitations of the model has been added, this sentence could be better connected to features of the model shown in Figure 3A.
Done

Line 264 - this paragraph still seems to overstate the conclusions given the model structure. It is an attractive model, but the L45R mutant only shows that L45 is important for complex formation, it does not show that it makes the predicted contact. It could simply cause UFL1 to unfold. Prrof of the contact would require additional experiments such crosslinking or charge-swap mutations.
We cannot discount the possibility that L45R mutation could cause the protein to unfold. We have now clearly stated in this paragraph and also throughout the manuscript that the Alphafold structures shown are predictions.

D119 should be highlighted in Figure 5A.
This is shown in Figure S1A and is also compared to the equivalent D87 residue in UBE2D3

Line 301: complex 1 is not denoted in figure 3E, or in the extended version?
Fixed

In the last paragraph of the discussion, the importance of extending the studies into a cellular setting should be highlighted, more than just structural work and cell free systems will be needed to understand how UFMylation is regulated in cells.
A sentence to this effect has now been added.

2nd Revision - Editorial Decision 29th Aug 2022

Thank you for submitting your final revised manuscript for our consideration. I am pleased to inform you that we have now accepted it for publication in The EMBO Journal.

EMBO Press Author Checklist

Corresponding Author Name: Yogesh Kulathu	
Journal Submitted to: EMBO Journal	
Manuscript Number: EMBOJ-2022-111015	

USEFUL LINKS FOR COMPLETING THIS FORM
The EMBO Journal - Author Guidelines
EMBO Reports - Author Guidelines
Molecular Systems Biology - Author Guidelines
EMBO Molecular Medicine - Author Guidelines

Reporting Checklist for Life Science Articles (updated January
This checklist is adapted from Materials Design Analysis Reporting (MDAR) Checklist for Authors. MDAR establishes a minimum set of requirements in transparent reporting in the life sciences (see Statement of Task: 10.31222/osf.io/9sm4x). Please follow the journal's guidelines in preparing your
Please note that a copy of this checklist will be published alongside your article.

Abridged guidelines for figures
1. Data
The data shown in figures should satisfy the following conditions:
- the data were obtained and processed according to the field’s best practice and are presented to reflect the results of the experiments in an accurate and unbiased manner.
- ideally, figure panels should include only measurements that are directly comparable to each other and obtained with the same assay.
- plots include clearly labeled error bars for independent experiments and sample sizes. Unless justified, error bars should not be shown for technical
- if n<5, the individual data points from each experiment should be plotted. Any statistical test employed should be justified.
- Source Data should be included to report the data underlying figures according to the guidelines set out in the authorship guidelines on Data

2. Captions
Each figure caption should contain the following information, for each panel where they are relevant:
- a specification of the experimental system investigated (eg cell line, species name).
- the assay(s) and method(s) used to carry out the reported observations and measurements.
- an explicit mention of the biological and chemical entity(ies) that are being measured.
- an explicit mention of the biological and chemical entity(ies) that are altered/varied/perturbed in a controlled manner.
- the exact sample size (n) for each experimental group/condition, given as a number, not a range;
- a description of the sample collection allowing the reader to understand whether the samples represent technical or biological replicates (including how many animals, litters, cultures, etc.).
- a statement of how many times the experiment shown was independently replicated in the laboratory.
- definitions of statistical methods and measures:
  - common tests, such as t-test (please specify whether paired vs. unpaired), simple χ2 tests, Wilcoxon and Mann-Whitney tests, can be unambiguously identified by name only, but more complex techniques should be described in the methods section;
  - are tests one-sided or two-sided?
  - are there adjustments for multiple comparisons?
  - exact statistical test results, e.g., P values = x but not P values < x;
  - definition of ‘center values’ as median or average;
  - definition of error bars as s.d. or s.e.m.

Please complete ALL of the questions below.
Select "Not Applicable" only when the requested information is not relevant for your study.

Materials

Newly Created Materials	Information included in the manuscript?	In which section is the information available? (Reagents and Tools Table, Materials and Methods, Figures, Data Availability Section)
New materials and reagents need to be available; do any restrictions apply?	Not Applicable	Reagents and Tools Table and Materials

Antibodies	Information included in the manuscript?	In which section is the information available? (Reagents and Tools Table, Materials and Methods, Figures, Data Availability Section)
For **antibodies** provide the following information:<br>- Commercial antibodies: RRID (if possible) or supplier name, catalogue number and or/clone number<br>- Non-commercial: RRID or citation	Yes	Materials and Methods section

DNA and RNA sequences	Information included in the manuscript?	In which section is the information available? (Reagents and Tools Table, Materials and Methods, Figures, Data Availability Section)
**Short novel DNA or RNA including primers, probes**: provide the sequences.	Not Applicable	

Cell materials	Information included in the manuscript?	In which section is the information available? (Reagents and Tools Table, Materials and Methods, Figures, Data Availability Section)
**Cell lines:** Provide species information, strain. Provide accession number in repository **OR** supplier name, catalog number, clone number, and/**OR** RRID.	Yes	Materials and Methods
**Primary cultures:** Provide species, strain, sex of origin, genetic modification status.	Not Applicable	
Report if the cell lines were recently **authenticated** (e.g., by STR profiling) and tested for mycoplasma contamination.	Yes	

Experimental animals	Information included in the manuscript?	In which section is the information available? (Reagents and Tools Table, Materials and Methods, Figures, Data Availability Section)
**Laboratory animals or Model organisms:** Provide species, strain, sex, age, genetic modification status. Provide accession number in repository **OR** supplier name, catalog number, clone number, **OR** RRID.	Not Applicable	
**Animal observed in or captured from the field:** Provide species, sex, and age where possible**.**	Not Applicable	
Please detail **housing and husbandry conditions**.	Not Applicable	

Plants and microbes	Information included in the manuscript?	In which section is the information available? (Reagents and Tools Table, Materials and Methods, Figures, Data Availability Section)
**Plants:** provide species and strain, ecotype and cultivar where relevant, unique accession number if available, and source (including location for collected wild specimens).	Not Applicable	
**Microbes:** provide species and strain, unique accession number if available, and source.	Not Applicable	

Human research participants	Information included in the manuscript?	In which section is the information available? (Reagents and Tools Table, Materials and Methods, Figures, Data Availability Section)
If collected and within the bounds of privacy constraints report on age, sex and gender or ethnicity for all study participants.	Not Applicable	

Core facilities	Information included in the manuscript?	In which section is the information available? (Reagents and Tools Table, Materials and Methods, Figures, Data Availability Section)
If your work benefited from core facilities, was their service mentioned in the acknowledgments section?	Yes	Methods and Acknowledgements sections

Design

Study protocol	Information included in the manuscript?	In which section is the information available? (Reagents and Tools Table, Materials and Methods, Figures, Data Availability Section)
If study protocol has been **pre-registered, provide DOI in the manuscript**. For clinical trials, provide the trial registration number **OR** cite DOI.	Not Applicable	
Report the **clinical trial registration number** (at ClinicalTrials.gov or equivalent), where applicable.	Not Applicable	

Laboratory protocol	Information included in the manuscript?	In which section is the information available? (Reagents and Tools Table, Materials and Methods, Figures, Data Availability Section)
Provide DOI OR other citation details if **external detailed step-by-step protocols** are available.	Not Applicable	

Experimental study design and statistics	Information included in the manuscript?	In which section is the information available? (Reagents and Tools Table, Materials and Methods, Figures, Data Availability Section)
Include a statement about **sample size** estimate even if no statistical methods were used.	Not Applicable	
Were any steps taken to minimize the effects of subjective bias when allocating animals/samples to treatment (e.g. **randomization procedure**)? If yes, have they been described?	Not Applicable	
Include a statement about **blinding** even if no blinding was done.	Not Applicable	
Describe **inclusion/exclusion criteria** if samples or animals were excluded from the analysis. Were the criteria pre-established?<br><br>If sample or data points were omitted from analysis, report if this was due to attrition or intentional exclusion and provide justification.	Not Applicable	
For every figure, are **statistical tests** justified as appropriate? Do the data meet the assumptions of the tests (e.g., normal distribution)? Describe any methods used to assess it. Is there an estimate of variation within each group of data? Is the variance similar between the groups that are being statistically compared?	Yes	

Sample definition and in-laboratory replication	Information included in the manuscript?	In which section is the information available? (Reagents and Tools Table, Materials and Methods, Figures, Data Availability Section)
In the figure legends: state number of times the experiment was **replicated** in laboratory.	Yes	Figure legends
In the figure legends: define whether data describe **technical or biological replicates**.	Yes	

Ethics

Ethics	Information included in the manuscript?	In which section is the information available? (Reagents and Tools Table, Materials and Methods, Figures, Data Availability Section)
Studies involving **human participants**: State details of **authority granting ethics approval** (IRB or equivalent committee(s), provide reference number for approval.	Not Applicable	
Studies involving **human participants**: Include a statement confirming that **informed consent** was obtained from all subjects and that the experiments conformed to the principles set out in the WMA Declaration of Helsinki and the Department of Health and Human Services Belmont Report.	Not Applicable	
Studies involving **human participants:** For publication of **patient photos**, include a statement confirming that consent to publish was obtained.	Not Applicable	
Studies involving experimental **animals**: State details of **authority granting ethics approva**l (IRB or equivalent committee(s), provide reference number for approval. Include a statement of compliance with ethical regulations.	Not Applicable	
Studies involving **specimen and field samples:** State if relevant **permits** obtained, provide details of authority approving study; if none were required, explain why.	Not Applicable	

Dual Use Research of Concern (DURC)	Information included in the manuscript?	In which section is the information available? (Reagents and Tools Table, Materials and Methods, Figures, Data Availability Section)
Could your study fall under dual use research restrictions? Please check biosecurity documents and list of **select agents and toxins** (CDC): https://www.selectagents.gov/sat/list.htm	Not Applicable	
If you used a select agent, is the security level of the lab appropriate and reported in the manuscript?	Not Applicable	
If a study is subject to dual use research of concern regulations, is the name of the **authority granting approval and reference number** for the regulatory approval provided in the manuscript?	Not Applicable	

Reporting

The MDAR framework recommends adoption of discipline-specific guidelines, established and endorsed through community initiatives. Journals have their own policy about requiring specific guidelines and recommendations to complement MDAR.

Adherence to community standards	Information included in the manuscript?	In which section is the information available? (Reagents and Tools Table, Materials and Methods, Figures, Data Availability Section)
State if relevant guidelines or checklists (e.g., **ICMJE, MIBBI, ARRIVE, PRISMA**) have been followed or provided.	Not Applicable	
For **tumor marker prognostic studies,** we recommend that you follow the **REMARK** reporting guidelines (see link list at top right). See author guidelines, under 'Reporting Guidelines'. Please confirm you have followed these guidelines.	Not Applicable	
For **phase II and III randomized controlled trials**, please refer to the **CONSORT** flow diagram (see link list at top right) and submit the CONSORT checklist (see link list at top right) with your submission. See author guidelines, under 'Reporting Guidelines'. Please confirm you have submitted this list.	Not Applicable	

Data Availability

Data availability	Information included in the manuscript?	In which section is the information available? (Reagents and Tools Table, Materials and Methods, Figures, Data Availability Section)
Have **primary datasets** been deposited according to the journal's guidelines (see 'Data Deposition' section) and the respective accession numbers provided in the Data Availability Section?	Yes	
Were **human clinical and genomic datasets** deposited in a public access-controlled repository in accordance to ethical obligations to the patients and to the applicable consent agreement?	Not Applicable	
Are **computational models** that are central and integral to a study available without restrictions in a machine-readable form? Were the relevant accession numbers or links provided?	Not Applicable	
If publicly available data were reused, provide the respective **data citations in the reference list**.	Not Applicable	
